# Data-informed discovery of hydrolytic nanozymes

**DOI:** 10.1038/s41467-022-28344-2

**Published:** 2022-02-11

**Authors:** Sirong Li, Zijun Zhou, Zuoxiu Tie, Bing Wang, Meng Ye, Lei Du, Ran Cui, Wei Liu, Cuihong Wan, Quanyi Liu, Sheng Zhao, Quan Wang, Yihong Zhang, Shuo Zhang, Huigang Zhang, Yan Du, Hui Wei

**Affiliations:** 1 College of Engineering and Applied Sciences, Nanjing National Laboratory of Microstructures, Jiangsu Key Laboratory of Artificial Functional Materials, Nanjing University, 210023 Nanjing, Jiangsu China; 2 Hubei Key Laboratory of Genetic Regulation and Integrative Biology, School of Life Sciences, Central China Normal University, 430079 Wuhan, Hubei China; 3 Jiangsu Key Laboratory of Druggability of Biopharmaceuticals, State Key Laboratory of Natural Medicines, School of Life Science and Technology, China Pharmaceutical University, 211198 Nanjing, Jiangsu China; 4 College of Chemistry and Molecular Sciences, Wuhan University, 430072 Wuhan, Hubei China; 5 State Key Laboratory of Electroanalytical Chemistry, Changchun Institute of Applied Chemistry, Chinese Academy of Sciences, 130022 Changchun, Jilin China; 6 University of Science and Technology of China, Hefei, 230026 Anhui, Hefei China; 7 Collaborative Innovation Center of Advanced Microstructures and Institute of Materials Engineering Nanjing University, 210093 Nanjing, Jiangsu China; 8 State Key Laboratory of Analytical Chemistry for Life Science, School of Chemistry and Chemical Engineering, Nanjing University, 210023 Nanjing, Jiangsu China; 9 Chemistry and Biomedicine Innovation Center (ChemBIC), Nanjing University, 210023 Nanjing, Jiangsu China

**Keywords:** Nanoscale biophysics, Biocatalysis, Synthesis and processing

## Abstract

Nanozyme is a collection of nanomaterials with enzyme-like activity but higher environmental tolerance and long-term stability than their natural counterparts. Improving the catalytic activity and expanding the category of nanozymes are prerequisites to complement or even supersede enzymes. However, the development of hydrolytic nanozymes is still challenged by diverse hydrolytic substrates and following complicated mechanisms. Here, two strategies are informed by data to screen and predict catalytic active sites of MOF (metal–organic framework) based hydrolytic nanozymes: (1) to increase the intrinsic activity by finely tuned Lewis acidity of the metal clusters; (2) to improve the density of active sites by shortening the length of ligands. Finally, as-obtained Ce-FMA-MOF-based hydrolytic nanozyme is capable of cleaving phosphate bonds, amide bonds, glycosidic bonds, and even their mixture, biofilms. This work provides a rational methodology to design hydrolytic nanozyme, enriches the diversity of nanozymes, and potentially sheds light on future evolution of enzyme engineering.

## Abstract (web summary)

Developing hydrolytic nanozymes remains challenging. Here the authors present a rational methodology to design hydrolytic nanozyme by developing a data-informed strategy to screen and identify potential scaffold and active sites of hydrolase-like nanozyme.

## Introduction

Exploring enzyme mimics in artificially fabricated systems is a promising strategy to overcome the instability and high cost of enzymes^1^. It can also enable us to better understand the living world^2^. However, the development of artificial enzymes is challenged by limited knowledge on miscellaneous mechanisms and finite chemical methodology to mimic. Over the last two decades, the emergence of nanotechnology has expanded artificial enzymes into nanomaterials, which are now collectively termed as nanozymes^3–7^. Nanozymes integrate multivalent catalytic sites while retaining the multifunctional repertoires of nanomaterials, such as the magnetic property of Fe_3_O_4_. Thereby diverse feats in bioanalysis^4,8,9^, medical imaging^10^, therapeutics^11^ and tissue engineering^12^ have been achieved, enriching biomimetic nanozymes to a larger context.

**Fig. 1:**
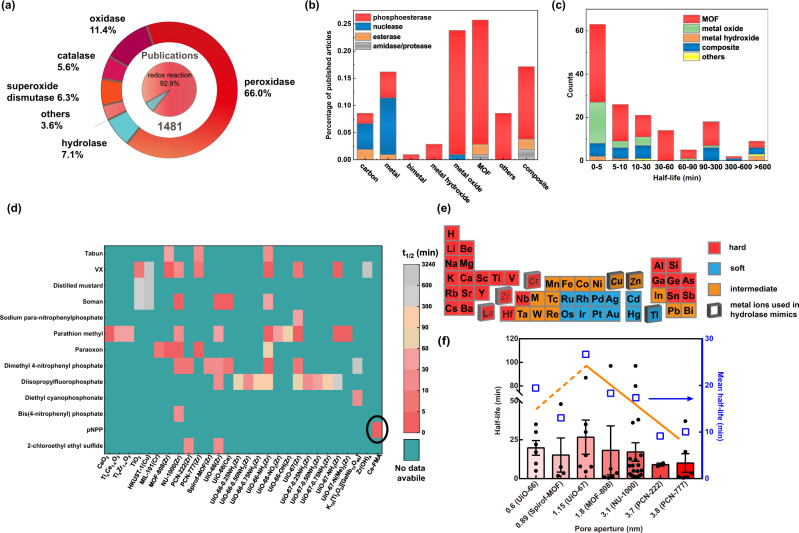
The overall statistical analysis of hydrolytic nanozymes. **a** Proportions of different types of nanozymes. Raw data were adapted from Wu et al.^3^. **b** Stacked histogram of the publication frequency distribution of studies on different types of material related to each type of hydrolase. **c** Stacked histogram of the number distribution of each material based on its half-life of phosphatase-like activity. **d** Heat map of the half-lives of various phosphatase-like nanozymes and their corresponding substrates. Each half-life is presented as the mean unless there is only one sample. The circled one represents the data-informed Ce-FMA in this work. **e** Periodic table of metal elements mapped by hard-soft-acid-base theory. The red indicates hard Lewis acid, the blue indicates soft Lewis acid and the yellow indicates the intermediate Lewis acid. Titled squares represent metal compositions that have been reported with hydrolase-like activity. **f** Half-lives of reported Zr-based MOFs versus their pore aperture; the mean values are shown as blue empty squares. The number associated with each MOF on the x-axis represents the pore/window aperture^14,40,47–51^. Each half-life is presented as the mean ± standard deviation.

In our recent review on nanozymes^3^, we summarised an exponential growth in the number of publications on nanozymes, demonstrating the fast expansion of this field. However, the breadth of enzymatic reactions that has been explored is, to date, rather limited. Specifically, a detailed analysis of these papers showed that only a small fraction (7.1%) focused on hydrolytic enzyme mimics, while the majority (92.9%) focused on redox enzyme mimics (Fig. 1a). To fully exploit nanozymes, it is demanded to widen the category that nanozymes can mimic and study them in-depth to compensate this 13-fold difference in the number of studies on hydrolase and redox enzyme mimics.

Biologically, hydrolases are involved in 13 different bio-transformations. Among them, esters (esterases), phosphoesters (phosphoesterases), amides (amidases and proteases), and carbohydrates (glycosidases) are the most common ones, see in Supplementary Table 1. The broad substrate scope of hydrolases enables them to function in nerve impulse transmission (*e.g.*, acetylcholinesterase), blood sugar balance (*e.g.*, glycogen phosphorylase), dissimilation (*e.g.*, digestive enzyme) and energy transfer (*e.g.*, ATP hydrolase). Additionally, organophosphate-like nanozymes can degrade organophosphorus pesticides/chemical warfare agents (such as soman, sarin, and tabun) by breaking the P-X bond (X = O, F, CN, *etc*.)^13^, hopeful to be embedded into protective masks. Despite the encouraging achievements, to date, only a few such nanozymes have been developed, which can be attributed, at least in part to that the current design strategies are heavily relying on integrating natural active site moieties (such as Zn^2+^-coordinated complexes) within/onto nanomaterials^5,14–19^. The specificity and regulation of natural active sites restrict the further evolution of these nanozymes in abiotic environment or specific microenvironment, which may lead to non-optimal reactivity and thus an unsuitable replication template for new-to-nature catalysis^20–22^. Moreover, the diversity of substrates and relatively limited knowledge on the catalytic mechanisms of hydrolases give additional obstacles towards the design of new hydrolytic nanozymes. To overcome these challenges and expand nanozyme functionality beyond nature’s repertoire, herein, we identified two key factors to design MOF (metal–organic framework) based hydrolytic nanozymes through a data-informed analysis of published hydrolase-like nanozymes based on our recent comprehensive review^3^.

Data-informed analysis is an approach that balances between expertise and understanding of information, thus drawing key information and further deriving insights from structured and unstructured data^23^. In this work, 105 research papers describing hydrolytic nanozymes are screened from 1481 research papers we reviewed (the list of papers we covered is supplemented as Supplementary Data 1). The breadth and the efficiency heat map of hydrolytic nanozymes (Fig. 1d) indicated MOF as a good scaffold to incorporate active sites. Further, two factors were informed through data of which the metal ions were suggested as hard Lewis acid (*i.e*., Ce^4+^) for higher affinity with substrates while the ligand was deduced as fumaric acid in that a shorter ligand could increase the density of the active sites^24^. The optimised hydrolytic MOF nanozyme was prepared experimentally and shown to possess excellent phosphatase-, protease- and glycosidase-like activities among current nanozymes. Moreover, the optimised hydrolytic nanozyme even successfully degraded biofilms under mild conditions. This work provides a general methodology to design hydrolytic nanozymes. And the abiotic active sites we derived may conversely shed a light on enzyme engineering in the future.

## Results

### Data-informed strategy to identify hydrolase-like material candidates

The course to develop a potent hydrolytic nanozyme is (1) selecting a suitable scaffold to embed/design highly active sites; (2) identifying a highly reactive site to activate hydrolytic substrates; and (3) performing experiments to fabricate and confirm the designed materials. We envisioned the suitable scaffold to embed active sites for nanozymes can be deduced from data classified by varying material category. Therefore, we first plotted the publication frequency distribution of studies on different types of material related to each type of hydrolase in Fig. 1b. Four types of hydrolase, namely, phosphoesterase, nuclease, esterase,and amidase/protease (there were no glycosidase mimics when this work was initiated in mid-2018) were sorted which were mimicked by carbon, metal, bimetal, metal hydroxide, metal oxide, MOF, composite and others. Notably, reports on MOFs, which are crystalline materials consisting of metal clusters coordinated by organic ligands^25–27^, and metal oxides were the most numerous, indicating these two as scaffold candidates to mimic hydrolases. On the other hand, half-life (t_*1/2*_, the time needed to achieve 50% conversion) which was presented as the most popular kinetic parameter among collected data, is grouped in Fig. 1c to determine which scaffold is more likely to achieve faster catalytic speed. MOFs quickly draw a specific focus because of their much higher representation in the literature than other materials with a sub-10 min half-life. Consequently, we inferred that MOFs are optimal candidates to mimic hydrolases.

Having confirmed MOFs as a potent scaffold, our next goal was to deduce highly active sites of MOFs. A kinetic heat map that displayed the half-lives of various phosphorylated substrates treated with nanozymes was shown in Fig. 1d. Intriguingly, though dozens of materials were reported to mimic hydrolase, the metal components were within six elements, namely zirconium (IV), cerium (IV), chromium (III), copper (II), zinc (II) and titanium (IV), see the tilted box in Fig. 1e. Moreover, nanozymes consisted of metal ions with hard Lewis acidy (see the Lewis acidy summarised in Supplementary Table 2) tend to have faster half-lives, such as zirconium (IV), the major (17/20) component of the hydrolytic MOFs, manifesting hard Lewis acid as effective active sites. Mechanistically, a hard Lewis acid, such as a high-valence metal ion, can easily activate a carbonyl or phosphoryl group by accepting an electron lone pair from the oxygen and drawing electron density away from the double bond, leading to a greater positive charge on—and thus increasing the electrophilicity and reactivity of—the central carbon or phosphorus (see in Supplementary Figure 1)^28,29^. Thus, Lewis acid metal ions are preferred active sites in a MOF scaffold for mimicking hydrolases, based on both an analysis of previously published data and hard-soft-acid-base theory.

Another impact factor in MOF scaffolds is the ligand which controls the connectivity, topology and spacing/density of metal clusters and moreover affects the final pore/window aperture of MOFs. Since different connectivity between the same metal ion and ligand can yield MOFs with different topology structures and pores/windows, we classified the MOFs in the abovementioned papers by their pore/window aperture instead of ligand and plotted their kinetic data (half-lives) in Fig. 1f. For MOFs with pore widths above approximately 1 nm, larger pores lead to faster reaction rates (*i.e.*, shorter half-lives) due to easier substrate diffusion into the catalytic interior of the MOF (solid yellow trend line, Fig. 1f)^28^. Below pore widths of approximately 1 nm, however, this trend is reversed. We attributed this reversed trend to the increased density of active site as shorter ligand means greater vicinity of metal clusters^24^. Of the MOFs in which this trend is observed, UiO-66 and UiO-67 are of particular interest, as these two isostructural MOFs differ only in the length of their structural ligands (shorter ligand benzene-1,4-dicarboxylic acid (BDC) in UiO-66 and longer ligand biphenyl-4,4’-dicarboxylate (BPDC) in UiO-67; Supplementary Fig. 2). In this regard, we selected fumaric acid (FMA, Supplementary Figure 3) as a candidate ligand because its length is shorter than BDC but able to form/construct a homologue of UiO-66. Modulators have been demonstrated to affect the yield^30^, degree of crystallinity^30^, morphology/size^17^ and presence of defects^29^ of the synthesised MOFs^30^, ultimately resulting in differences in the catalytic performance. Therefore, we also screened modulators. Three modulators, acetic acid (AA), formic acid (FA) and trifluoroacetic acid (TFA) (see their structures in Supplementary Fig. 4), were outlined for the similar carboxylic acid structures to that of FMA. Of note, though FMA contained UiO-66 like MOFs have been synthesised^31,32^, there currently are no available data showing that an FMA contained MOF possesses hydrolase-like activity.

Based on the above analyses, we selected high-valence, strong Lewis acid ions (Zr^4+^, Ce^4+^, and Hf^4+^, the three tetravalent metal ions with the strong Lewis acidity in Supplementary Table 2), FMA and modulators to construct a homologue MOF of UiO-66. Then, a total of 15 kinds of substrates (summarised in Supplementary Table 3) towards four different hydrolases were involved to confirm its activity.

### Optimisation of the metal ions and ligands

The protocols to fabricating Zr-FMA, Hf-FMA and Ce-FMA was described in Methods section. As shown in Supplementary Fig. 5a, crystalline Ce-FMA could be collected within 10 min at room temperature. Therefore, Ce-FMAs modulated by different modulators with varied ratios were collected after 10 min stirring (Supplementary Fig. 6), yielding MOFs with different size (Supplementary Fig. 7) as well as distinct Brunner-Emmet-Teller (BET) surface area (Supplementary Fig. 8 and Supplementary Table 6). In addition to Zr-FMA, Hf-FMA and Ce-FMA, we fabricated Zr-BDC, Hf-BDC and Ce-BDC to investigate which metal ions dominate the hydrolytic activity and whether increasing the density of active sites by shortening the length of ligand can increase the phosphatase activity towards p-nitrophenyl phosphate (pNPP) and bis-*p*-nitrophenyl phosphate (BNPP).

Having confirmed the identical phase structure of six MOFs by XRD, as shown in Supplementary Fig. 9a, we then tested phosphatase-like activity towards pNPP. Neither Hf-BDC nor Hf-FMA showed prominent phosphatase-like activity, as shown in Supplementary Fig. 9b, c, and these samples therefore served as null examples of Hf-based hydrolytic nanozymes. We then compared Zr- and Ce-based MOFs linked by FMA and BDC. Consistently, the Ce-based systems demonstrated greater catalytic activity than the Zr-based systems (Supplementary Fig. 9d, e), and nonactivated Ce-FMA demonstrated higher activity than activated Ce-BDC (Supplementary Fig. 9f), even though Ce-FMA and Ce-BDC showed similar size in Supplementary Fig. 10. Of note, the BET surface area of Ce-FMA was ~4 times smaller than that of activated Ce-BDC (120.44 m^2^/g versus 517.00 m^2^/g, respectively; Supplementary Table 6). This difference between the six MOFs, for one thing, demonstrates our hypothesis that a shorter ligand is beneficial to yield higher density to the active sites; for another, indicates that the increased Lewis acidity of Ce makes the *4f* orbital well suited to hybridise with the P-O bond and thus better stabilises the pentavalent phosphate intermediate for nucleophilic attack. Both factors collectively make the Ce-FMA as particularly well-suited for a hydrolase-like catalyst^33^.

### Characterisation of the optimal Ce-FMA-FA-20-RT

**Fig. 2:**
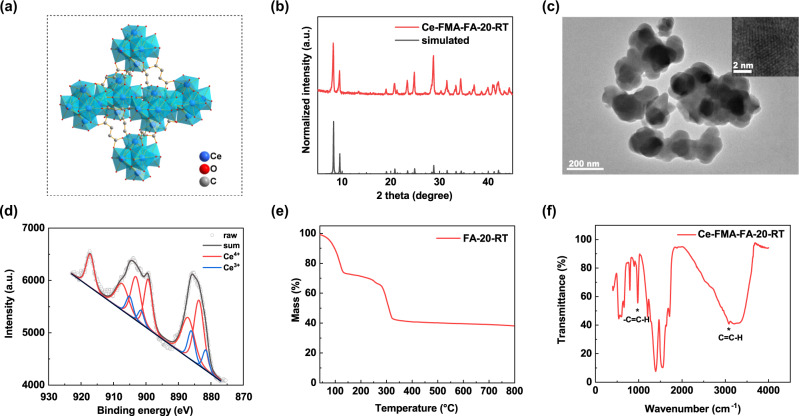
Characterisation of Ce-FMA modulated by FA with an FA-to-FMA molar ratio of 20. **a** Structure of Ce-FMA. Ce blue, O red, C grey; hydrogen atoms are omitted for clarity. **b** XRD pattern of Ce-FMA-FA-20-RT (the black lines at the bottom are simulated patterns from Lammert et al.^31^, with CCDC number 1036903). **c** TEM images of Ce-FMA-FA-20-RT at different magnifications. **d** XPS spectrum of the Ce *3d* signals of the Ce-FMA-FA-20-RT. The red curves correspond to Ce^4+^ which are deconvoluted into three Voigt doublets and the blue curves correspond to Ce^3+^ which are deconvoluted into two Voigt doublets. The black curve is summed by every deconvoluted peak. **e** TG curve of Ce-FMA-FA-20-RT analysed under air flow. **f** FTIR spectrum of Ce-FMA-FA-20-RT.

An overall comparison of the various synthetic conditions for Ce-FMA was conducted via phosphatase-like activity assays with the substrates pNPP and BNPP (Supplementary Fig. 11). Both of the mass activity and surface area-normalised activity towards pNPP and BNPP were taken into consideration to optimise the synthetic conditions of Ce-FMA (Supplementary Fig. 11b–e) since it is convenient to use mass activity to evaluate cost, but more consistent with the active sites by surface area-normalised activity. Finally, an FA-to-FMA molar ratio of 20 was determined to be the best synthetic condition according to the phosphatase-like activity, and this optimised structure was referred to as Ce-FMA-FA-20-RT. As illustrated in Fig. 2a, the [Ce_6_O_4_(OH)_4_]^12+^ clusters are arranged as cubic close packing and linked by 12 FMA^2−^, yielding the formula as [Ce_6_O_4_(OH)_4_(FMA)_6_] in Ce-FMA. The XRD patterns in Fig. 2b confirmed consistent crystal structure with simulated results^31^. TEM images revealed that this modulated MOF was ~200 nm in diameter with slight aggregation (Fig. 2c). X-ray photoemission spectroscopy (XPS) deconvolution of the peaks of Ce *3d* in Fig. 2d indicates a proportion of 82.7% Ce^4+^ and 17.3% of Ce^3+^, making the final valence of 3.8. A typical three-stage thermal behaviour determined by thermogravimetric analysis was also exhibited (Fig. 2e), suggesting a similar thermal behaviour with Ce-BDC^31^. Fourier transform infrared spectroscopy (FTIR) in Fig. 2f also demonstrates the remained trans structured C=C in MOFs derived from FMA.

### Catalytic performance

A total of 15 different substrates (listed in Supplementary Table 3) towards four main hydrolases were applied to evaluate the catalytic performance of Ce-FMA-FA-20-RT. Since the phosphoester bond is the most active bond among the above hydrolytic bonds, we studied phosphatase-like activity first and used the activity data to verify the optimal synthetic conditions (confirming the modulator and the dosage) of Ce-FMA. Then we explored Ce-FMA to hydrolyse planar amide bond in bovine serum albumin (BSA) which is more hierarchal in structures and difficult to cleave. With such success, we next investigated glycoside bonds cleavage effect in chitosan. In summary, except for carbonate esterase, Ce-FMA is able to mimic the other three types of hydrolase.

### Phosphatase-like activity

Despite the variation in all the phosphorylase substrates previously reported (summarised in Fig. 1d), the catalytic mechanism was consistent with Lewis acid-activated cleavage of the P-X bond (X = O, F, CN, *etc.*). For phosphorylated substrates, as illustrated in Supplementary Fig. 1, the reaction starts with the nucleophilic addition of an undercoordinated M-OH (M refers to metal) after the substrate was activated by metal cluster in MOF, forming a pentacoordinated phosphorus intermediate. And then, the intermediate decomposed via the elimination of alcohol^34^. Since most of the organophosphorus are neurotoxic, for analytical efficiency and safety, we selected pNPP and BNPP as phosphatase substrates instead of the other organophosphorus compounds. As shown in Supplementary Fig. 11a, both pNPP and BNPP produced yellowish 4-nitrophenol after phosphoester bond cleavage, allowing us to measure the reaction rate quantitatively by recording the absorbance at 400–405 nm. Similar to alkaline phosphatase (ALP), Ce-FMA-FA-20-RT exhibited pH-dependent activity and achieved their maximum activities at pH 10.0 (Supplementary Fig. 12). However, while the activity for BNPP hydrolysis increased with the pH, similar to that for pNPP hydrolysis, the optimised pH was 9.0, as shown in Supplementary Fig. 13a. This may be because the rate-limiting step becomes substrate binding when pH is within 9.0 to 10.0 rather than nucleophilic attack when pH is within 7.0 to 9.0^35^.

**Fig. 3:**
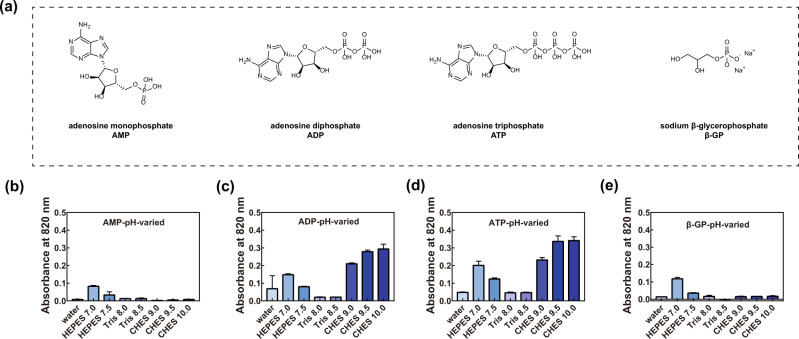
Phosphatase-like activity of Ce-FMA towards AMP, ADP, ATP and β-GP. **a** Chemical structures of AMP, ADP, ATP and β-GP. pH-dependent cleaving activity of Ce-FMA-FA-20-RT towards AMP (**b**), ADP (**c**), ATP (**d**) and β-GP (**e**). Experiments were carried out at 37 °C with 1 mM substrate and 0.1 mg/mL Ce-FMA-FA-20-RT for 12 h. Data are processed by removing the blank groups (without catalyst), and presented as the mean ± standard error, *n* = 4. The conversion rate of 12 h towards AMP, ADP ATP and β-GP under the optimised pH is summarised in Supplementary Table 7.

Since ALP can convert phosphate compounds (*i.e.*, pyrophosphate) to free phosphate, we continued testing whether Ce-FMA-FA-20-RT could cleave biological phosphates, such as AMP, ADP, ATP and β-glycerophosphate (β-GP) (see their structures in Fig. 3a). The molybdenum-blue colorimetric method^36^ was employed to investigate the hydrolysis effect by detecting the Ce-FMA-FA-20-RT-generated free phosphates, as summarised in Supplementary Figure 14. Generally, Ce-FMA-FA-20-RT exhibited higher efficiency towards ADP and ATP than AMP and β-GP, which may be ascribed to more phosphate bonds in ADP and ATP. What is more, alike tendency towards ADP and ATP was observed, manifesting a “V” shape curve. Intriguingly, though Ce-FMA-FA-20-RT showed weaker activity towards AMP and β-GP, both the substrates can still be cleaved in neutral environments (HEPES 7.0 and HEPES 7.5, see their conversion rate in Supplementary Table 7), indicating the further possible application in physiological conditions, such as phosphate prodrugs in clinics^37^ and accelerating mineralisation in hard tissue formation due to the supplementary phosphates.

Such success in colorimetric/biological phosphorylated substrates encouraged us next to investigate whether Ce-FMA-FA-20-RT could cleave bio-macromolecules with phosphate such as lipid and DNA. We chose cephalin, the second most abundant lipid in living organisms and plasmid DNA as substrates. As shown in Supplementary Figs. 15 and 16, neither of them can be cleaved by Ce-FMA-FA-20-RT. Two reasons could be rationalised: (1) both of the cephalin and plasmid are far larger than the pore aperture of Ce-FMA-FA-20-RT, therefore bringing great steric hindrance for reaction; (2) the long fatty chain in cephalin and large hydrophobic force in DNA may interfere the catalytic reaction.

In summary, this data-informed Ce-FMA-FA-20-RT has robust catalytic activity towards various phosphate molecules (*i.e.*, pNPP, BNPP, AMP, ADP, ATP and β-GP). The hydrolysis half-life of six MOFs and ALP (Supplementary Fig. 17) are summarised in Supplementary Table 8. Also, a comprehensive heat-map overviewed the hydrolysis action of reported hydrolytic materials as well as Ce-FMA in Fig. 1d. Remarkably, we did not apply the normally required co-catalyst such as polyethylenimine^33^ or *N*-ethylmorpholine^28^ for this data-informed Ce-FMA MOFs. However, the strong binding affinity between Zr/Ce and phosphate^38^ also caused the catalyst poisoning (Supplementary Fig. 18). Future efforts are encouraged to solve the recyclability dilemma for subsequent applications such as organophosphorus antidotes.

### Protease-like activity

Given the breadth of biologically and chemically relevant hydrolase reactions and mechanisms, we were curious whether Ce-FMA could mimic other hydrolytic enzymes beyond phosphoesterases. Thus, we next evaluated the catalytic performance of Ce-FMA as a protease. Hydrolysis of amide/peptide bonds in proteins is more challenging than that of phosphate bonds because the phosphate linkage is more easily cleaved. In contrast to the autolysis of phosphate bond (such as phosphodiester linkage in BNPP, as shown in Supplementary Fig. 13b), the autolysis of amide/peptide bonds has a half-life of 350 years under physiological pH and temperature^39^.

**Fig. 4:**
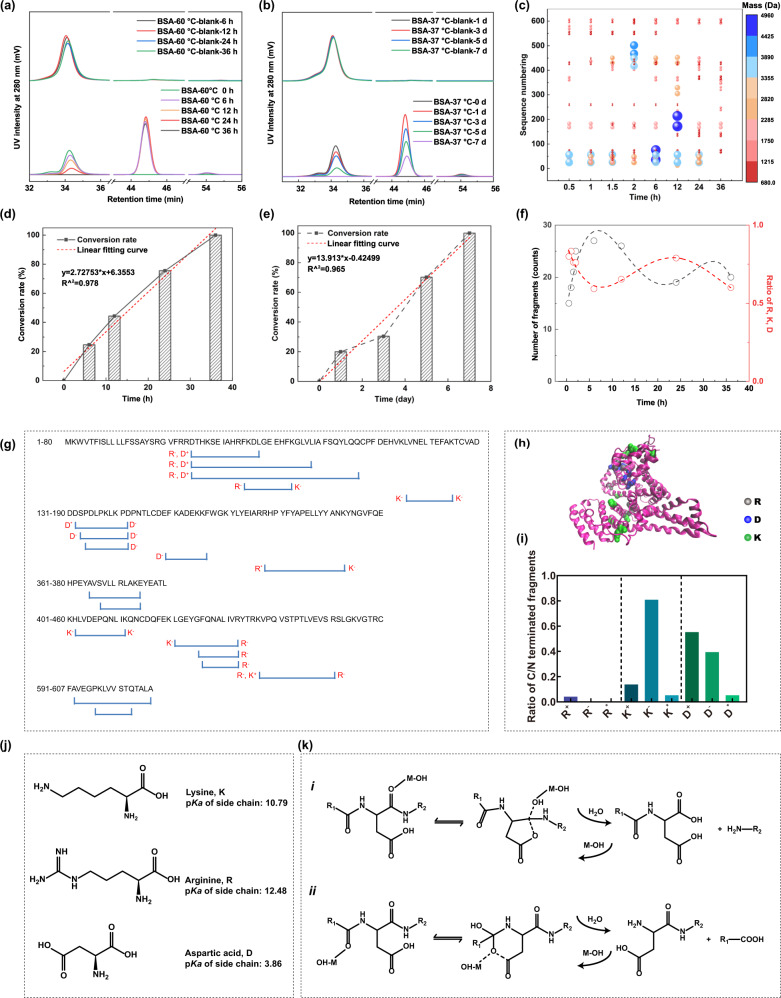
Protease-like activity of Ce-FMA-FA-20-RT. **a** GPC profiles of BSA with/without Ce-FMA-FA-20-RT in PBS (pH 7.4) at 60 °C. **b** GPC profiles of BSA with/without Ce-FMA-FA-20-RT in PBS (pH 7.4) at 37 °C. **c** Fragment numbering and mass of fragments of Ce-FMA-FA-20-RT degraded BSA collected at different times. The mass is indicated by the colour gradient and the size of the bubble. **d**, **e** Time-dependent conversion rate of BSA corresponding to **a** and **b**, which is calculated by the peak area integral of BSA. **f** The number of fragments and ratio of fragments with indicated termini versus reaction time. **g** Sequence coverage of BSA and Ce-FMA-FA-20-RT cleaved peptides after 24 h. **h** Visualisation of BSA and the surface distribution of cleaved R, K and D after 24 h at 60 °C (PDB code: 6QS9, [https://www.pdbus.org/structure/6QS9]). **i** Histogram of the frequency distribution of R, K and D. Specifically, each cleavage site is classified as N-terminus (R^+^, K^+^ and D^+^), C-terminus (R^−^, K^−^ and D^−^) or both (R^*^, K^*^ and D^*^). **j** Chemical structures of K, R and D and the p*K*_*a*_ values of their side chains. **k** Mechanisms of cleavage at D^−^ (*i*) and D^+^ (*ii*) termini, showing both of them are thermally stable to form the cyclic anhydride and imide intermediates, adapted from Li et al.^52^.

We applied bovine serum albumin (BSA) as a substrate to test the protease-like activity. BSA is a commonly used and stable globular protein with 585 amino acids (66.4 kDa molecular weight). To monitor the hydrolysis of BSA, gel permeation chromatography (GPC) with UV detection at 280 nm was used. The peak corresponding to BSA at 34–36 min decreased gradually as the reaction proceeded in PBS (pH 7.2–7.4) at both 60 °C and 37 °C, see the lower curves in Fig. 4a, b; while there is no noticeable self-degradation product in the absence of Ce-FMA-FA-20-RT, see the upper curves in Fig. 4a,b. A 100% conversion rate was achieved after 36 h and 7 d respectively at 60 °C and 37 °C in Fig. 4d, e. We then compared the hydrolysis efficiency of our MOF with trypsin (comparison was conducted at 37 °C) and MOF-808 (comparison was conducted at 60 °C) ([Zr_6_O_4_(OH_4_(BTC)_2_-(HCOO)_6_] (BTC: benzene-1,3,5-tricarboxylate) because trypsin is a typical protease while MOF-808 was reported as both good protease-like^40^ and phosphatase-like nanozyme^41^. For trypsin, a conversion rate of 100% was achieved after 24 h as shown in Supplementary Fig. 19, demonstrating seven times higher efficiency than Ce-FMA-FA-20-RT (37 °C, 7 days) but 10,000 more costly than nanozyme (Supplementary Table 9). For MOF-808, it achieved no more than 50% conversion after 24 h, while the conversion of Ce-FMA-FA-20-RT reached 75.54% (Fig. 4d and Supplementary Fig. 20c, d), even though the surface area of MOF-808 is more than eight times greater than that of Ce-FMA-FA-20-RT (1017.8893 m^2^/g versus 120.4338 m^2^/g, respectively, Supplementary Table 6), further confirming the advantage of Ce^4+^ over Zr^4+^ in the active site of hydrolytic MOFs.

However, peaks ascribed to degradation products (44–46 min and 53–55 min) were not consistent with the peak of tryptophan (referred as possible final hydrolysis products) which peaked at 93 min in Supplementary Fig. 21, indicating products with molecular weight larger than tryptophan formed. To obtain the exact molecular weight of the hydrolysed fragments and to identify possible cleavage sites, we further carried out electrospray ionization mass spectrometry (ESI-MS) analysis. As shown in Fig. 4c and Supplementary Fig. 22, during the process of hydrolysis, the molecular weight of cleaved fragments ranged from 699 to 4764 Da, composed of 6 to 41 amino acids. Notably, there were no fragments > 5000 Da even though we collected the sample at the initial process of hydrolysis (30 min), indicating only small fragments can desorb from Ce-FMA-FA-20-RT. Moreover, the fragments were finally digested into fragments with 6 to 12 amino acids at 36 h, which is alike length with the products digested by natural protease^42^. Coverage of BSA sequence by Ce-FMA-FA-20-RT cleaved peptides from 0.5 h to 36 h indicates arginine (R), lysine (K) and aspartic acid (D) are the three main cleavage sites, see Fig. 4f, g and Supplementary Fig. 22. The ratio of selectivity (number of fragments with R or K or D/number of total fragments) are plotted in Fig. 4f. Reasonably, the ratio of selectivity decreases when the number of fragments increases but increases if the number of fragments decreases. Moreover, the cleaved sites, plotted in Fig. 4h, are located on the outer surface of BSA, suggesting a crucial interaction between the outer surface of BSA and Ce-FMA-FA-20-RT which can also be proved by the protein lane of the mixed samples (Supplementary Fig. 23b) and TEM images in Supplementary Fig. 24e, f.

The high protease-like activity of Ce-FMA-FA-20-RT derives from Lewis acid activation mechanism. Similar to the activation of organophosphorus, the Lewis acid (*i.e.*, Ce^4+^) activates carbon by polarising the peptide bond after coordinating with the amide oxygen, thus enhancing the affinity for nucleophile attack to break the amide bond. We ascribed the selective cleavage on sites of R, K, and D to two reasons. First, the alkaline/acid groups in side chains accelerate the process of hydrolysis, see in Fig. 4j. Second, hydrolysis of D may undergo two pathways with cyclic anhydride or imide intermediate (Fig. 4k) which are determined by the varied microenvironment of peptides/pH^43^. Understanding the specific interaction is helpful for the future design of site-selective protease-like nanozyme and accordingly to acquire potential bioactive peptides. Moreover, since Ce-FMA-FA-20-RT showed different cleavage positions from that by trypsin (R, K, and D for Ce-FMA-FA-20-RT vs. R and K for trypsin), we further summarised a comparison between Ce-FMA-FA-20-RT and trypsin towards cost, catalytic efficiency and storage in Supplementary Table 9. In conclusion, even though trypsin showed one order of magnitude higher catalytic efficiency than Ce-FMA-FA-20-RT, this data-informed nanozyme decreased four order of magnitudes in cost, making itself an optional alternative.

### Glycosidase-like activity

**Fig. 5:**
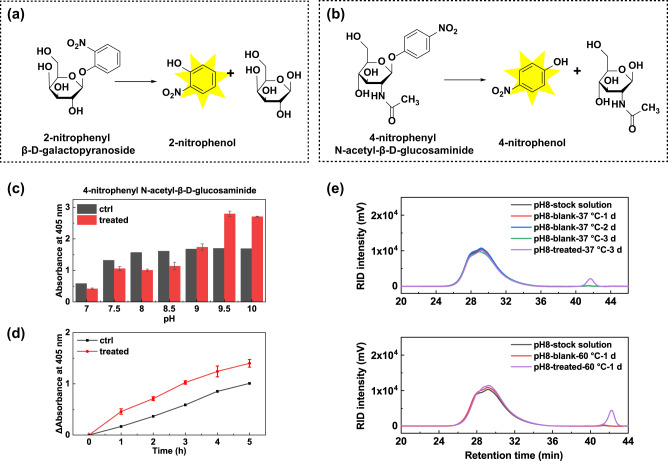
Glycosidase-like activity of Ce-FMA-FA-20-RT. **a** and **b** Schematic illustration of hydrolysis and the detection principle of 2-nitrophenyl β-d-galactopyranoside in **a** and 4-nitrophenyl *N*-acetyl-β-d-glucosaminide in **b**. **c** The optical density at 405 nm of hydrolysed product of 4-nitrophenyl *N*-acetyl-β-d-glucosaminide under different pHs at 60 °C for 8 h. Data are presented as mean ± standard error of the mean (*n* = 3). **d** Optical density change at 405 nm of hydrolysed product of 4-nitrophenyl *N*-acetyl-β-d-glucosaminide under pH 10.0 bathed at 60 °C for 5 h. (ΔAbsorbance = Absorbance_time_−Absorbance_0 h_) Data are presented as mean ± standard error of the mean (*n* = 3). **e** GPC profiles of carboxymethyl chitosan before and after treatment with Ce-FMA-FA-20-RT at 37 °C or 60 °C under pH 8.0.

The successful demonstration of the ability of Ce-FMA-FA-20-RT to cleave both phosphate bonds and peptide bonds encouraged us to further explore the hydrolytic activity of this material towards glycosidic bonds. However, glycosidase-like nanozymes are rarely studied compared with the other three hydrolase-like nanozymes. To evaluate the broad substrate scope of Ce-FMA-FA-20-RT, we started with chromogenic substrates of 2-nitrophenyl β-d-galactopyranoside and 4-nitrophenyl *N*-acetyl-β-d-glucosaminide (see their structure in Fig. 5a, b). In general, Ce-FMA-FA-20-RT is more efficient to cleave 4-nitrophenyl *N*-acetyl-β-d-glucosaminide than 2-nitrophenyl β-d-galactopyranoside (see in Fig. 5c, d and Supplementary Fig. 25, in which a higher reaction rate was observed in 4-nitrophenyl *N*-acetyl-β-d-glucosaminide). We further applied an ion chromatography to investigate whether Ce-FMA-FA-20-RT could cleave α-1,4 glycosidic bonds in maltose and β-1,4 glycosidic bonds in lactose when neither of the two substrates have an acetyl amino group. However, there were no cleaved monosaccharide products (Supplementary Fig. 26). These interesting results indicate that the hydrolysis effect on 2-nitrophenyl β-d-galactopyranoside and 4-nitrophenyl *N*-acetyl-β-d-glucosaminide is benefited by the good leaving groups (*i.e.*, 2-nitrophenyl and 4-nitrophenyl groups). Therefore, we continued choosing carboxymethyl chitosan as a substrate since the carboxymethyl groups may coordinate with Ce clusters which may help decrease the adsorption energy. GPC with a refractive index detector (RID) was used to measure the product of carboxymethyl chitosan. The results of GPC demonstrate that Ce-FMA-FA-20-RT is able to cleave carboxymethyl chitosan in alkaline (pH 8.0) environments both at 37 °C and 60 °C, see Fig. 5e. The area of the peak ascribed to product increased with the temperature enhanced and the product peak in 60 °C shifted more right than that in 37 °C (retention time 42–44 min vs. 40–42 min). Moreover, the UV intensity peak at 254 nm to product grows more sharp and intense, as outlined by the dashed blue rectangle in Supplementary Fig. 27, indicating the successful cleavage towards carboxymethyl chitosan. We also applied carboxymethyl chitosan to test the reusability of Ce-FMA-FA-20-RT. As shown in Supplementary Fig. 28, Ce-FMA-FA-20 remained 80% activity in the second recycle but decreased dramatically in the third recycle.

Generally, Ce-FMA-FA-20-RT prefers to cleaving glucuronide derivatives with (1) groups lowering the adsorption energy; (2) groups lowering the desorption energy (such as 2-nitrophenyl and 4-nitrophenyl). More universal and powerful glycosidase-like nanozyme still needs further improvement.

### Application of Ce-FMA-FA-20-RT in hydrolysis of multiple substrates for biofilm degradation

**Fig. 6:**
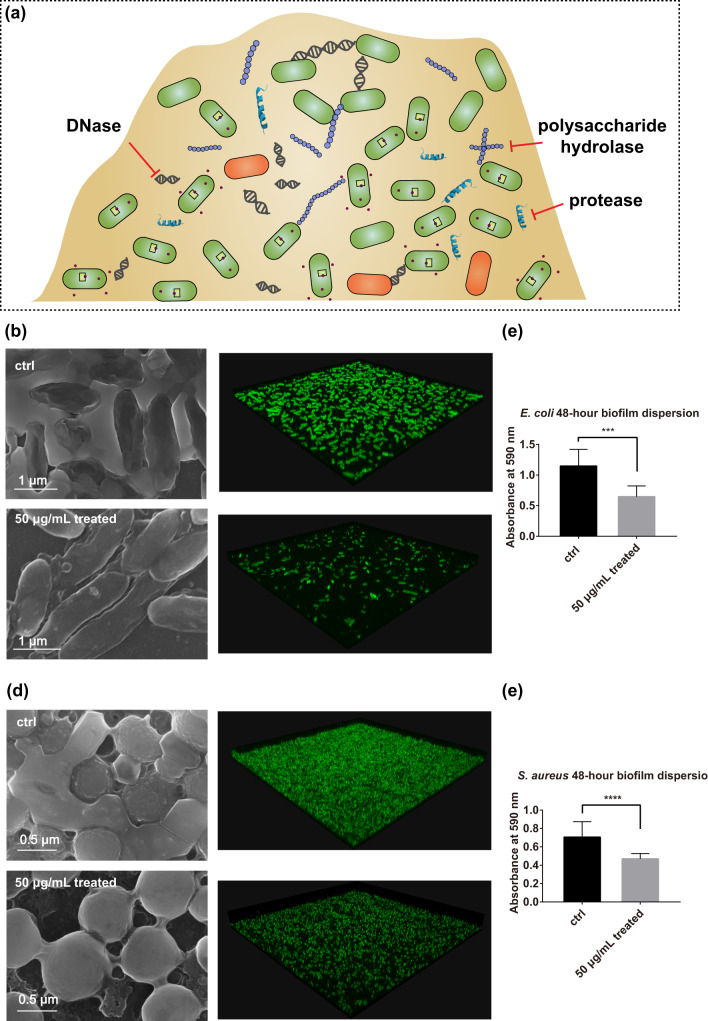
Hydrolytic performance of Ce-FMA-FA-20-RT on biofilms. **a** Schematic of the diverse biofilm components and strategies to combat biofilm formation by using hydrolytic enzymes. **b** SEM and confocal images of biofilms formed by *E. coli* without/with Ce-FMA-FA-20-RT treatment (upper: low magnification, lower: high magnification). The biofilm was stained by FilmTracer™ FM™ 1-43 Green Biofilm Cell Stain. And the confocal images were obtained using an Olympus confocal microscope FV 3000 and a ×100/1.45 NA oil immersion objective. **c** Crystal violet staining of biofilms formed by *E. coli* without/with Ce-FMA-FA-20-RT treatment (significance level: ***P < 0.001). Data are presented as the mean ± standard error after removing the maximum and minimum, *n* = 12. **d** SEM and confocal images of biofilms formed by *S. aureus* without/with Ce-FMA-FA-20-RT treatment (upper: low magnification, lower: high magnification). **e** Crystal violet staining of biofilms formed by *S. aureus* without/with Ce-FMA-FA-20-RT treatment (significance level: ****P < 0.0001). Data are presented as the mean ± standard error after removing the maximum and minimum, *n* = 16.

As described above, Ce-FMA-FA-20-RT has been shown to hydrolyse several individual substrates containing phosphoester bonds, amide/peptide bonds and glycosidic bonds. A more challenging feat is the degradation of mixture of these substrates, such as biofilms. A biofilm is a microbial consortium with self-produced extracellular polymeric substances (EPS). Inside the three-dimensional architecture of a biofilm, the EPS forms a scaffold that hosts the bacteria and is responsible for external defence, adhesion to surfaces, connectivity and nutrient trapping. Due to the roles that a biofilm plays as a protector and energy supplier, cells in a biofilm have adopted properties different from those of planktonic bacteria, thus limiting the efficacy of antimicrobials against bacterial infection involving biofilms^44^. One of the reasons why biofilms are so difficult to deal with is their varied compositions: polysaccharides/phosphoethanolamine cellulose^45^, proteins, nucleic acids and lipids (Fig. 6a). This complexity increases the difficulty for a single enzyme to disrupt a biofilm. In this regard, Ce-FMA-FA-20-RT, with multiple hydrolytic activities, may be advantageous, as it has been proven to be effective in cleaving phosphoester bonds, amide/peptide bonds and glycosidic bonds, which all exist in biofilms in various forms.

To test this hypothesis, we applied two representative biofilms formed by gram-negative bacteria (*E. coli*) and gram-positive bacteria (*S. aureus*). Bacterial cells were grown for 48 h in a 24-well plate stationarily to form biofilms. Then, Ce-FMA-FA-20-RT together with fresh medium was added, followed by another 12 h incubation at 37 °C to allow catalytic hydrolysis of the biofilms. Both of the scanning electronic microscope (SEM) images and confocal laser scanning microscope (CLSM) images in Fig. 6b, d display the expected thick, bulk-like adhesions between cells when no Ce-FMA-FA-20-RT treatment was applied, demonstrating the successful formation of biofilms. In contrast, the adhesion among cells became tenuous and fibre-like after Ce-FMA-FA-20-RT treatment, and clear gaps were also observed. Crystal violet staining assay was applied to semi-quantitate the amount of biofilm. As shown in Fig. 6c, e, a significant difference was observed before and after Ce-FMA-FA-20-RT treatment in both gram-negative bacteria (*E. coli*) and gram-positive bacteria (*S. aureus*). Moreover, plates spread with Ce-FMA-FA-20-RT-treated *E. coli* and *S. aureus* indicated negligible adverse effects on bacterial growth, as shown in Supplementary Fig. 29. This finding is also consistent with the intact cell morphology shown in Fig. 6b, d after treatment with Ce-FMA-FA-20-RT, confirming that the decrease in biofilm formation could only be attributed to hydrolysis rather than to bacterial cell death.

## Discussion

In conclusion, we introduced a data-informed approach to discover a high-performance hydrolytic nanozyme by systematically analysing 105 published papers to supplement the under-developed field of research on hydrolytic nanozymes. Analysis of these data indicates that MOF is a good scaffold to embed hydrolytic active sites for their tuneable metal clusters and ligands. Further structured data suggests that Lewis acidity of metal clusters in MOFs and the density of active sites which is adjusted by ligand are two critical elements. Consequently, we screened Ce^4+^ as a promising metal ion component and applied a short ligand, FMA, to construct a UiO-66–like MOF.

Encouragingly, this rationally designed Ce-FMA was highly efficient in hydrolysing a broad scope of substrates and exhibited huge promise to be further developed. First, nonactivated Ce-FMA exhibited excellent phosphatase-like activity (half-life of < 2 min) even without the use of co-catalysts. Second, Ce-FMA showed high activity towards BSA hydrolysis, with 12.7 times better efficiency than the multi-functional Zr-based MOF-808. The new cleavage sites (such as D sites) brought by nanozymes expand the diversity of peptides and offer additional probability to obtain active peptides. Third, we evaluated the ability of this Ce-FMA MOF to cleave glycosidic bonds in selected substrates. Last, we applied Ce-FMA to a mixture of biomacromolecules, *i.e.*, biofilms, from both gram-negative bacteria (*E. coli*) and gram-positive bacteria (*S. aureus*), which is hopefully applied in biomedicine and marine. Additionally, this data-informed approach can be applied to deduce abiotic active sites rather than being limited to the direct modification of natural active sites.

We acknowledge that more chemical information about hydrolytic MOF nanozymes should be analysed, such as the topology of the coordination network, pore aperture to the substrates and the modification of functional groups. However, due to the limited amount of available data, the current study was not suitable for machine learning or chemoinformatic models with multiple variables and was unable to discover esterase mimics. Nevertheless, given the rapid development of this field, we anticipate that machine learning as well as other artificial intelligence techniques will be further leveraged for the design and discovery of new nanozymes, such as powerful esterase mimics for degradation of PET (polyethylene terephthalate) in the near future.

## Methods

### Synthesis of Zr-FMA

Zr-FMA was synthesised as previously reported^46^. For details, FMA (116 mg, 1 mmol) and ZrOCl_2_·8H_2_O (322.25 mg, 1 mmol) were suspended in 10 mL of water/formic acid mixed solution (water, 9.8 mL; formic acid, 0.2 mL) and heated to 105 °C, refluxing for 24 h. The obtained white powder product was centrifuged (4100×*g*, 1 min) and washed with water for three times. The obtained solids were dried in a vacuum oven at 60 °C overnight.

### Synthesis of Hf-FMA

Hf-FMA was synthesised using the same method as Zr-FMA, except changing ZrOCl_2_·8H_2_O into HfCl_4_ (320 mg, 1 mmol).

### Synthesis of Ce-FMA

Typically, FMA (1.74 g, 15 mmol) and Ce(NH_4_)_2_(NO_3_)_2_ (1.6447 g, 15 mmol) were suspended in 30 mL solution with water and modulators (see their ratio in Supplementary Table 5), stiring for 10 min at room temperature. The yellowish solution was centrifuged (4100×*g*, 1 min) and washed with water and ethanol three times. Then, the obtained solids were dried in vacuum oven at 60 °C overnight.

### Synthesis of UiO (University of Oslo)-66 (Zr)

BDC (124.5 mg, 0.75 mmol) and ZrOCl_2_·8H_2_O (241.69 mg, 0.75 mmol) were suspended in 30 mL of DMF/formic acid mixed solution (DMF, 27 mL; formic acid, 3 mL). After sonication for 5 min, the mixture was transferred into a 50 mL Teflon-lined autoclave and heated to 120 °C for 24 h. The obtained white powder product was centrifuged (4100 × *g*, 3 min) and washed with water twice and then with acetone twice. Then the obtained solids were dried in vacuum oven at 60 °C overnight.

### Synthesis of UiO-66 (Hf)

BDC (124.5 mg, 0.75 mmol) and HfCl_4_ (240 mg, 0.75 mmol) were suspended in 30 mL of DMF/formic acid mixed solution (DMF, 27 mL; formic acid, 3 mL). After sonication for 5 min, the mixture was transferred into a 50 mL Teflon-lined autoclave and heated to 123 °C for 40 h. The obtained white powder product was centrifuged (4100×*g*, 3 min) and washed with water twice and then with acetone twice. Then the obtained solids were dried in vacuum oven at 60 °C overnight.

### Synthesis of UiO-66 (Ce)

UiO-66 (Ce) was synthesised as previously reported with minor modification^31,33^. In detail, an ultrapure water solution of Ce(NH_4_)_2_(NO_3_)_6_ (0.533 M, 2.4 mL) was added into 7.5 mL of DMF solution containing 212.4 mg BDC. The mixture was then refluxed in an oil bath at 102 °C for 15 min. Product was then centrifuged (2010×*g*, 1 min) and resuspended in fresh DMF for 1 h. After 3 cycles of centrifugation and soaking in fresh DMF for 1 h, the solids were washed by ethanol for three times. Finally, the yellowish powder product was placed in vacuum oven at 60 °C overnight to remove solvent. To activate UiO-66, the solid was soaked in ethanol overnight to fully exchange solvent and then collected by centrifuged (4100 × g, 3 min) and dried at 100 °C in vacuum oven for more than 12 h.

### Synthesis of MOF-808

MOF-808 was synthesised as previously reported^40^. In details, H_3_BTD (157.2 mg, 0.75 mmol) along with ZrOCl_2_.8H_2_O (241.8 mg, 0.75 mmol) was added into the mixture solution of 30 mL of DMF and 30 mL FA in a 100 mL flask. The solution was then refluxed in an oil bath at 130 °C for 48 h. Product was washed by DMF for one time and then washed by ethanol for several times. Finally, the white powder product was placed in vacuum oven at 70 °C overnight to remove solvent.

### Optimisation on synthetic conditions of Ce-FMAs by comparing ALP-like activity

Typically, the activity assays were conducted in the optimised pH 10.0 with a fixed concentration of 10 mM pNPP and 0.5 mg/mL catalyst prepared at different conditions with 8 parallel samples. After incubation for 20 min at 37 °C, the absorbance at 400 nm was recorded by Tecan Pro 200 Microplate Reader for mass activity comparison. The specific activity comparison was normalised by BET surface area in Supplementary Table 6. Due to the presence of high-energy phosphate bond in pNPP, all the data have been treated to subtract the autolysis of pNPP and the background of catalysts. The maximum and minimum were removed when plotting.

### Optimisation on synthetic conditions of Ce-FMA by comparing catalytic activity

The optimisation of synthetic conditions was conducted by monitoring the absorbance at 400 nm. Typically, the activity assay was conducted in the optimised pH 9.0 with a fixed concentration of 0.8 mM BNPP and 0.5 mg/mL catalyst prepared at different conditions with at least 3 parallel samples. After incubation for 3 h at 37 °C, the absorbance at 400 nm was recorded by Tecan Pro 200 Microplate Reader for mass activity comparison. The specific activity comparison was normalised by BET surface area in Supplementary Table 6. Due to the presence of high-energy phosphate bond in BNPP, all the data have been treated to subtract the autolysis of BNPP and the background of catalysts.

### GPC monitor of the BSA degradation process

BSA (66.4 kDa) was chosen as a model to test whether Ce-FMA-FA-20-RT could cleavage peptide bonds. Typically, 2 mg/mL BSA mixed with 5 mg/mL Ce-FMA-FA-20-RT in 1×PBS buffer was bathed at 60 °C or 37 °C with stirring at 1200 rpm. Equivalent reaction mixture was taken at 6 h, 12 h, 24 h, 36 h and 48 h for 60 °C group and 1 d, 3 d, 5 d and 7 d for 37 °C group. The mixture was centrifuged and supernatant was collected and diluted 4 times before being filtered with 0.22 µm filter. GPC profiles were monitored by UV detector at 280 nm. The conversion rate was defined by the decreased area on the peak of original BSA. The conversion rate *µ* could be described as follow:

1 $$\mu =1{{{-}}}\frac{{{\mbox{area}}}\; {{\mbox{of}}}\; {{\mbox{BSA}}} \;{{\mbox{after}}}\; {{\mbox{hydrolysis}}}}{{{\mbox{area}}}\; {{\mbox{of}}}\; {{\mbox{BSA}}}\; {{\mbox{before}}}\; {{\mbox{hydrolysis}}}}\times 100 \%$$

### ESI-MS characterisation on hydrolytic fragments of BSA

Hydrolysed products taken out at 0.5 h, 1.0 h, 1.5 h, 2.0 h, 6.0 h, 12 h, 24 h and 36 h were then centrifuged to collect the supernatants. Before test, samples were diluted to 10 times by ultra-pure water and removed salt by stage tip, then lyophilised and re-suspended in water for ESI-MS.

### Hydrolytic effect on β-glycosidic bond

2-nitrophenyl β-d-galactopyranoside was chosen to study the cleavage effect in different buffers. Typically, 200 µL of solutions containing 5 mM 2-nitrophenyl β-d-galactopyranoside and 1 mg/mL Ce-FMA-FA-RT-20 were incubated in different buffers at 60 °C for 8 h. Three parallel samples were conducted each time. After 8 h, 180 µL of the solution was added into 96-well plate and measured by Tecan Pro 200 Microplate Reader at 420 nm.

### Hydrolytic effect on β-*N*-acetyl-glycosidic bond

4-nitrophenyl *N*-acetyl-β-d-glucosaminide was chosen to study the cleavage effect in different buffers. Typically, 200 µL of solutions containing 5 mM 4-nitrophenyl *N*-acetyl-β-d-glucosaminide and 1 mg/mL Ce-FMA-FA-RT-20 were incubated in different buffers at 60 °C for 8 h. Three parallel samples were conducted each time. After 8 h, 180 µL of the solution was added into 96-well plate and measured by Tecan Pro 200 Microplate Reader at 405 nm.

### Hydrolysis of β-1–4 glycosidic bond (carboxymethyl chitosan as substrate in alkaline environment)

Since carboxymethyl chitosan tends to dissolve in neutral or alkaline solution, carboxymethyl chitosan was chosen to study the degradation effect in alkaline (Tris-HCl buffer at 8.0) environment. Typically, 1 mL of 1.2%_wt_ carboxymethyl chitosan (dissolved in buffer) was mixed with 200 µL of 10 mg/mL Ce-FMA-FA-20-RT in constant temperature oscillator at 37 °C with shaking (220 rpm). Three parallel samples were conducted each time. After 24 h, the mixture was centrifuged and then filtered with 0.22 μm filter for GPC test by RID detector and Pullulan was applied as an internal reference (P5 for carboxymethyl chitosan, respectively).

### SEM observation on the dispersion effect of formed biofilm

In all, 24-well plate covered with Nest circle microscope cover glass was used to incubate bacteria. After 48 h development of biofilm, 2 mL of culture medium with or without 50 µg/mL Ce-FMA-FA-20-RT was added to study whether Ce-FMA-FA-20-RT could hydrolyse existed biofilm at 37 °C. After 12 h, the medium was discarded and the wells were washed by PBS three times gently to remove suspended bacteria. Remained biofilm was then fixed by methanol for 15 min. Next, the cover glass was sputtered with Cr for subsequent SEM observation.

### CLSM observation on the dispersion effect of formed biofilm

Biofilm was cultured in coverglass bottom dish (10 mm) for 48 h and then treated with/without the addition of 50 µg/mL Ce-FMA-FA-20 for 12 h at 37 °C. Then, the medium was discarded and the wells were washed by PBS three times gently to remove suspended bacteria. The biofilm was stained by FilmTracer™ FM™ 1-43 Green Biofilm Cell Stain. And the confocal images were obtained using an Olympus confocal microscope FV 3000 and a ×100/1.45 NA oil immersion objective.

### Crystal violet staining of biofilm

The biofilm dispersion was similar as above without covering Nest circle microscope cover glass in 24-well plate. After fixation, wells were stained with 150 µL of 0.1%_wt_ crystal violet for 10 min and then washed with 200 µL of water to remove floating colour. After drying, 250 µL of ethanol was used to elute the crystal violet of each well. The stained solution was diluted moderately in 96-well plate for further measure at the absorbance at 590 nm by Tecan Pro 200 Microplate Reader.

## Supplementary information

Supplementary Information

Description of Additional Supplementary Files

Supplementary Data 1

## Data Availability

The data generated in this study are provided in the Supplementary Information/Source Data file. Source data are provided with this paper.

## Source data

Source Data
