# Supplementary material for: Data-informed discovery of hydrolytic nanozymes
Source: Nat Commun. 2022 Feb 11;13:827. doi: 10.1038/s41467-022-28344-2 (PMC8837776; doi:10.1038/s41467-022-28344-2)
Supplement: Supplementary file 1 — Supplementary Information [file 41467_2022_28344_MOESM1_ESM.docx]

**Data-informed discovery of hydrolytic nanozymes**

Sirong Li^1^, Zijun Zhou^1^, Zuoxiu Tie^1^, Bing Wang^2^, Meng Ye^3^, Lei Du^4^, Ran Cui^4^, Wei Liu^3^, Cuihong Wan^2^, Quanyi Liu^5, 6^, Sheng Zhao^1^, Quan Wang^1^, Yihong Zhang^1^, Shuo Zhang^1, 7^, Huigang Zhang^1, 7^, Yan Du^5,6^, Hui Wei^1, 8, 9*^

^1^College of Engineering and Applied Sciences, Nanjing National Laboratory of Microstructures, Jiangsu Key Laboratory of Artificial Functional Materials, Nanjing University, Nanjing, Jiangsu 210023, China.

^2^Hubei Key Laboratory of Genetic Regulation and Integrative Biology, School of Life Sciences, Central China Normal University, Wuhan, Hubei 430079, China.

^3^Jiangsu Key Laboratory of Druggability of Biopharmaceuticals, State Key Laboratory of Natural Medicines, School of Life Science and Technology, China Pharmaceutical University, Nanjing, Jiangsu 211198, China.

^4^College of Chemistry and Molecular Sciences, Wuhan University, Wuhan, Hubei 430072, China.

^5^State Key Laboratory of Electroanalytical Chemistry, Changchun Institute of Applied Chemistry, Chinese Academy of Sciences, Changchun, Jilin 130022, China.

^6^University of Science and Technology of China, Hefei, Anhui, Hefei 230026, China.

^7^Collaborative Innovation Center of Advanced Microstructures and Institute of Materials Engineering, Nanjing University, Nanjing, Jiangsu 210093, China.

^8^State Key Laboratory of Analytical Chemistry for Life Science, School of Chemistry and Chemical Engineering, Nanjing University, Nanjing, Jiangsu 210023, China.

^9^Chemistry and Biomedicine Innovation Center (ChemBIC), Nanjing University, Nanjing, Jiangsu 210023, China.

E-mail: [weihui@nju.edu.cn](mailto:weihui@nju.edu.cn)

**Supplementary** **information**

**Table of contents**

[Supplementary Figures 5](#_Toc89962769)

[Supplementary Figure 1. General mechanism for the catalytic hydrolysis of a phosphate bond. 5](#_Toc89962770)

[Supplementary Figure 2. Structures of ligands to synthesize hydrolase-like MOFs. 6](#_Toc89962771)

[Supplementary Figure 3. Structure of fumaric acid (FMA). 7](#_Toc89962772)

[Supplementary Figure 4. Structures and p*K*_a_ values in aqueous solution of selected monobasic carboxylic acids in Supplementary Table 4. 9](#_Toc89962773)

[Supplementary Figure 5. Phase confirmation of Ce-FMAs synthesized at different time without modulator and comparison of their ALP-like activity with pNPP as the substrate. 10](#_Toc89962774)

[Supplementary Figure 6. Ce-FMAs modulated by AA, FA and TFA with various ratios. 11](#_Toc89962775)

[Supplementary Figure 7. TEM images of Ce-FMAs at different magnification synthesized under various conditions. 12](#_Toc89962776)

[Supplementary Figure 8. N_2_ isotherms at 77 K of Ce-FMAs synthesized under various conditions. (a)-(j) Ce-FMAs synthesized with AA. (k)-(t) Ce-FMAs synthesized with FA. (u)-(z) Ce-FMAs synthesized with TFA. 13](#_Toc89962777)

[Supplementary Figure 9. Phase confirmation of UiO-66 structured MOFs linked by BDC or FMA, and comparison of ALP-like activity with pNPP as the substrate. 14](#_Toc89962778)

[Supplementary Figure 10. SEM/TEM images of six MOFs indicated by data. 15](#_Toc89962779)

[Supplementary Figure 11. Phosphatase-like activity of Ce-FMAs towards pNPP and BNPP. 16](#_Toc89962780)

[Supplementary Figure 12. Optimization pH of reaction medium and concentration of pNPP. 17](#_Toc89962781)

[Supplementary Figure 13. pH dependent hydrolytic activity of Ce-FMA-FA-20-RT towards BNPP and the spontaneous hydrolysis of BNPP under different pH or concentrations. 18](#_Toc89962782)

[Supplementary Figure 14. Molybdenum-blue colorimetric approach for detection of free phosphates. 19](#_Toc89962783)

[Supplementary Figure 15. Phosphatase-like activity of Ce-FMA-FA-20-RT towards cephalin. 20](#_Toc89962784)

[Supplementary Figure 16. Horizontal electrophoresis of DNA treated by Ce-FMA-FA-20-RT. 21](#_Toc89962785)

[Supplementary Figure 17. Absorbance at 405 nm of 4-nitrophenol for monitoring the catalytic hydrolysis of 10 mM pNPP by different concentrations of ALP in pH 10.0 at 37 ºC. 22](#_Toc89962786)

[Supplementary Figure 18. The recyclability of Ce-FMA-FA-20-RT towards pNPP. 23](#_Toc89962787)

[Supplementary Figure 19. Coomassie blue stained SDS-PAGE of 2 mg/mL BSA hydrolyzed by 1 mg/mL trypsin at 37 °C in 1×PBS buffer. Samples were collected at different time. 24](#_Toc89962788)

[Supplementary Figure 20. Characterization of MOF-808 and its protease-like activity towards BSA at 60 °C in PBS. 25](#_Toc89962789)

[Supplementary Figure 21. GPC profiles of BSA and tryptophan. 26](#_Toc89962790)

[Supplementary Figure 22. Sequence coverage of BSA and Ce-FMA-FA-20-RT cleaved peptides. 30](#_Toc89962791)

[Supplementary Figure 23. The adsorption behavior of protein onto Ce-FMA-FA-20-RT. 31](#_Toc89962792)

[Supplementary Figure 24. TEM images of Ce-FMA-FA-20-RT before and after reaction with various substrates. 32](#_Toc89962793)

[Supplementary Figure 25. pH dependent cleavage ability towards 2-nitrophenyl β-D-galactopyranoside. 33](#_Toc89962794)

[Supplementary Figure 26. Ion chromatography curves of disaccharides with α/β-glycosidic bonds and their monosaccharides. 34](#_Toc89962795)

[Supplementary Figure 27. GPC profiles (detected by RID and UV) of carboxymethyl chitosan before (a) and after treatment (b) with Ce-FMA-FA-20-RT at 60 °C under pH 8.0 for 26.5 h. 35](#_Toc89962796)

[Supplementary Figure 28. Reusability of carboxymethyl chitosan by Ce-FMA-FA-20-RT. 36](#_Toc89962797)

[Supplementary Figure 29. Bacteria spread on agar plates treated with/without Ce-FMA-FA-20-RT. 37](#_Toc89962798)

[Supplementary Tables 38](#_Toc89962799)

[Supplementary Table 1. Classification and schematic reaction of natural hydrolases. 38](#_Toc89962800)

[Supplementary Table 2. Absolute hardness of selected metal ions^11-13^. 39](#_Toc89962801)

[Supplementary Table 3. Hydrolytic effect of Ce-FMA-FA-20-RT towards broad substrate scope of different hydrolases. 40](#_Toc89962802)

[Supplementary Table 4. Summary of modulator used in hydrolase-like MOFs. 42](#_Toc89962803)

[Supplementary Table 5. The amount of modulator added to synthesize Ce-FMA 43](#_Toc89962804)

[Supplementary Table 6. Summary of BET surface area of Ce-FMAs and other MOFs 44](#_Toc89962805)

[Supplementary Table 7. Summary of conversion rate after 12 h towards various phosphate substrates 45](#_Toc89962806)

[Supplementary Table 8. Summary of half-life towards different MOFs and ALP 46](#_Toc89962807)

[Supplementary Table 9. Comparison between trypsin and Ce-FMA-FA-20-RT 47](#_Toc89962808)

[Supplementary Discussion 48](#_Toc89962809)

[Supplementary discussion 1 48](#_Toc89962810)

[Supplementary discussion 2 48](#_Toc89962811)

[Supplementary discussion 3 48](#_Toc89962812)

[Supplementary discussion 4 48](#_Toc89962813)

[Supplementary discussion 5 48](#_Toc89962814)

[Supplementary discussion 6 48](#_Toc89962815)

[Supplementary discussion 7 48](#_Toc89962816)

[Supplementary discussion 8 49](#_Toc89962817)

[Supplementary discussion 9 49](#_Toc89962818)

[Supplementary discussion 10 49](#_Toc89962819)

[Supplementary discussion 11 49](#_Toc89962820)

[Supplementary discussion 12 49](#_Toc89962821)

[Supplementary discussion 13 49](#_Toc89962822)

[Supplementary Methods 50](#_Toc89962823)

[Chemical and biological reagents 50](#_Toc89962824)

[Instrumentation 50](#_Toc89962825)

[Hydrolysis of phosphate monoester bond (ALP-like activity, pNPP as the substrate) 50](#_Toc89962826)

[Hydrolysis of phosphodiester bond (phosphodiesterase-like activity, BNPP as the substrate) 51](#_Toc89962827)

[Hydrolysis of lipid (cephalin) 51](#_Toc89962828)

[Hydrolysis of plasmid DNA (plasmid extracted from *E. coli*) 51](#_Toc89962829)

[Hydrolysis of lactose and maltose 51](#_Toc89962830)

[Bacterial culture and development of biofilm 52](#_Toc89962831)

[Cytotoxicity test of Ce-FMA-FA-20-RT treated *E. coli* & *S. aureus* 52](#_Toc89962832)

[Supplementary References 53](#_Toc89962833)

# Supplementary Figures


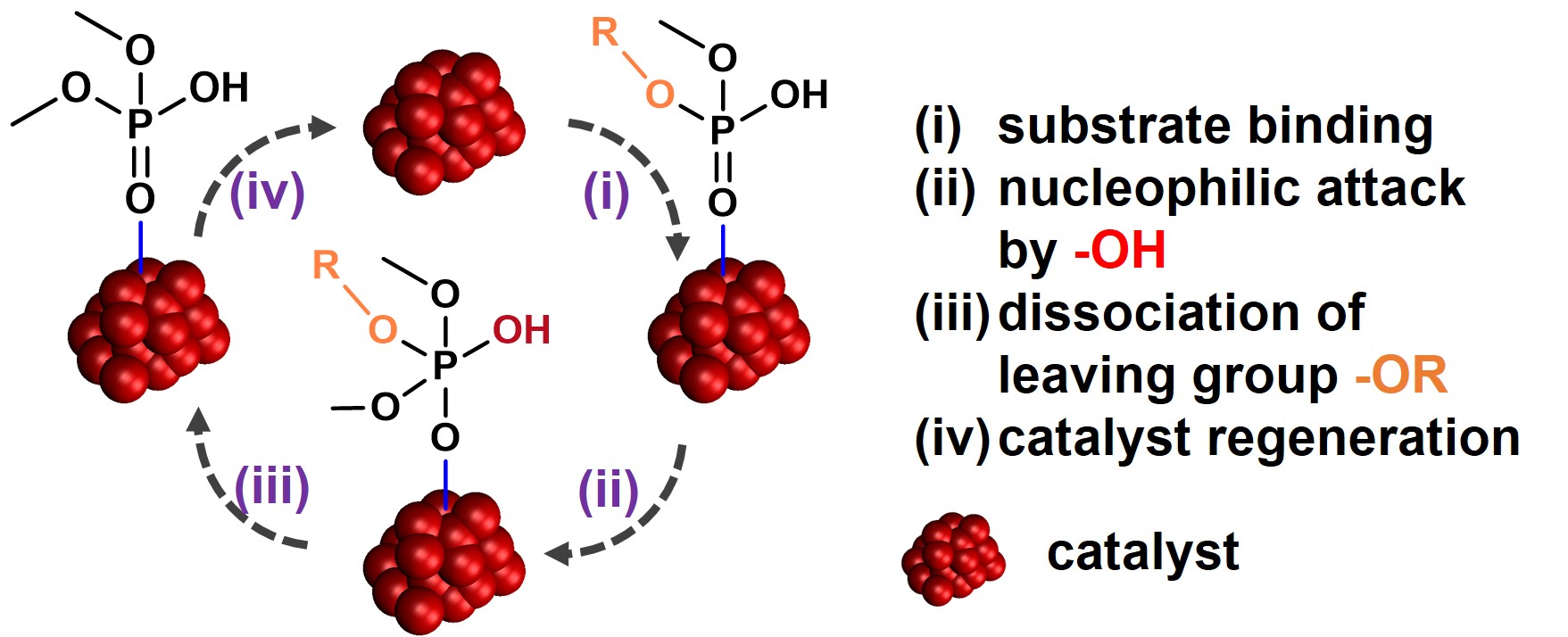


## Supplementary Figure 1. General mechanism for the catalytic hydrolysis of a phosphate bond. Adapted with permission from Katz, M. J.; Klet, R. C.; Moon, S. Y.; Mondloch, J. E.; Hupp, J. T.; Farha, O. K. ACS Catal. 2015, 5 (8), 4637-4642 (Ref 1). Copyright 2015 American Chemical Society.


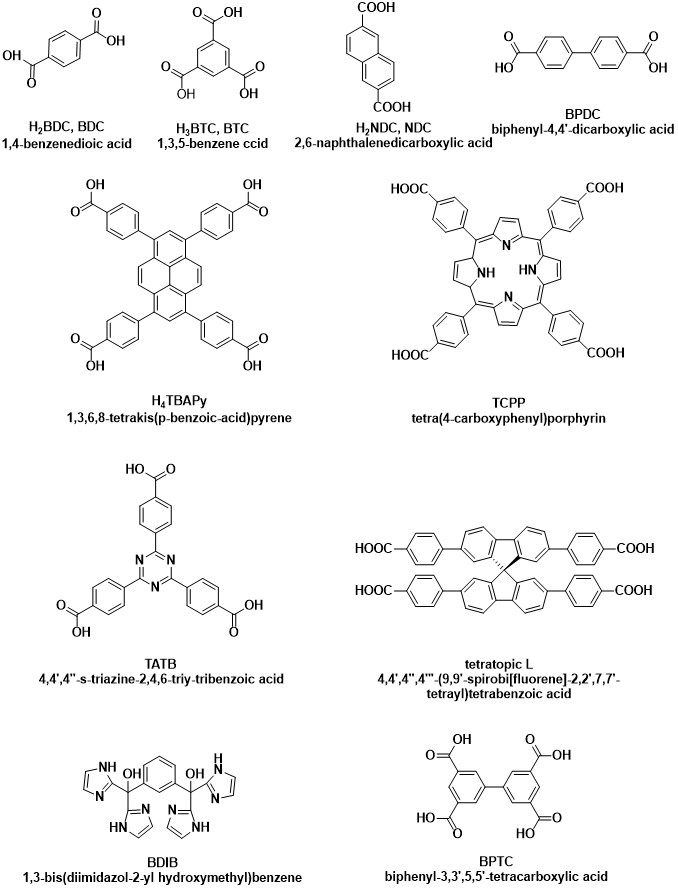


## **Supplementary Figure 2. Structures of ligands to synthesize hydrolase-like MOFs.** Specifically, BDC for UiO-66^2^ and MIL-101(Cr)^3^; BTC for HKUST-1^4^ and MOF-808^2^; NDC for DUT-84^2^; BPDC for UiO-67^5^; H_4_TBAPy for NU-1000^6^; TCPP for PCN-222^6^; TATB for PCN-777^7^; tetratopic L for Spirof-MOF^8^; BDIB and BPTC for SNNU-101(Zn)^9^.


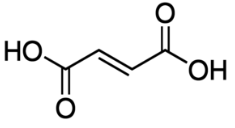


## Supplementary Figure 3. Structure of fumaric acid (FMA).


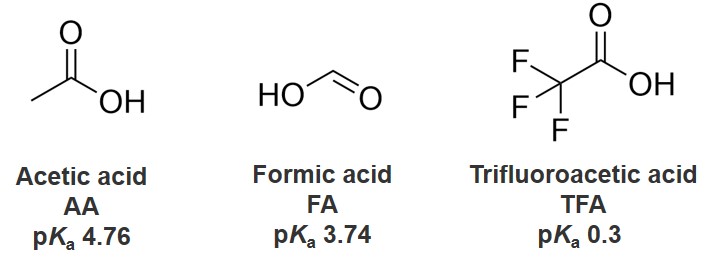


## Supplementary Figure 4. Structures and p*K*_a_ values in aqueous solution of selected monobasic carboxylic acids in Supplementary Table 4.


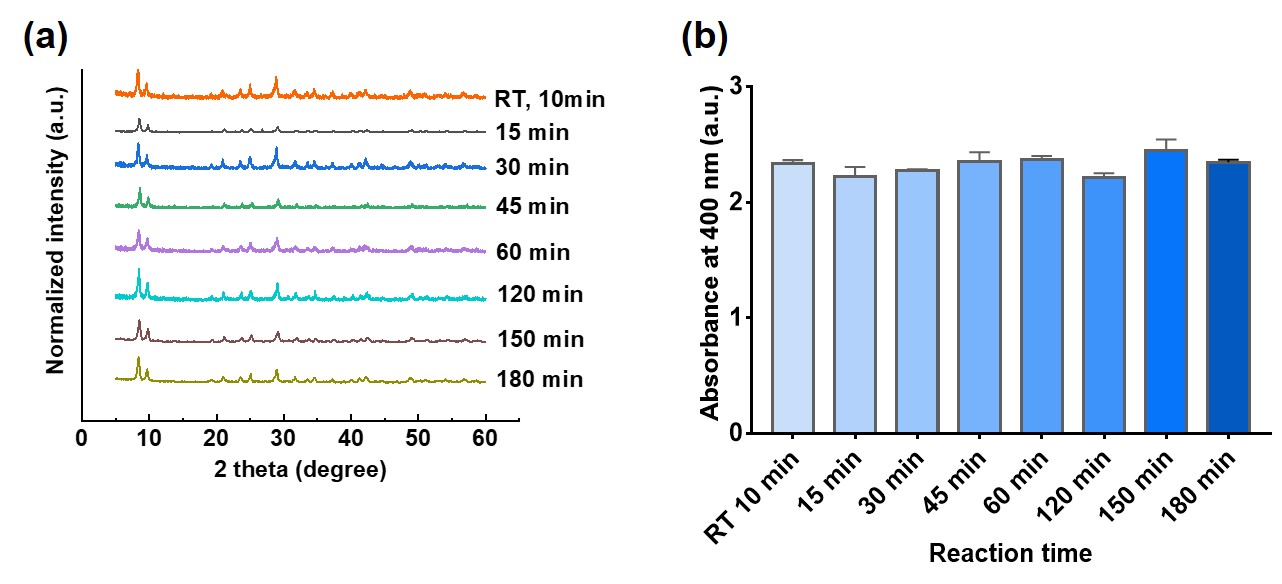


## **Supplementary Figure 5. Phase confirmation of Ce-FMAs synthesized at different time without modulator and comparison of their ALP-like activity with pNPP as the substrate.** (a) XRD patterns of Ce-FMAs synthesized at different time. (b) The absorbance at 400 nm of 4-nitrophenol for monitoring the catalytic hydrolysis of pNPP by Ce-FMAs synthesized at different time. Data are presented as mean ± standard error after removing the maximum and minimum with n=8.

## **Supplementary Figure 6. Ce-FMAs modulated by AA, FA and TFA with various ratios.** (a) XRD patterns of Ce-FMAs modulated by AA with different ratios. (b) XRD patterns of Ce-FMAs modulated by FA with different ratios. (c) XRD patterns for Ce-FMAs modulated by TFA with different ratios. (d) Maximum ratios of modulator to FMA to synthesize Ce-FMAs with different modulators.


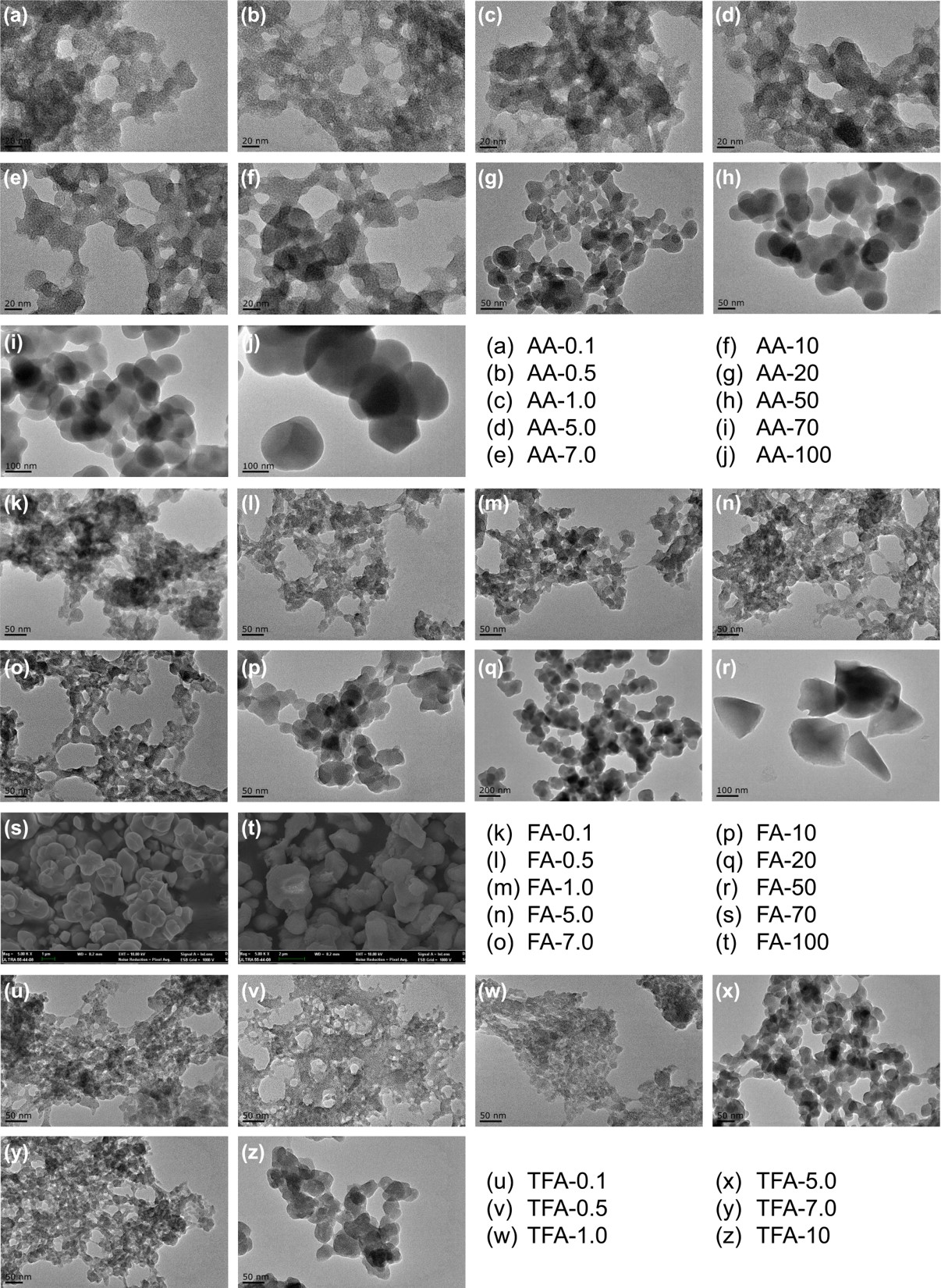


## Supplementary Figure 7. TEM images of Ce-FMAs at different magnification synthesized under various conditions. (a)-(j) Ce-FMAs synthesized with AA. (k)-(t) Ce-FMAs synthesized with FA. (u)-(z) Ce-FMAs synthesized with TFA.

## Supplementary Figure 8. N_2_ isotherms at 77 K of Ce-FMAs synthesized under various conditions. (a)-(j) Ce-FMAs synthesized with AA. (k)-(t) Ce-FMAs synthesized with FA. (u)-(z) Ce-FMAs synthesized with TFA.


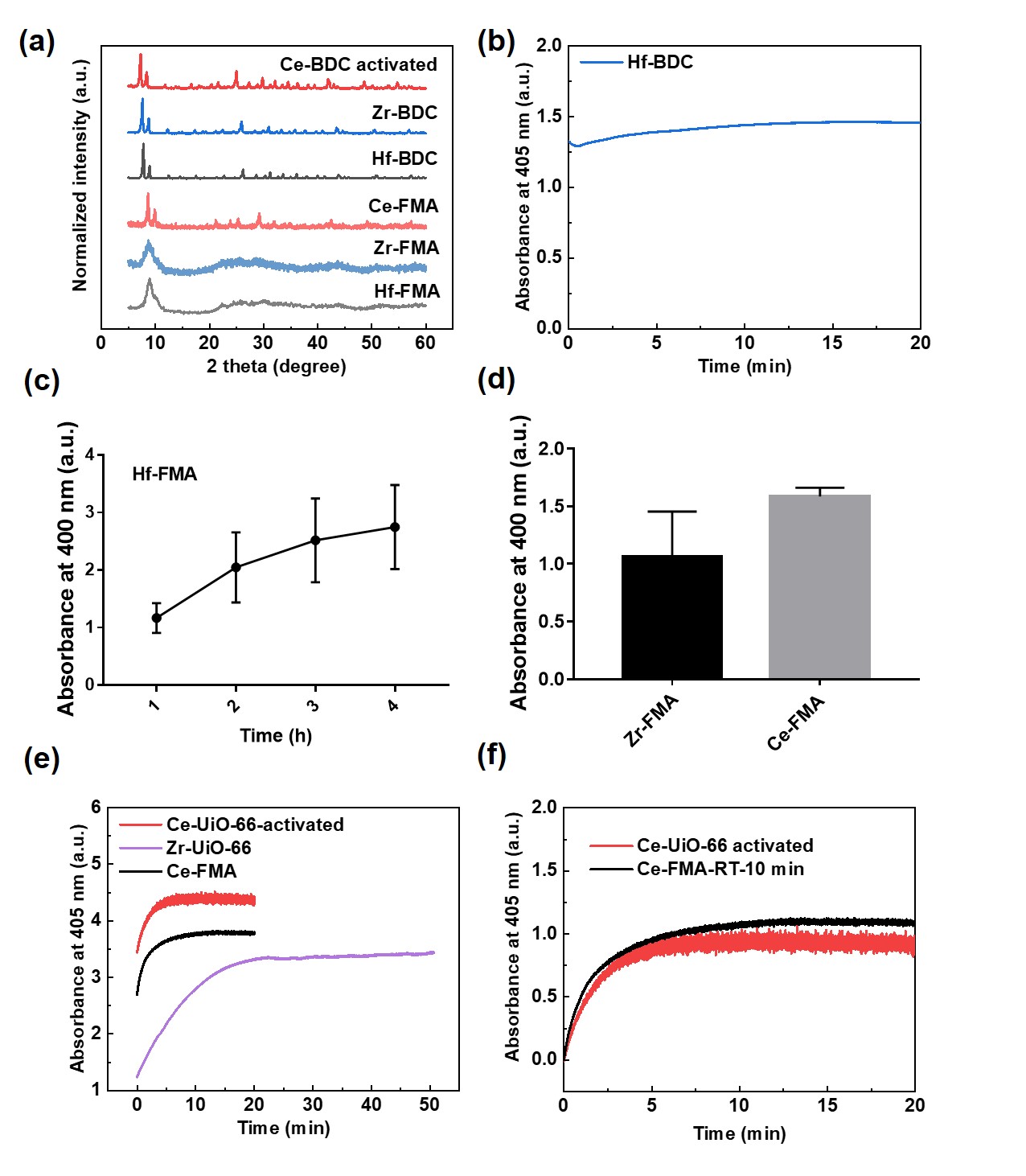


## **Supplementary Figure 9. Phase confirmation of UiO-66 structured MOFs linked by BDC or FMA, and comparison of ALP-like activity with pNPP as the substrate**. (a) XRD patterns for BDC and FMA linked UiO-66 structured MOFs. (b) Time dependent absorption at 405 nm for monitoring the hydrolysis of pNPP with Hf-BDC. (c) Time dependent absorption at 400 nm for monitoring the hydrolysis of pNPP with Hf-FMA. Data are presented as mean ± standard error of the mean (n=3). (d) Absorption at 400 nm for Zr-FMA and Ce-FMA systems after reacting with pNPP for 20 min at 37 °C. Data are presented as mean ± standard error of the mean (n=5 for Zr-FMA and n=3 for Ce-FMA). (e) Time dependent absorption at 405 nm for monitoring the hydrolysis of pNPP with Zr-BDC, Ce-BDC-activated and Ce-FMA. (f) Time dependent absorption at 405 nm for monitoring the hydrolysis of pNPP with Ce-FMA and Ce-BDC at the initial 20 min in Supplementary Figure 9e. Note, the background absorbance at 0 min was corrected at the same onset.





## Supplementary Figure 10. SEM/TEM images of six MOFs indicated by data. SEM images of Ce-BDC (a), Zr-BDC (c), Hf-BDC (d), Zr-FMA (e) and Hf-FMA (f). TEM image of Ce-FMA-FA-20-RT (b).





## **Supplementary Figure 11. Phosphatase-like activity of Ce-FMAs towards pNPP and BNPP.** (a) Schematic of the hydrolytic mechanism and the detection principles of pNPP and BNPP. Both pNPP and BNPP produce yellowish 4-nitrophenol after breaking phosphomonoester and phosphodiester bonds. (b) and (c) 3D histogram maps of the mass and specific ALP-like activity of Ce-FMAs with the substrate pNPP. (d) and (e) 3D histogram maps of the mass and specific phosphodiesterase-like activity of Ce-FMAs with the substrate BNPP. The activity is indicated by the colour gradient. Data are processed by removing the self-hydrolysis effect and the initial background of nanozymes. Data are presented as the mean ± standard error, n=8.


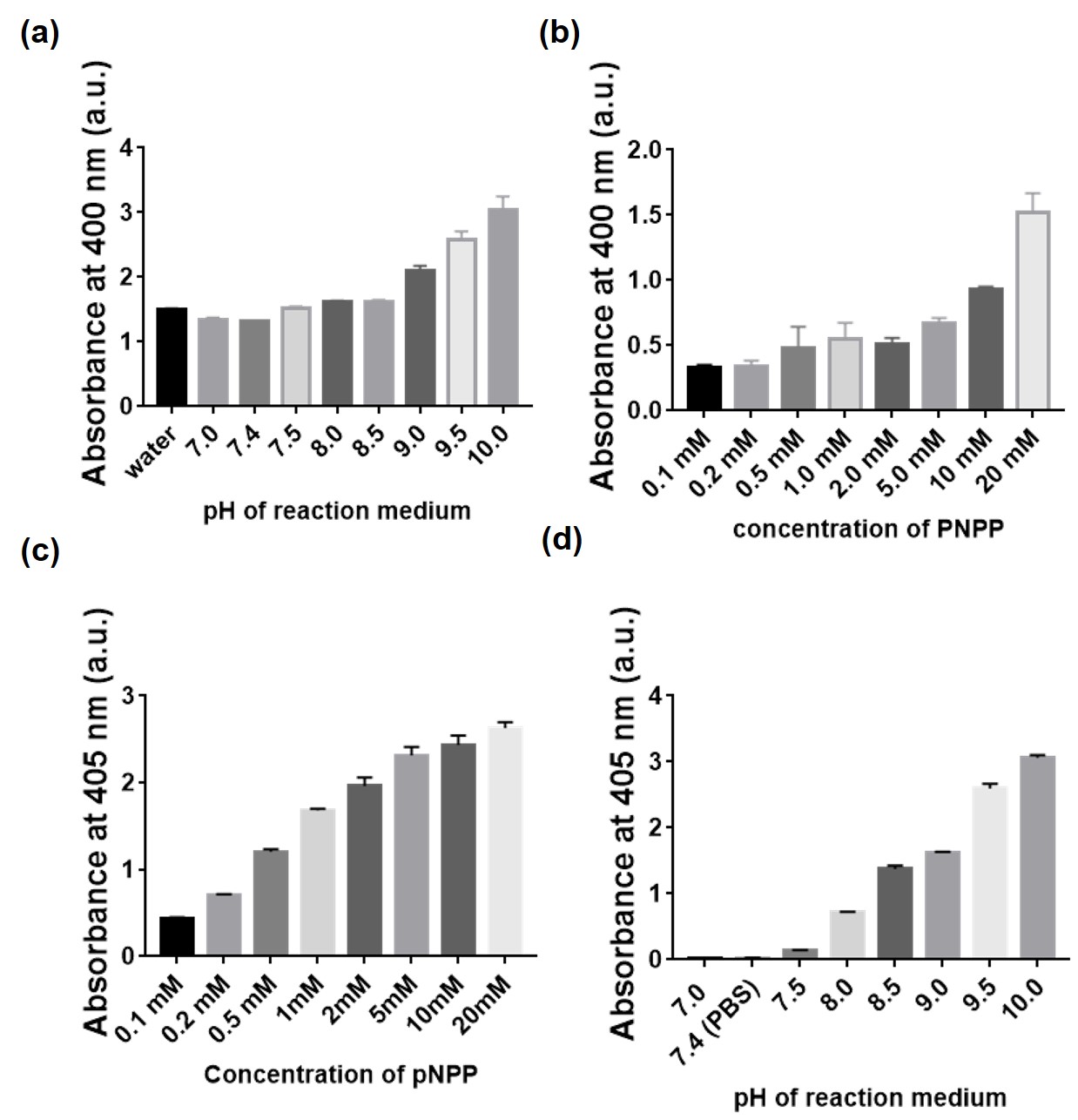


## Supplementary Figure 12. Optimization pH of reaction medium and concentration of pNPP. (a) The absorbance at 400 nm of 4-nitrophenol for monitoring the catalytic hydrolysis of pNPP by Ce-FMA-FA-20-RT at different pH for 30 min. Data are presented as mean ± standard error of the mean (n=3). (b) The absorbance at 400 nm of 4-nitrophenol for monitoring the catalytic hydrolysis of pNPP at pH 10.0 by Ce-FMA-FA-20-RT with different concentrations of pNPP for 10 min. Data are presented as mean ± standard error of the mean (n=6). (c) The absorbance at 405 nm of 4-nitrophenol for monitoring the catalytic hydrolysis of pNPP by ALP (1 U) at different pH for 20 min. Data are presented as mean ± standard error of the mean (n=3). (d) The absorbance at 405 nm of 4-nitrophenol for monitoring the catalytic hydrolysis of pNPP at pH 10.0 by ALP (1 U/mL) with different concentrations of pNPP for 10 min. Data are presented as mean ± standard error of the mean (n=3).


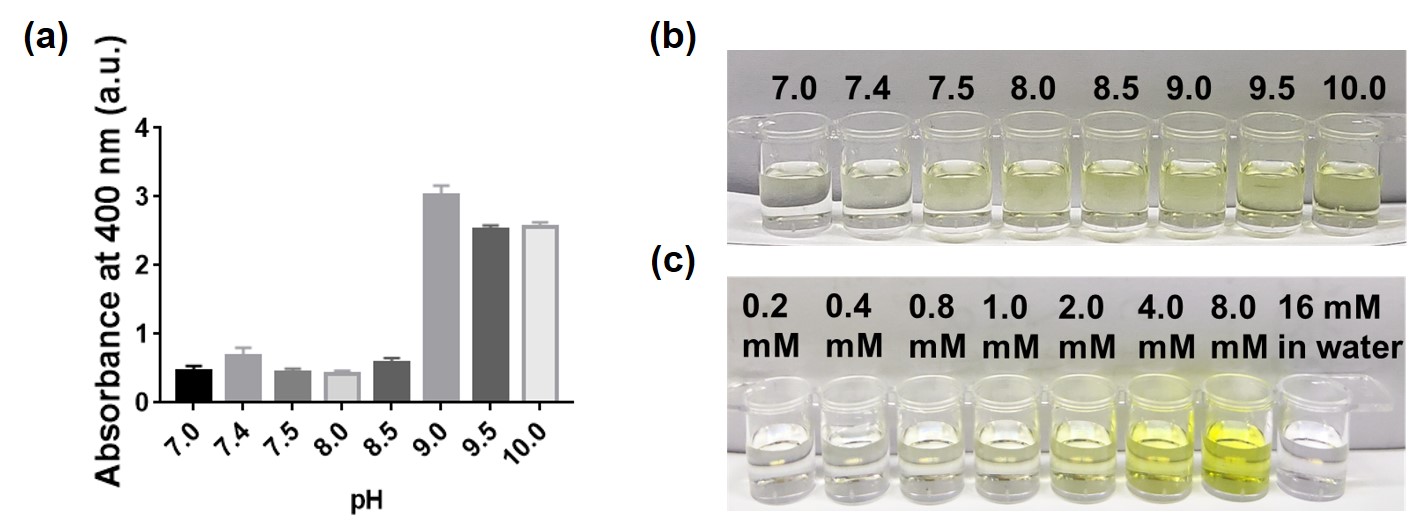


## Supplementary Figure 13. pH dependent hydrolytic activity of Ce-FMA-FA-20-RT towards BNPP and the spontaneous hydrolysis of BNPP under different pH or concentrations. (a) The absorbance at 400 nm of 4-nitrophenol for monitoring the catalytic hydrolysis of BNPP. Data are presented as mean ± standard error of the mean (n=4). (b) Photograph of BNPP with a concentration of 2 mM under different pH. (c) Photograph of BNPP with varied concentrations under pH 9.0 or water.


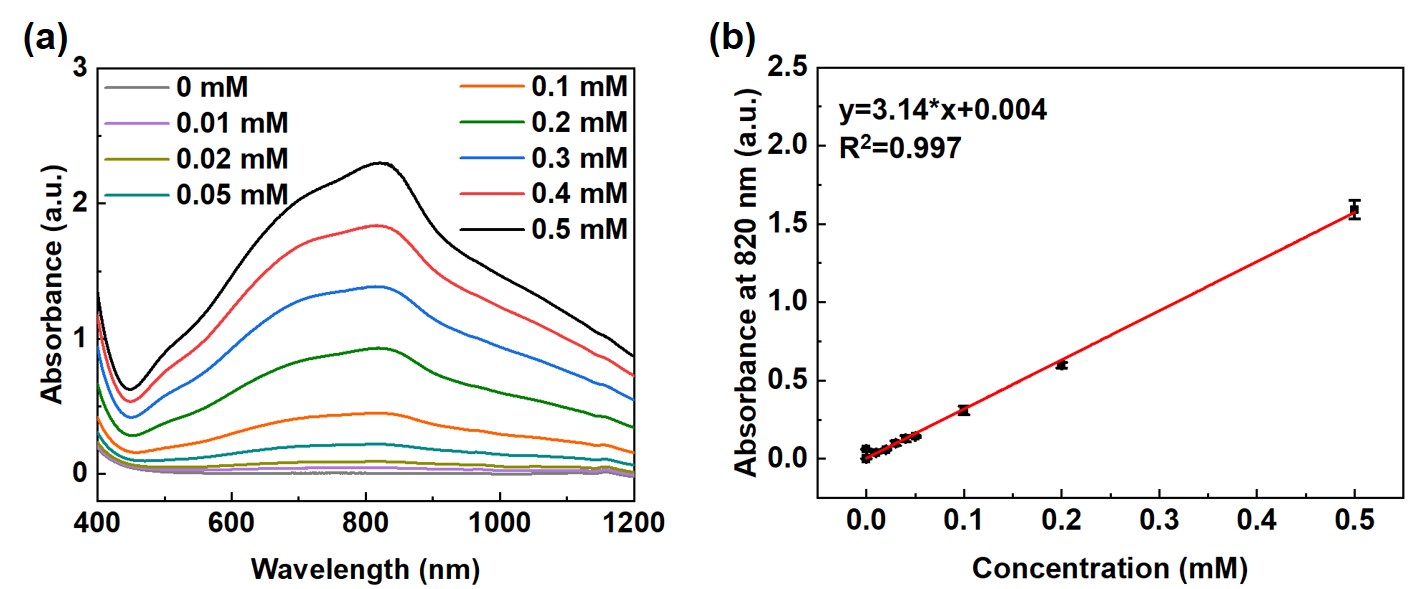


## Supplementary Figure 14. Molybdenum-blue colorimetric approach for detection of free phosphates. (a) The absorbance spectra with varied concentration of phosphate samples. (b) The linear fitting curve of absorbance at 820 nm versus phosphate concentration. Data are presented as mean ± standard error of the mean (n=4) and the experiments were conducted as previously reported^10^.


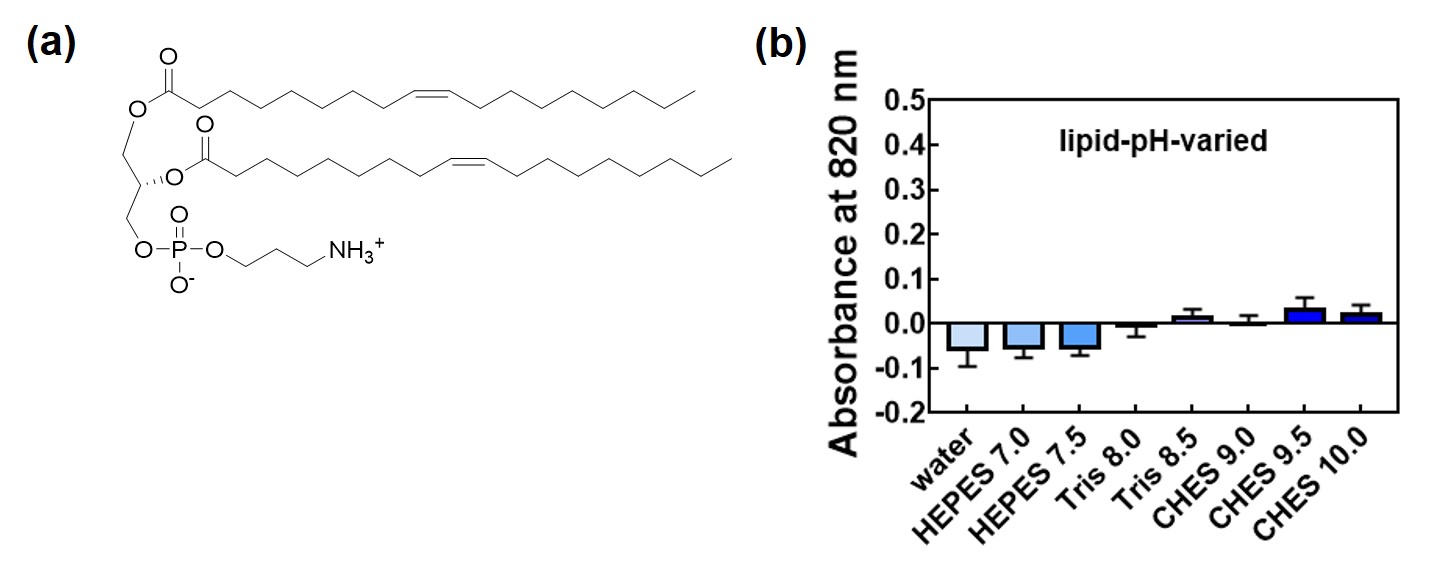


## Supplementary Figure 15. Phosphatase-like activity of Ce-FMA-FA-20-RT towards cephalin. (a) Chemical structure of cephalin. (b) pH dependent cleaving effect of Ce-FMA-FA-20-RT towards cephalin. Data are processed by removing the blank groups (without catalyst), and presented as the mean ± standard error, n=4.

## Supplementary Figure 16. Horizontal electrophoresis of DNA treated by Ce-FMA-FA-20-RT. Lanes 1 and 2: DNA treated by Ce-FMA-FA-20-RT after 24 and 12 h, respectively; lane 3: DNA with PBS only; lane 4: DNA markers with 0.5, 1, 2, 3, 4, 6, 8, and 10 kbp in order.


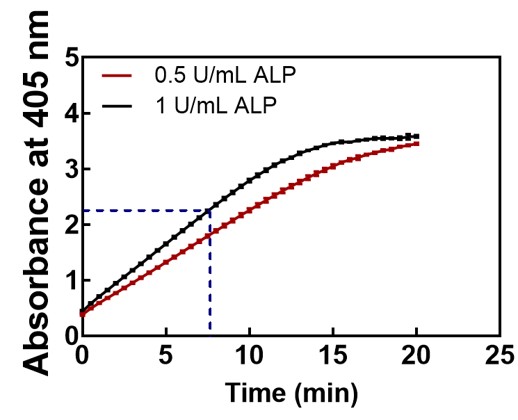


## Supplementary Figure 17. Absorbance at 405 nm of 4-nitrophenol for monitoring the catalytic hydrolysis of 10 mM pNPP by different concentrations of ALP in pH 10.0 at 37 ºC. Data are presented as mean ± standard error of the mean (n=3). The dashed line is used to determine the half-time of ALP (1U).


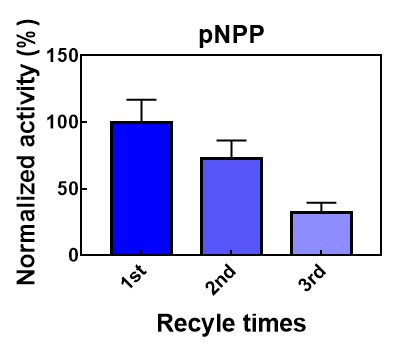


## Supplementary Figure 18. The recyclability of Ce-FMA-FA-20-RT towards pNPP. Experiments were conducted with 10 mM pNPP in the presence of 0.5 mg/mL Ce-FMA-FA-20-RT in pH 10.0 at 37 ºC.

**
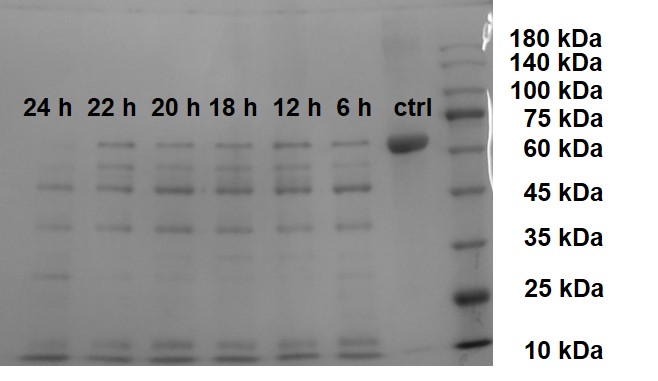
**

## Supplementary Figure 19. Coomassie blue stained SDS-PAGE of 2 mg/mL BSA hydrolyzed by 1 mg/mL trypsin at 37 °C in 1×PBS buffer. Samples were collected at different time.

## Supplementary Figure 20. Characterization of MOF-808 and its protease-like activity towards BSA at 60 °C in PBS. (a) XRD pattern of MOF-808. (b) N_2_ isotherms at 77 K of MOF-808. (c) GPC profiles of BSA before and after MOF-808 treated for 24 h. (d) Comparison of conversion rate and BET surface area normalized conversion rate between MOF-808 and Ce-FMA-FA-20-RT.


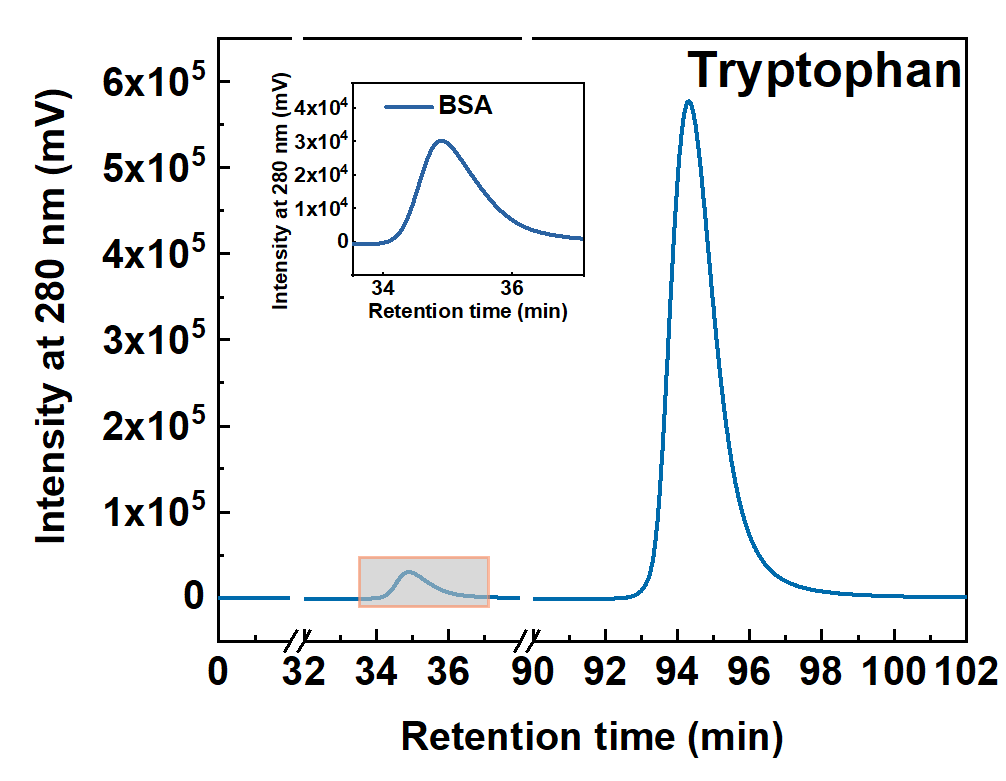


## Supplementary Figure 21. GPC profiles of BSA and tryptophan.

**
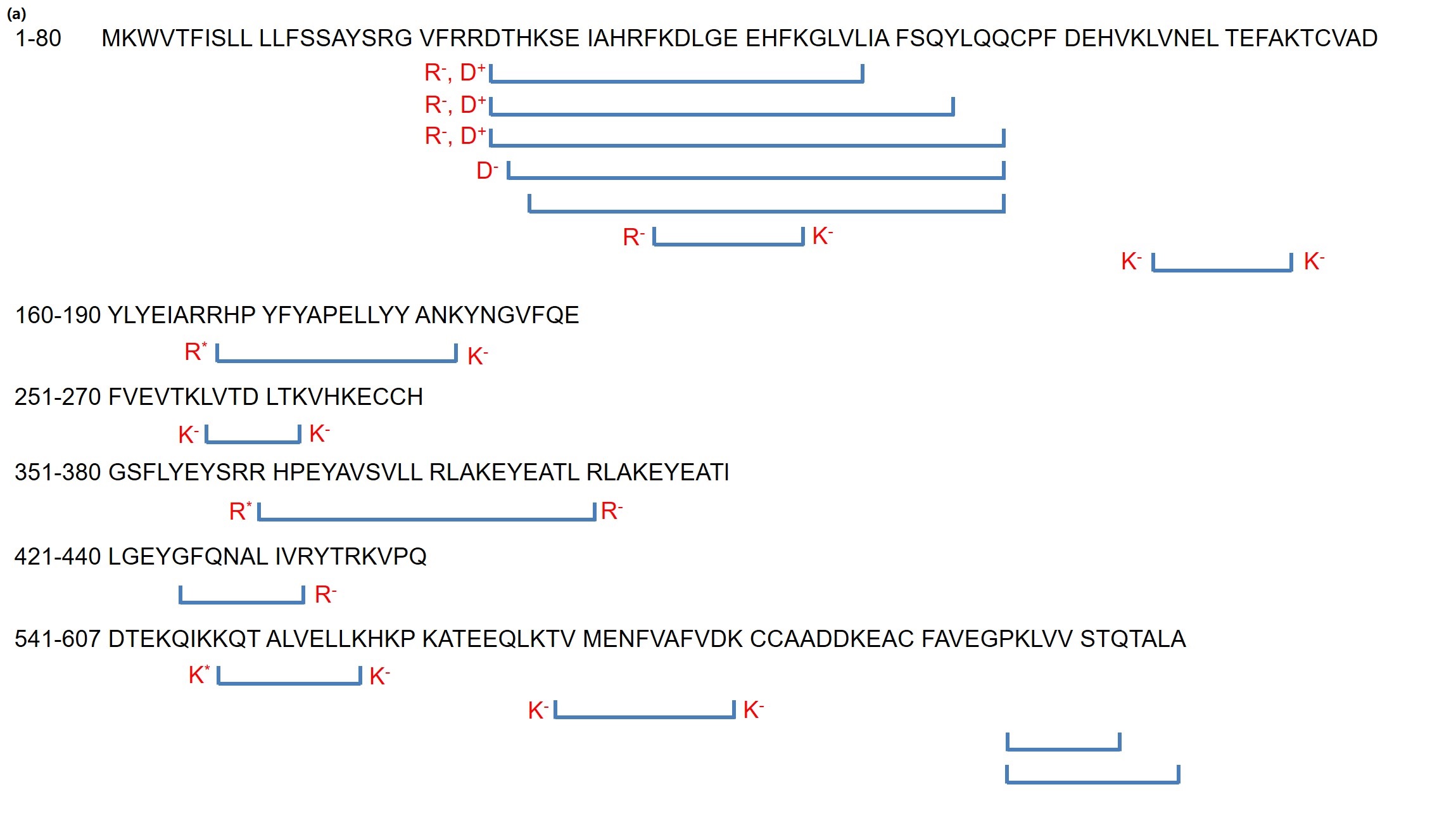
**

**
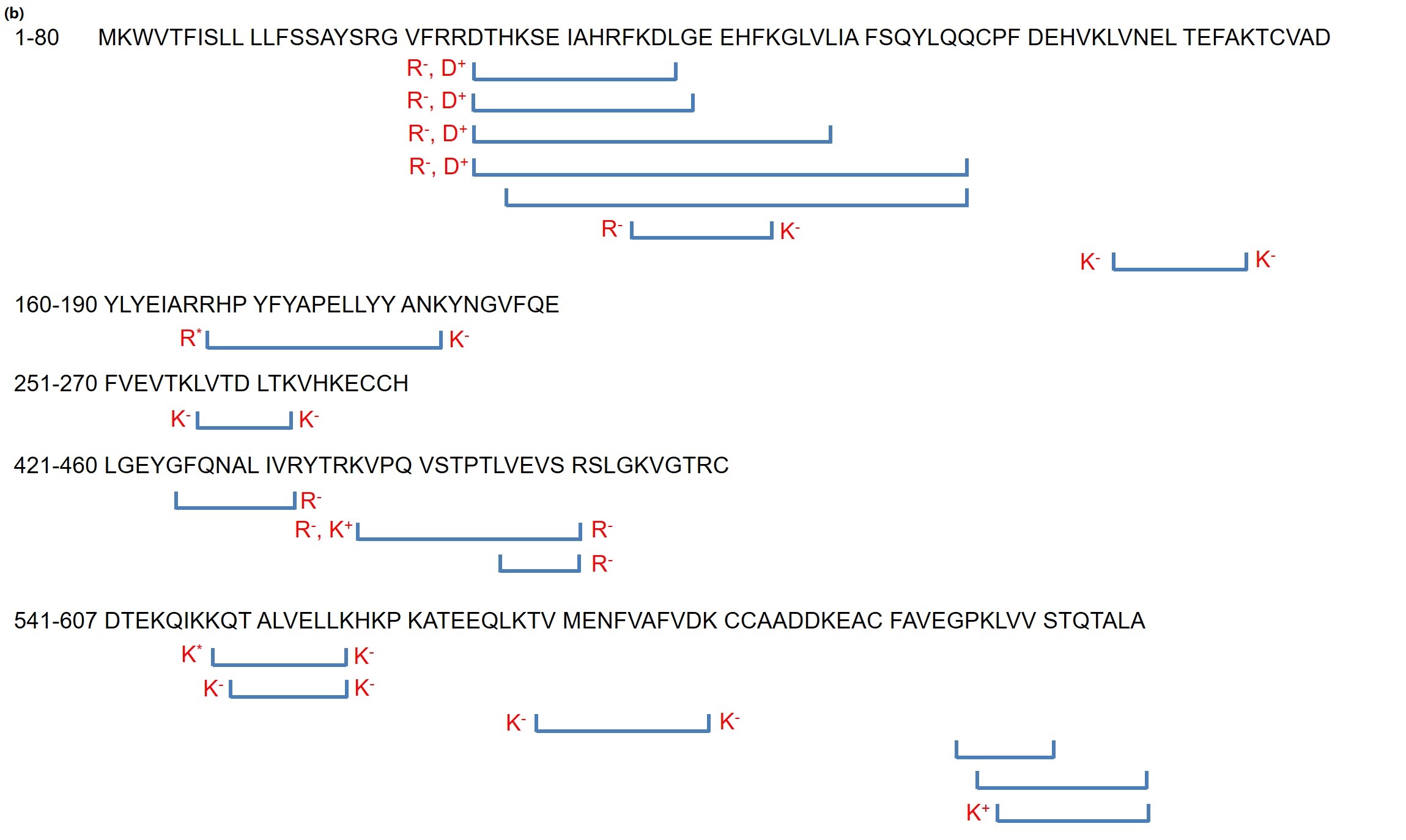
**

**
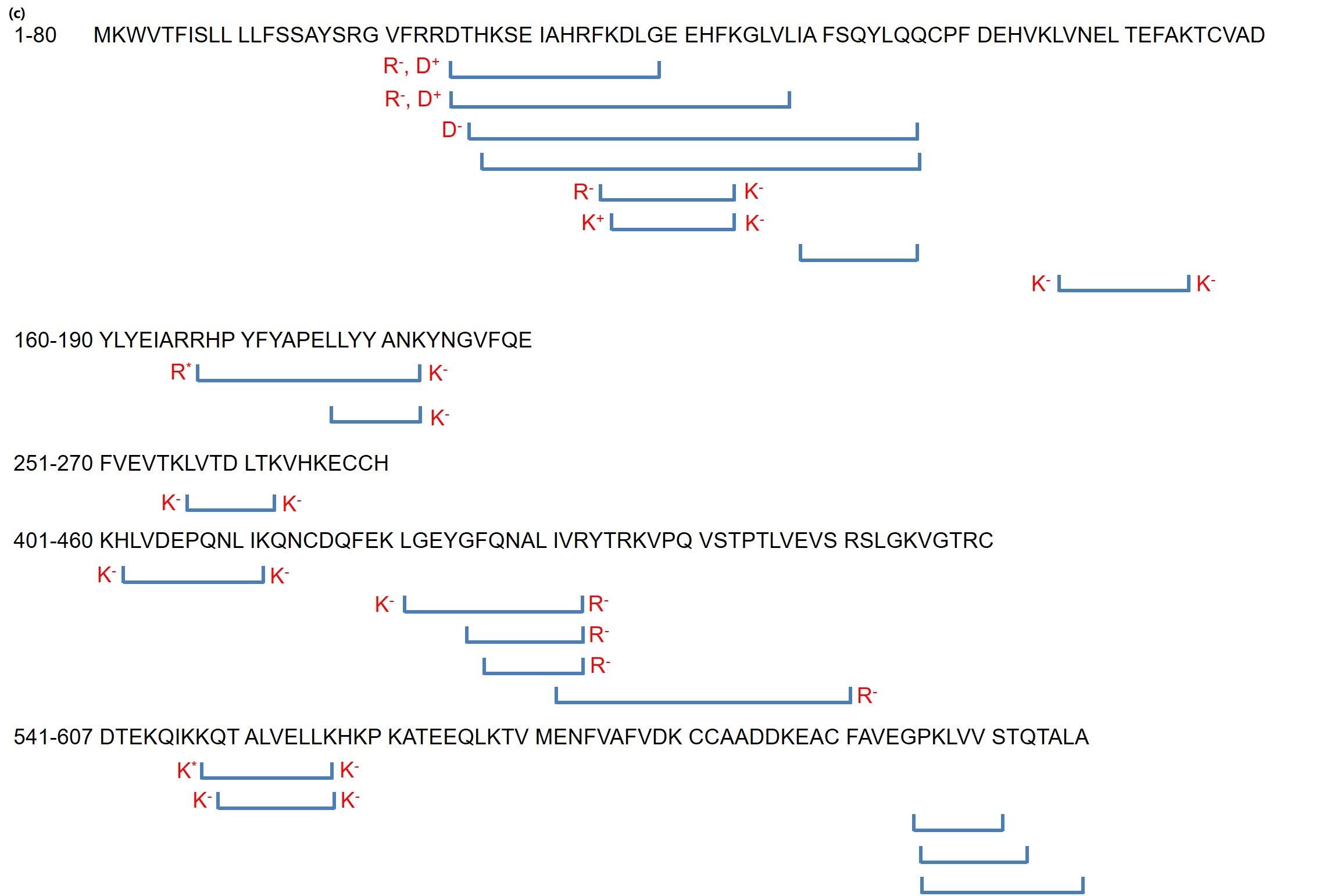
**

**
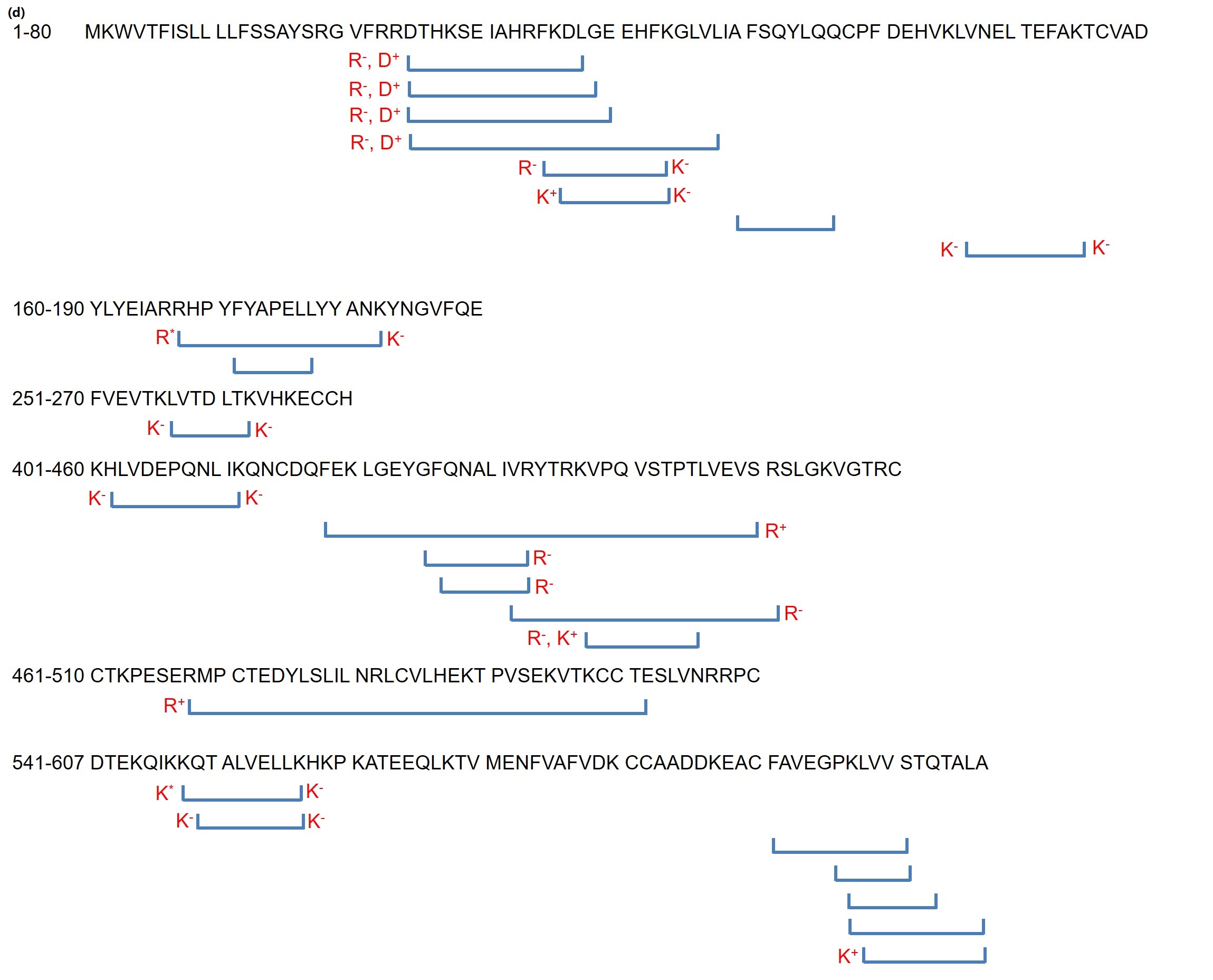

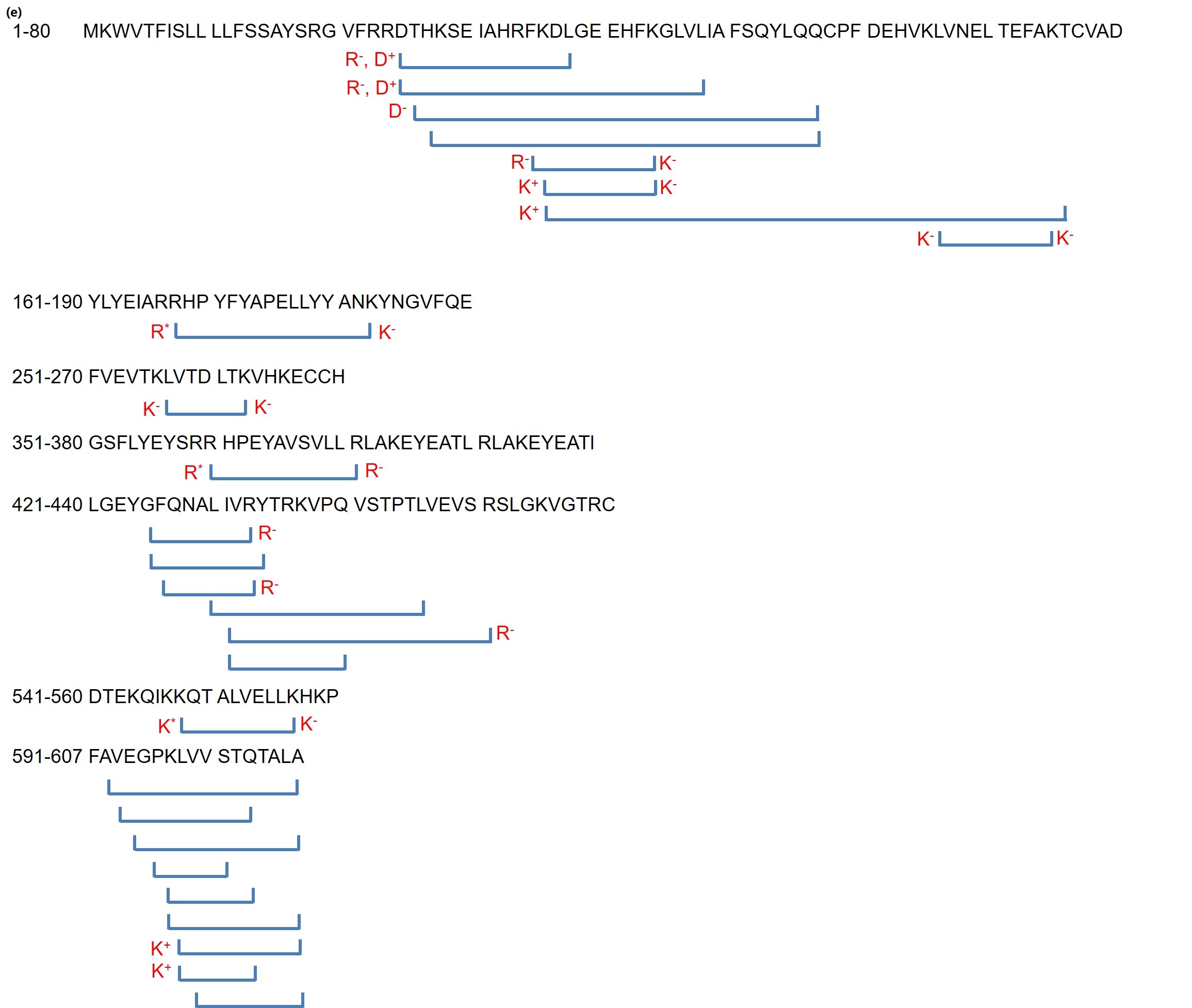
**

**
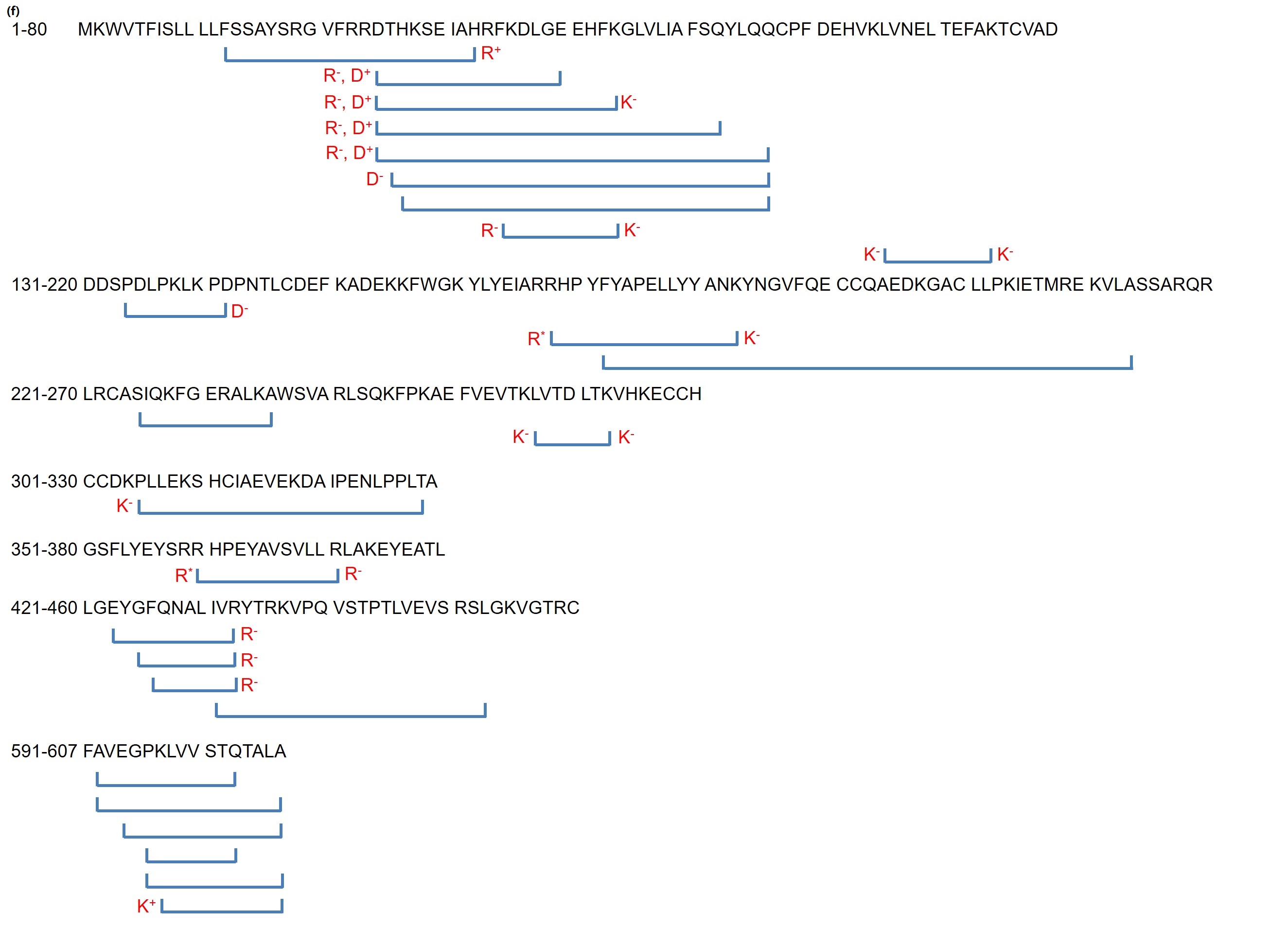
**

**
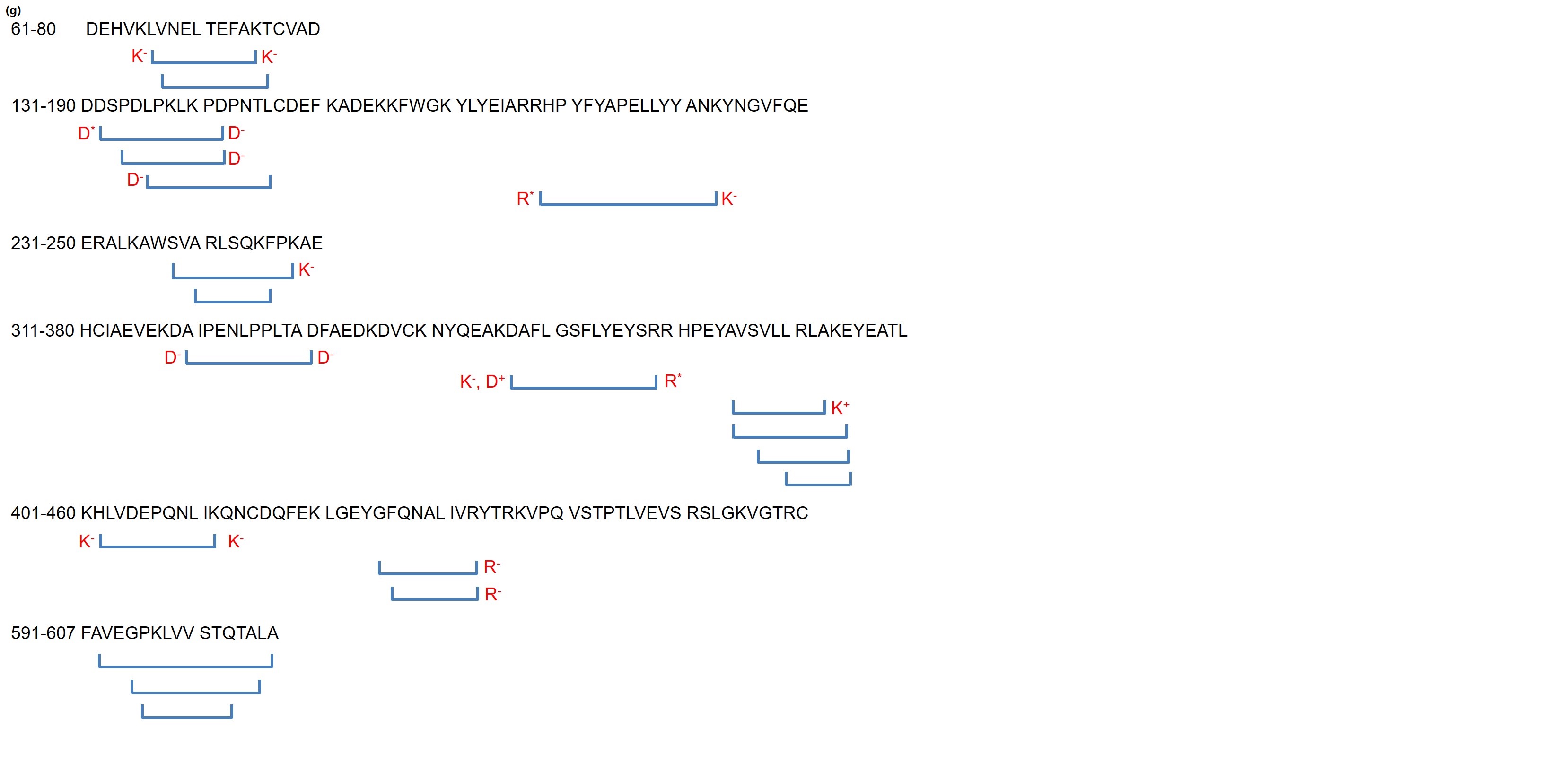
**

## Supplementary Figure 22. Sequence coverage of BSA and Ce-FMA-FA-20-RT cleaved peptides. (a) 0.5 h at 60 °C. (b) 1 h at 60 °C. (c) 1.5 h at 60 °C. (d) 2.0 h at 60 °C. (e) 6.0 h at 60 °C. (f) 12 h at 60 °C. (g) 36 h at 60 °C.


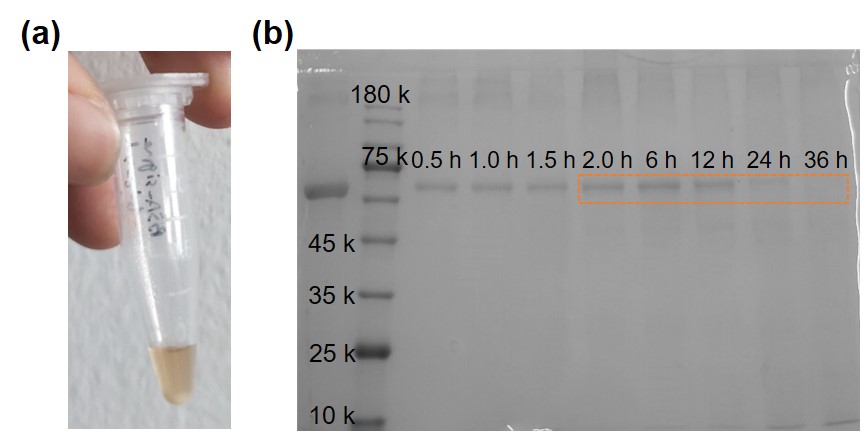


## Supplementary Figure 23. The adsorption behavior of protein onto Ce-FMA-FA-20-RT. (a) Ce-FMA-FA-20-RT could be decomposed by EDTA-Tris-HCl (8.0). (b) SDS-PAGE of the mixture samples collected at various time.


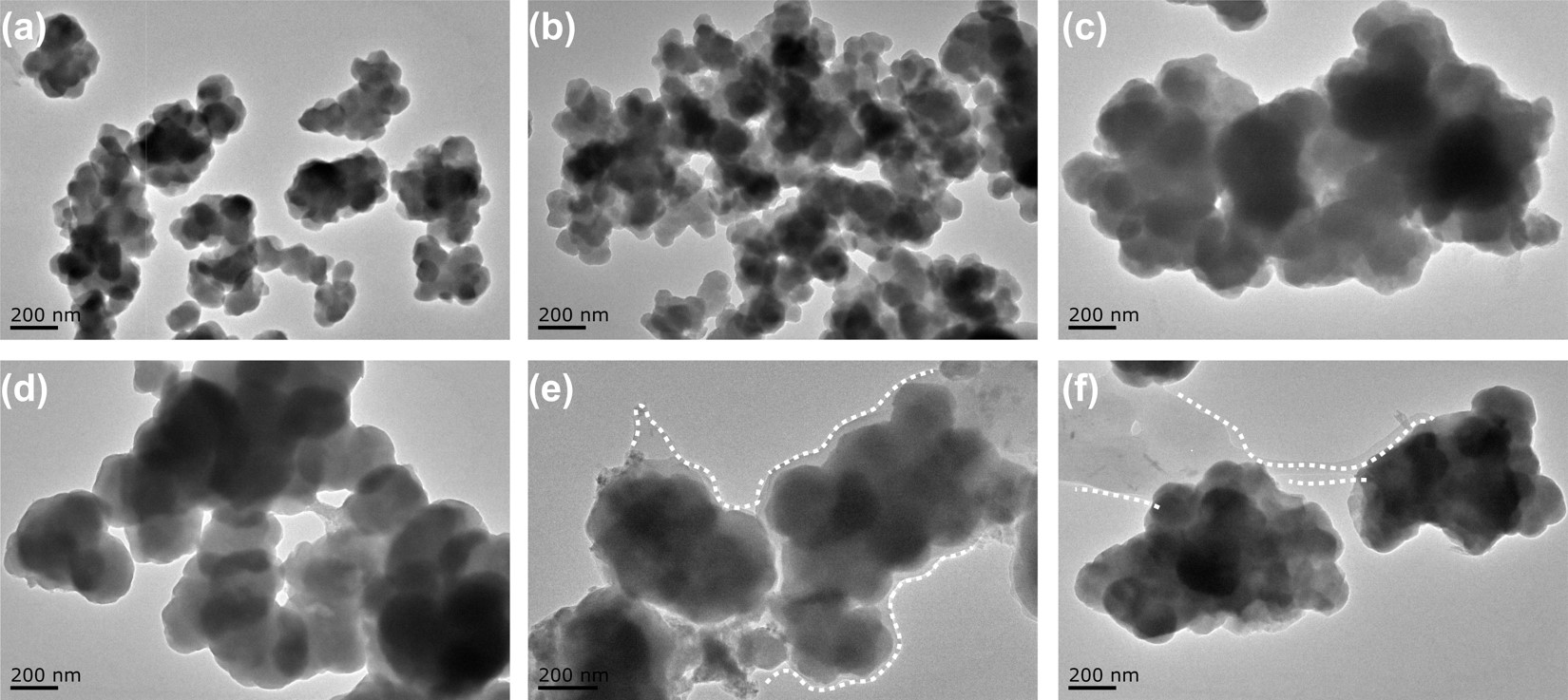


## Supplementary Figure 24. TEM images of Ce-FMA-FA-20-RT before and after reaction with various substrates. (a) TEM image of Ce-FMA-FA-20-RT prior to reactions. (b) TEM image of Ce-FMA-FA-20-RT after reacted with carboxymethyl chitosan at pH 8. (c) TEM image of Ce-FMA-FA-20-RT after reacted with pNPP. (d) TEM image of Ce-FMA-FA-20-RT after reacted with BNPP. (e) TEM image of Ce-FMA-FA-20-RT after reacted with BSA at 60 °C. (f) TEM image of Ce-FMA-FA-20-RT after reacted with BSA at 37 °C. The white dashes in (e) and (f) indicate the adsorbed protein/peptide when hydrolyzing due to electrostatic interaction.


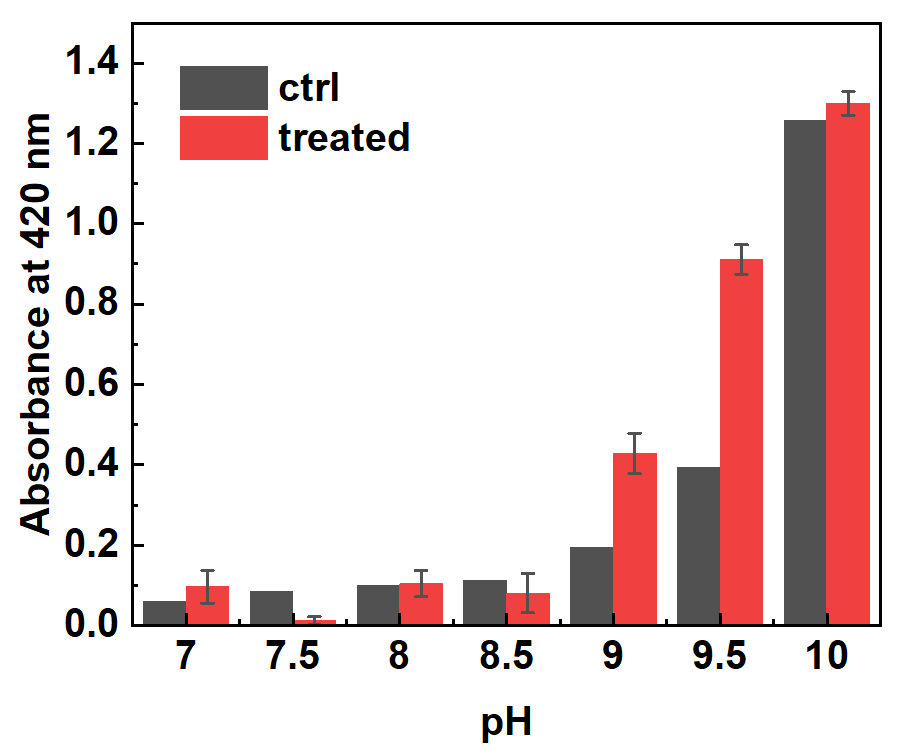


## Supplementary Figure 25. pH dependent cleavage ability towards 2-nitrophenyl β-D-galactopyranoside. The absorption at 420 nm of 2-nitrophenol for monitoring the catalytic hydrolysis of 2-nitrophenyl β-D-galactopyranoside by Ce-FMA-FA-20-RT at different pH for 8 h at 60 °C. Data are presented as mean ± standard error of the mean (n=3).

## Supplementary Figure 26. Ion chromatography curves of disaccharides with α/β-glycosidic bonds and their monosaccharides. (a) and (b) The isolated standard curves of monosaccharide and the enlarged discrimination of galactose and glucose. (c) Curves of maltose with/without Ce-FMA-FA-20-RT in pH 4 or pH 8 solutions. (d) Curves of lactose with/without Ce-FMA-FA-20-RT in pH 4 or pH 8 solutions.


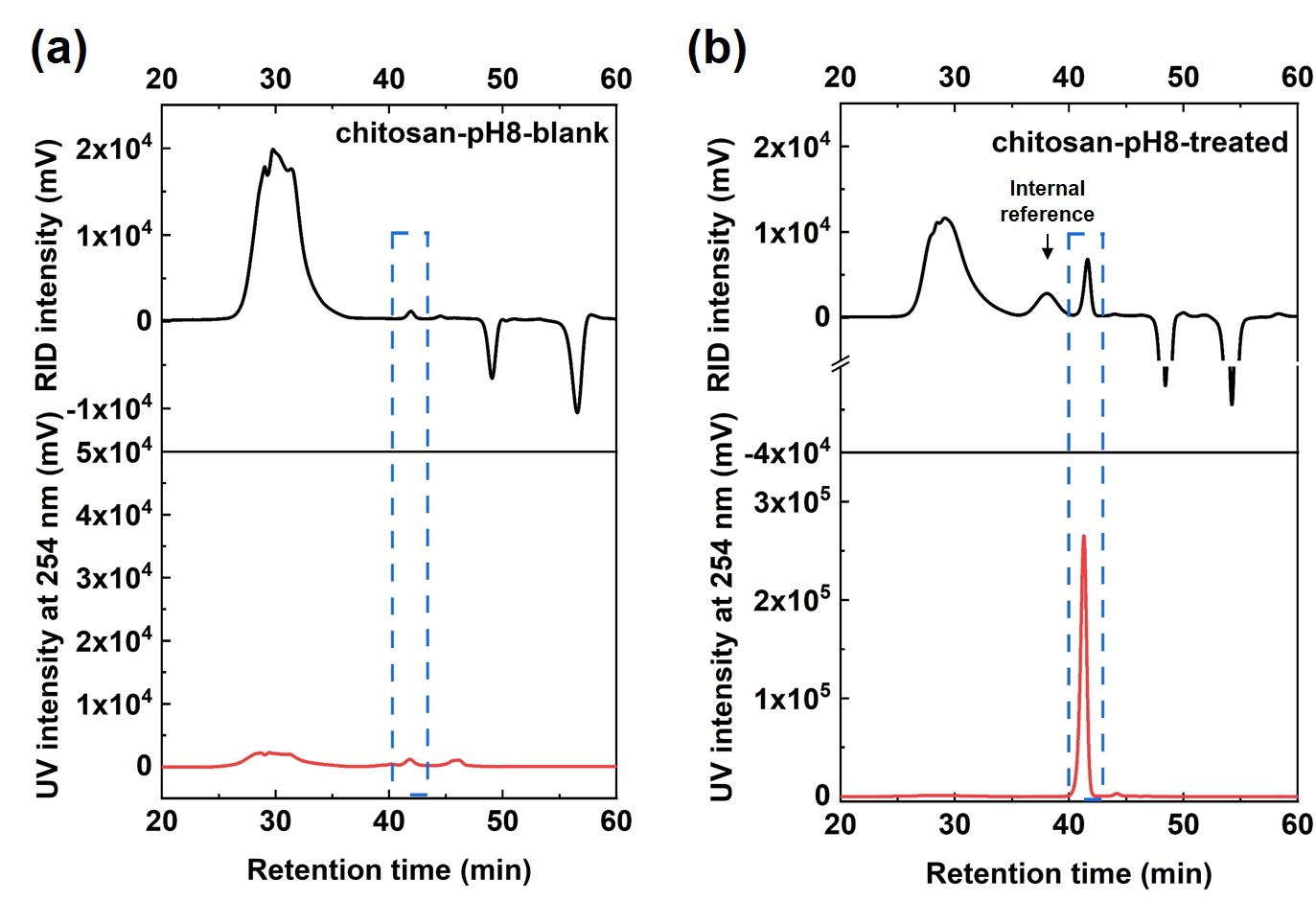


## Supplementary Figure 27. GPC profiles (detected by RID and UV) of carboxymethyl chitosan before (a) and after treatment (b) with Ce-FMA-FA-20-RT at 60 °C under pH 8.0 for 26.5 h. The dashed blue rectangle outlines the cleaved product by Ce-FMA-FA-20-RT.


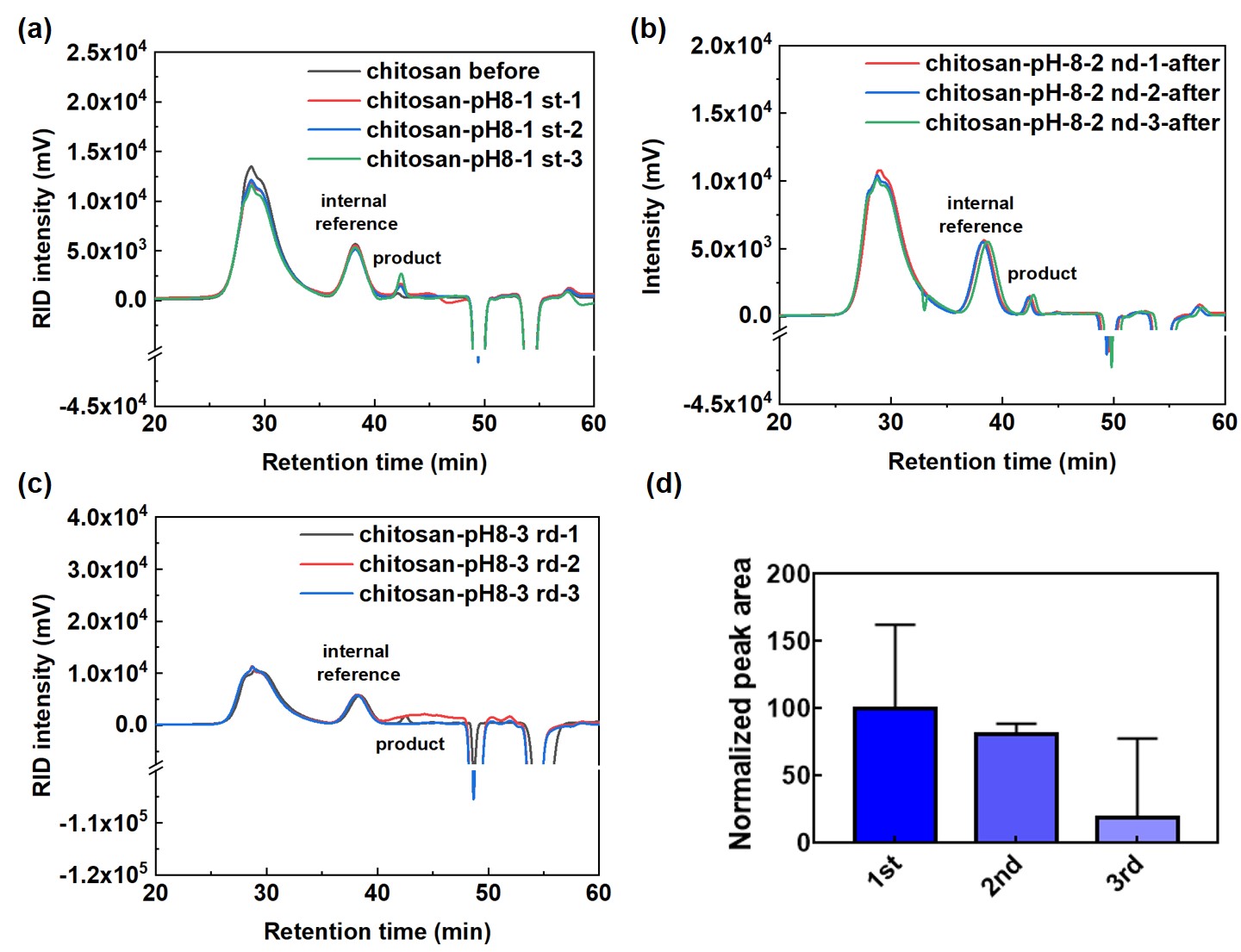


## Supplementary Figure 28. Reusability of carboxymethyl chitosan by Ce-FMA-FA-20-RT. GPC profiles (detected by RID) of carboxymethyl chitosan with the treatment of Ce-FMA-FA-20-RT at 37 °C under pH 8.0 for the first time (a), second time (b) and third time (c). (d) Normalized peak area (detected by RID) of carboxymethyl chitosan with the treatment of Ce-FMA-FA-20-RT at 37 °C under pH 8.0 for three times recycle. Data are processed by removing the blank groups (without catalyst) and presented as mean ± standard error of the mean (n=3).


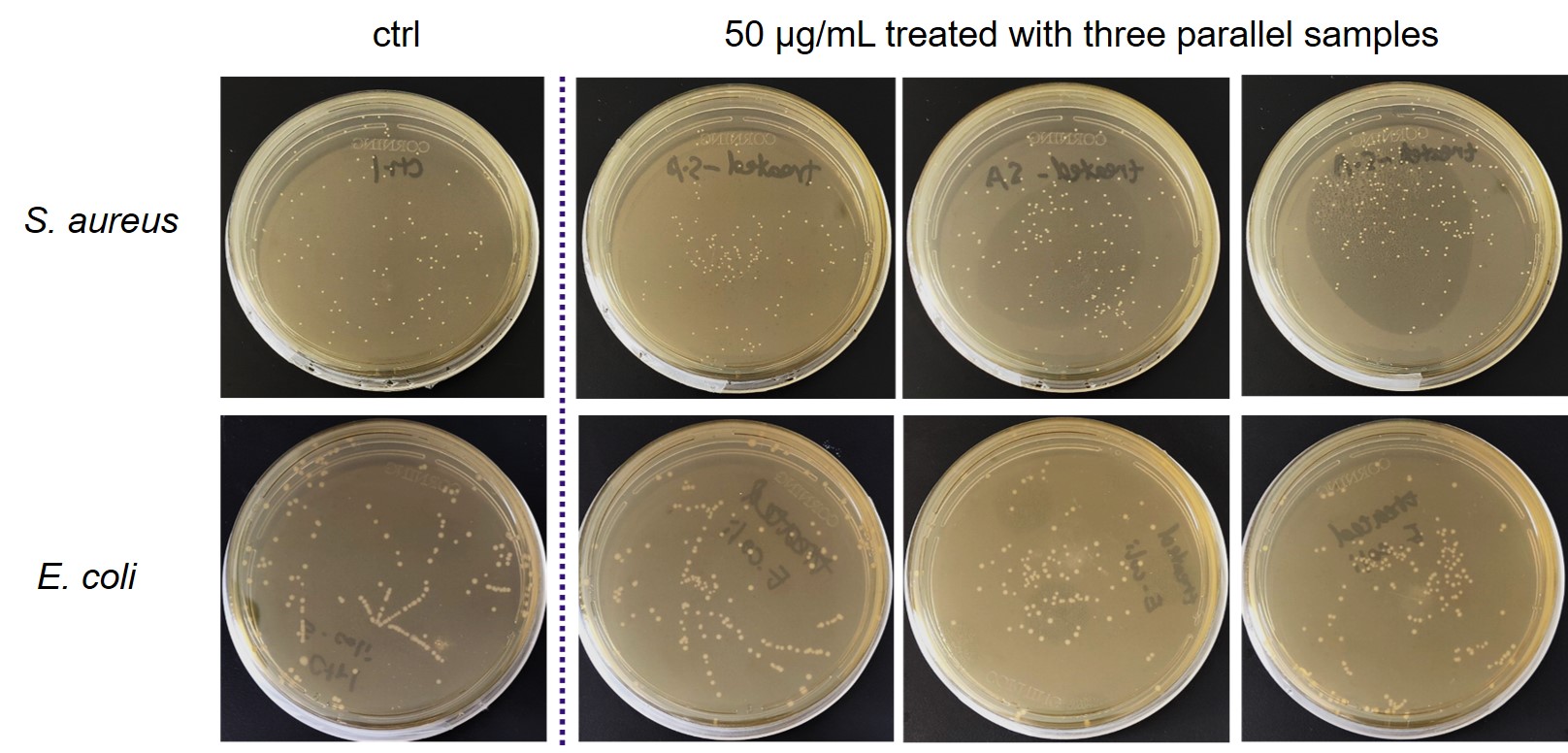


## Supplementary Figure 29. Bacteria spread on agar plates treated with/without Ce-FMA-FA-20-RT.

# Supplementary Tables

## Supplementary Table 1. Classification and schematic reaction of natural hydrolases.

| **Schematic diagram of hydrolytic reaction** | **Hydrolase** |
| --- | --- |
|  | esterase  lipase |
|  | phosphatase  nuclease  phosphodiesterase |
|  | protease  peptidase  amidase |
|  | glycosidase |

## Supplementary Table 2. Absolute hardness of selected metal ions^11-13^.

| **Element** | **Oxidation state** | **Ionization energy** (eV) | **Electron affinity**  (eV) | **Absolute hardness**  η |
| --- | --- | --- | --- | --- |
| Copper | II | 36.8 | 20.3 | 8.3 |
| Zinc | II | 39.7 | 18.0 | 10.8 |
| Chromium | III | 49.2 | 31.0 | 9.1 |
| Titanium | IV | 20.4 | 6.11 | 7.2 |
| Cerium | IV | 65.6 | 36.8 | 14.4 |
| Hafnium | IV | 68.4 | 33.4 | 17.5 |
| Zirconium | IV | 98.5 | 80.3 | 9.1 |

## Supplementary Table 3. Hydrolytic effect of Ce-FMA-FA-20-RT towards broad substrate scope of different hydrolases.

| **Hydrolytic substrate** | | **Effect** | **Hydrolase mimicked** | |
| --- | --- | --- | --- | --- |
| pNPP (4-nitrophenyl phosphate disodium salt hexahydrate) |  | positive | phosphomonoesterase  (alkaline phosphatase) | |
| BNPP (bis(4-nitrophenyl) phosphate) |  | positive | phosphodiesterase | |
| AMP | 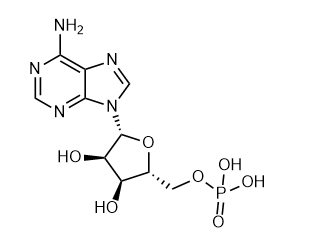 | positive | phosphomonoesterase | |
| ADP | 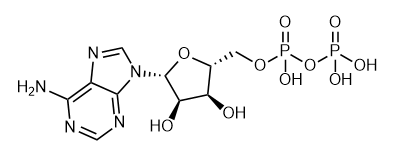 | positive | phosphodiesterase | |
| ATP | 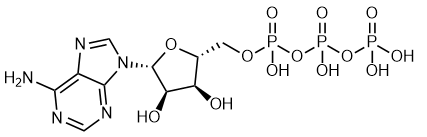 | positive | phosphotriesterase | |
| β-GP | 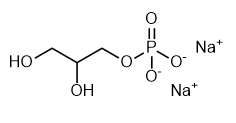 | positive | phosphomonoesterase | |
| cephalin | 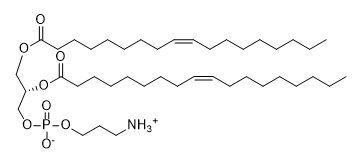 | negligible | phosphomonoesterase | |
| DNA |  | negligible | nuclease | |
| BSA | 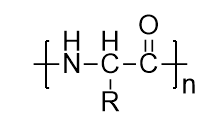 | positive | protease | |
| lactose |  | negligible | | β-galactosidase |
| maltose |  | negligible | | maltase |
| 2-nitrophenyl β-D-galactopyranoside |  | positive | | β-galactosidase |
| 4-nitrophenyl N-acetyl-β-D-glucosaminide |  | positive | | β-N-acetylglucosaminidase |
| carboxymethyl chitosan |  | positive | | chitosanase |
| pNA (p-nitrophenyl acetate) |  | negligible | | carbonic anhydrase |

## Supplementary Table 4. Summary of modulator used in hydrolase-like MOFs.

| **MOF** | **Modulator** | **Ref.** |
| --- | --- | --- |
| DUT-84 | AA | 2 |
| MIL-101(Cr) | none | 3 |
| H_5_PV_2_Mo_10_O_40_@ MIL-101(Cr) | AA | 14 |
| Cu_2_(C_9_H_3_O_6_)_4/3_ MOF(HKUST-1) | none | 15 |
| CuBTC (HKUST-1) | none | 4 |
| MOF(Cu-BTC)/g-C_3_N_4_ nanospheres | none | 16 |
| MOF-808 | AA | 2 |
|  | FA | 7, 5 |
| NU-1000 | benzoic acid | 5,7,17,18 |
|  | TFA | 6 |
| PCN-222/MOF-545 | benzoic acid | 6 |
|  | benzoic acid and TFA | 19 |
| PCN-777 | TFA | 7 |
| Spirof-MOF | FA | 8 |
| UiO-66 (Zr) | none | 20, 5 |
|  | AA | 21 |
|  | AA and HCl | 2 |
|  | HCl | 22,23 |
| UiO-66 (Ce) | none | 24 |
| UiO-66-0.25NH_2_ | HCl and TFA | 20 |
| UiO-66-0.50NH_2_ | HCl and TFA | 20 |
| UiO-66-0.75NH_2_ | HCl and TFA | 20 |
| UiO-66-NH_2_ | none | 25 |
|  | HCl and TFA | 20 |
|  | HCl | 7,22 |
| UiO-66-NO_3_ | HCl | 22 |
| UiO-66-OH | HCl | 22 |
| UiO-67 | none | 5,20,26 |
|  | AA | 27 |
|  | HCl | 22,28 |
| UiO-67-0.25NH_2_ | HCl and TFA | 20 |
| UiO-67-0.50NH_2_ | HCl and TFA | 20 |
| UiO-67-0.75NH_2_ | HCl and TFA | 20 |
| UiO-67-NH_2_ | HCl | 22 |
|  | HCl and TFA | 20 |
| UiO-67-N(Me)_2_ | HCl | 22 |

## Supplementary Table 5. The amount of modulator added to synthesize Ce-FMA

| **Modulator** | **Ratio of modulator/ligand** | **Volume of modulator** | **Volume of water** | **Total volume** |
| --- | --- | --- | --- | --- |
| AA | 0.1 | 15.945 µL | 29.984 mL | 30 mL |
|  | 0.5 | 85.77 µL | 29.914 mL |  |
|  | 1.0 | 159.54 µL | 29.840 mL |  |
|  | 5.0 | 857.76 µL | 29.142 mL |  |
|  | 7.0 | 1.2 mL | 28.8 mL |  |
|  | 10 | 1.595 mL | 28.405 mL |  |
|  | 20 | 3.431 mL | 26.569 mL |  |
|  | 50 | 8.578 mL | 21.422 mL |  |
|  | 70 | 12 mL | 18 mL |  |
|  | 100 | 15.954 mL | 14.046 mL |  |
| FA | 0.1 | 11.32 µL | 29.99 mL |  |
|  | 0.5 | 56.59 µL | 29.94 mL |  |
|  | 1.0 | 113.2 µL | 29.887 mL |  |
|  | 5.0 | 565.9 µL | 29.434 mL |  |
|  | 7.0 | 792.3 µL | 29.207 mL |  |
|  | 10 | 1.132 mL | 28.868 mL |  |
|  | 20 | 2.264 mL | 27.736 mL |  |
|  | 50 | 5.659 mL | 24.341 mL |  |
|  | 70 | 7.923 mL | 22.077 mL |  |
|  | 100 | 11.32 mL | 18.68 mL |  |
| TFA | 0.1 | 22.97 µL | 29.977 mL |  |
|  | 0.5 | 114.86 µL | 29.885 mL |  |
|  | 1.0 | 229.7 µL | 29.770 mL |  |
|  | 5.0 | 1.149 mL | 28.851 mL |  |
|  | 7.0 | 1.608 mL | 28.392 mL |  |
|  | 10 | 2.297 mL | 27.703 mL |  |

## Supplementary Table 6. Summary of BET surface area of Ce-FMAs and other MOFs

| **Modulator/Types of MOF** | **Ratio of modulator/ligand** | **BET surface area (multipoint BET method)**  **/(m^2^/g)** |
| --- | --- | --- |
| AA | 0.1 | 71.46 |
|  | 0.5 | 92.92 |
|  | 1.0 | 69.02 |
|  | 5.0 | 117.38 |
|  | 7.0 | 168.74 |
|  | 10 | 152.04 |
|  | 20 | 188.64 |
|  | 50 | 294.90 |
|  | 70 | 183.81 |
|  | 100 | 156.27 |
| FA | 0.1 | 94.39 |
|  | 0.5 | 84.82 |
|  | 1.0 | 99.83 |
|  | 5.0 | 144.41 |
|  | 7.0 | 148.16 |
|  | 10 | 140.04 |
|  | 20 | 120.43 |
|  | 50 | 50.18 |
|  | 70 | 127.18 |
|  | 100 | 44.79 |
| TFA | 0.1 | 117.08 |
|  | 0.5 | 167.58 |
|  | 1.0 | 165.38 |
|  | 5.0 | 237.59 |
|  | 7.0 | 173.20 |
|  | 10 | 125.32 |
| Ce-UiO-66-unactiviated | | 473.56 |
| Ce-UiO-66-activiated | | 517.00 |
| Zr-UiO-66 | | 425.71 |
| Hf-UiO-66 | | 312.06 |
| MOF-808 | | 1017.89 |

## Supplementary Table 7. Summary of conversion rate after 12 h towards various phosphate substrates

| **Substrate** | **Conversion rate after 12 h (%)** | **Optimized pH** |
| --- | --- | --- |
|  |  |  |
| AMP | 4.987261 | 7.0 |
| ADP | 18.45223 | 10.0 |
| ATP | 21.46497 | 10.0 |
| β-GP | 7.253185 | 7.0 |

## Supplementary Table 8. Summary of half-life towards different MOFs and ALP

| **MOF/ALP** | **Half-life towards pNPP** | **Comment** |
| --- | --- | --- |
| Hf-FMA | more than 1 h |  |
| Hf-BDC | more than 1 h |  |
| Zr-FMA | appr. 50 min |  |
| Zr-BDC | appr. 6 min |  |
| Ce-BDC | within 2 min | activated |
| Ce-FMA | within 2 min |  |
| ALP | appr. 7.5 min | 1 U/mL |
| ALP | appr. 10 min | 0.5 U/mL |

## Supplementary Table 9. Comparison between trypsin and Ce-FMA-FA-20-RT

| **Substance** | **Storage** | **Cost** | | **Conversion** | **Ratio of mass specific conversion** |
| --- | --- | --- | --- | --- | --- |
|  |  | **RMB/mg** | **Ratio of cost** |  |  |
| Trypsin | -20 °C | 0.3 | 1.15×10^4^ | 37 °C, 1 mg/mL, 1 d, 100% | 35 |
| Ce-FMA-FA-20-RT | room temperature | 0.000026 | 1 | 37 °C, 5 mg/mL, 7 d, 100% | 1 |
|  |  |  |  | 60 °C, 5 mg/mL, 1.5 d, 100% | 4.67 |

# Supplementary Discussion

## Supplementary discussion 1

As shown in Supplementary Figure 1, during the process of breaking a phosphate bond, though involved in various substrates, a catalyst (usually with Lewis acid sites) activates the central phosphorus first, which enhances the affinity for later nucleophile attack. Given the shared catalytic mechanism, we think it is reasonable to make such compare kinetic data towards different substrates.

## Supplementary discussion 2

First, Ce-FMA was synthesized without modulators to exam which temperature and reaction time could yield crystalline products. Products were collected after 10 min of stirring at room temperature and subsequent stirring for a longer time under reflux (around 105 °C). X-ray diffraction (XRD) results confirmed that the crystal structure remained uniform from 10 min at room temperature to 180 min under reflux (Supplementary Figure 5a); additionally, the activity of these MOFs towards the hydrolysis of para-nitrophenyl phosphate (pNPP) remained almost identical over reaction time (Supplementary Figure 5b). Therefore, stirring at room temperature for 10 min without the additional high-temperature step was applied in subsequent trials to evaluate the effect of modulators (see the synthetic details in Supplementary Table 5).

## Supplementary discussion 3

XRD results in Supplementary Figures 6a, 6b and 6c showed that Ce-FMAs modulated by different acids with various ratios retained the same crystalline structure. However, when the strongest acid TFA was applied, no crystal could be obtained with ratio greater than 10, which is consistent with previous report^29^.

## Supplementary discussion 4

In brief, monocarboxylic acids at low concentration usually slow down the process of crystalline, resulting in smaller size; while at high concentration, monocarboxylic acids accelerate crystal growth, making particles larger (see in Supplementary Figure 7). Moreover, the stronger acid is, the lower critical ratio is. For example, MOF modulated by AA with ratio of 20 is as large as FA with ratio of 10.

## Supplementary discussion 5

Since FMA is shorter than BDC in length, the unit cell constants of FMA linked MOFs are smaller than BDC linked MOFs. Thus, XRD patters for FMA based MOFs shifted into right (larger angle) compared with BDC based MOFs in Supplementary Figure 9a.

## Supplementary discussion 6

According to the optimization in Supplementary Figure 12, finally, pH 10.0 and concentration of 10 mM pNPP were optimized for later experiments.

## Supplementary discussion 7

BNPP underwent a fast spontaneous hydrolysis at alkaline solutions or high concentration (Supplementary Figure 13b), indicating easier cleavable property than pNPP. Therefore, Ce-FMAs with good activity towards pNPP (modulated by FA with an FA-to-FMA ratio of 20) was still active when it hydrolyzed BNPP (Supplementary Figures 11d and 11e) and even showed faster kinetics.

## Supplementary discussion 8

As displayed in Supplementary Figure 18, the catalytic activity of Ce-FMA-FA-20-RT declined gradually in which it remained around 70% activity in the second use but dropped to around 30% in the third use. We attributed the moderate recyclability to two reasons: (1) the phosphate induced catalyst poisoning as the strong binding affinity between Zr/Ce and phosphate^30^; (2) Ce-FMA-FA-20-RT aggregated into larger particles as shown in Supplementary Figure 24c and 24d, thereby leading to the decreased activity.

## Supplementary discussion 9

Figures 4a and4b and Supplementary Figure 21 indicated no single ammonia acid product can be obtained. We attributed this no further hydrolysis behavior into two reasons: (1) larger peptides were adsorbed on the surface of Ce-FMA-FA-20-RT, as shown in Supplementary Figures 24e and 24f, which outlined the adsorbed peptides as white dashed lines. (2) the fragments detected by GPC peaking at 44-46 min were relatively smaller ones, which were released from the surface of Ce-FMA-FA-20-RT. Thus further hydrolysis into amino acids was hindered.

## Supplementary discussion 10

Likewise, Ce-FMA-FA-20-RT and other MOF-based protease-like nanozymes also have high affinity towards BSA/peptides^31^, resulting in low efficiency in recyclability. To exclude the possibility that the decrease of BSA curves run by GPC is due to the adsorption instead of degradation, we applied EDTA-Tris-HCl buffer (8.0) at 4 ºC for three days. As seen in Supplementary Figure 23a, the yellowish supernatant indicates the leakage of Ce^4+^ because EDTA will grab the metal ions from MOF. We also run a SDS-PAGE on the precipitation (Ce-FMA-FA-20-RT after reaction) collected from various time in Supplementary Figure 23b. Though BSA was adsorbed initially, the band to BSA finally degraded after 24 h (see the rectangle in Supplementary Figure 23b), proving that BSA was degraded by Ce-FMA-FA-20-RT gradually.

## Supplementary discussion 11

TEM images revealed a bit of aggregation of Ce-FMA-FA-20-RT after reacted with pNPP and BNPP. And there was protein/peptide like adsorption in the surface of Ce-FMA-FA-20-RT after reacted with BSA.

## Supplementary discussion 12

Even though Ce-FMA-FA-20-RT is able to cleave N-acetyl-β-D-glucosaminide (Supplementary Figure 25), it is still challenging to cleave disaccharides without acetyl group (Supplementary Figure 25).

## Supplementary discussion 13

Pearson’s absolute hardness is a measure of Lewis acid strength based on orbital thermodynamics and is well linked to efficiency of Lewis acid catalysis^32^. As shown in Supplementary Table 2, cerium (IV) and hafnium (IV) have by far the highest absolute hardness with zirconium (IV), chromium (III) in the second tier. Such a hardness trend led us to explore Ce/Hf/Zr composed MOFs as hydrolytic nanozymes. Cr was not studied due to its potential toxicity concern.

# Supplementary Methods

## Chemical and biological reagents

Ce(NH_4_)_2_(NO_3_)_6_, acetic acid (HOAc), sodium acetate (NaOAc), fumaric acid (FMA), N,N-dimethylformamide (DMF), chitosan (dissolve in pH=4.0 HOAc-NaOAc buffer), formic acid (FA), trifluoroacetic acid (TFA), HCl, and 4-(2-hydroxyethyl)piperazine-1-ethanesulfonic acid (HEPES) were purchased from SCR HUSHI. Carboxymethyl chitosan (dissolved in pH=8.0 Tris-HCl buffer), CH_3_COONH_4_, benzene-1,4-dicarboxylic acid (H_2_BDC), p-nitrophenyl phosphate (pNPP), bis(4-nitrophenyl) phosphate (BNPP), and ZrOCl_2_·8H_2_O were purchased from Aladdin. 2-(cyclohexylamino)ethanesulfonic acid (CHES), crystal violet, and amylose from potato were purchased from Macklin. Tris (hydroxymethyl) aminomethane was purchased from Beijing Dingguo Changsheng Biotechnology Co. Ltd. HfCl_4_ was purchased from Sigma. Phosphate buffered saline, PBS (pH=7.2-7.4) was from CooLaber. Bovine serum albumin (BSA) was purchased from Biosharp. Pullulan was provided by Shodex, ranging from P5~P800 (*Mp* is 6,300, 9,800, 22,000, 49,400, 106,000, 201,000, 334,000 and 642,000 in order). All reagents were used without further purification. All aqueous solutions were prepared with double distilled water (18.2 MΩ·cm; Milli-Q).

*S. aureus* (Staphylococcus aureus, NCTC 10788) and *E. coli* (Escherichia coli, strain, DH5a) were used in bacterial experiments. Lysogeny broth (LB, QDRS BioTECH) and Agar powder (Chembase) were used to culture bacteria. Plasmid Mini Kit I 100 (Omega Bio-tek) was applied to extract plasmid DNA from *E. coli*. The concentration of DNA was measured by Tecan Pro 200 Microplate Reader equipped with Nanoquart. Agarose G-10 (Biowest), super GelRed (US Everbright@Inc), 50×TAE (Solarbio), DNA loading buffer (Solarbio), and 1 kb DNA ladder (Solarbio) were used to run agarose gel electrophoresis.

## Instrumentation

Ion chromatography was carried out with Thermo Dionex ICS-5000+ with CarboPac PA100 (4×250 mm). GPC (Gel permeation chromatography) profiles for monitoring hydrolytic process of macromolecules were recorded by using DGU-20A 3R (Shimadzu, Japan) with tandem columns SB-802.5 HQ and SB-804 HQ equipped with RID, UV detectors. GPC samples of BSA and amylose were run in 0.1 M CH_3_COONH_4_ as mobile phase; samples of chitosan were conducted in pure water while samples of carboxymethyl chitosan were in Tris-HCl at pH 8.0. A standard molecular weight calibration curve of GPC was collected by using Pullulan in various mobile phases. UV−visible absorption spectra were measured on Cary UV-Vis 100 (Agilent Technologies). The absorbance of the 96-well plates was read by using Tecan Pro 200 Microplate Reader. The crystalline phases of materials were identified by X-ray diffraction (XRD; Rigaku Corporation, Ultima III, Japan) with 2 °/min using a Cu Kα radiation. Morphology of materials/fixed bacteria were imaged by JEM-2100 and Zeiss Ultra 55 at an accelerating voltage of 10 kV. Prior to the SEM (scanning electronic microscopy) imaging, samples were sputter-coated with Cr to increase conductivity. Thermo-gravimetric analyses (TGA) were performed by using a TG 209 (Netzsch) in the temperature from ambient temperature to 800 °C under air flow with a heating rate of 10 °C/min. The Brunauer-Emmett-Teller (BET) surface areas were calculated from N_2_ sorption isotherms, which were obtained at 77 K by using Biaode Kubo X1000. Agarose gel electrophoresis of DNA was operated with Tanon 1200 and imaged by using Gel image analysis system (Tanon 1600).

## Hydrolysis of phosphate monoester bond (ALP-like activity, pNPP as the substrate)

***Optimization of pH.*** Different buffer medium including water, HEPES (pH=7.0, 7.5), PBS (pH=7.2-7.4), Tris-HCl (pH=8.0, 8.5), and CHES (pH=9.0, 9.5, 10.0) were investigated. Typically, 200 µL of buffer containing 0.5 mg/mL catalyst (FA-20-RT) and 10 mM pNPP was incubated at 37 °C for 30 min in 96 wells with 3 parallel samples. The absorbance at 400 nm was recorded by using Tecan Pro 200 Microplate Reader to indicate the activity on hydrolysis of pNPP.

***Optimization of concentration.*** Likewise, the optimization of pNPP concentration was conducted in 96 wells with 6 parallel samples containing pNPP with a concentration of 0.5 mM, 1 mM, 2 mM, 5 mM, 10 mM, and 20 mM in the presence of 0.5 mg/mL catalyst (FA-20-RT) in CHES (pH=10.0). After incubation for 10 min at 37 °C, the absorbance at 400 nm was recorded by using Tecan Pro 200 Microplate Reader.

## Hydrolysis of phosphodiester bond (phosphodiesterase-like activity, BNPP as the substrate)

***Optimization of pH.*** Likewise, different buffer medium including water, HEPES (pH=7.0, 7.5), PBS (pH=7.2-7.4), Tris-HCl (pH=8.0, 8.5), and CHES (pH=9.0, 9.5, 10.0) were investigated. Typically, 200 µL of buffer containing 0.5 mg/mL catalyst and 0.8 mM BNPP was incubated at 37 °C for 3 h in 96 wells with 4 parallel samples. The activity on hydrolysis of BNPP was read at the absorbance at 400 nm by Tecan Pro 200 Microplate Reader.

***Optimization of concentration.*** Since BNPP tends to hydrolyze spontaneously in high concentration under alkaline environment, we applied rather low concentration of BNPP (0.8 mM) in later experiments and the reaction time was prolonged to 3h.

## Hydrolysis of lipid (cephalin)

100 µL of cephalin (3.46 mg/mL) was mixed with 16.7 µL of Ce-FMA-FA-20-RT (10 mg/mL) at 37 °C in different buffers (HEPES 7.0 and 7.5, Tris-HCl 8.0 and 8.5, CHES 9.0, 9.5 and 10.0). After 12 h, 100 µL of the resulted solution were incubated with 100 µL molybdenum-blue chromogenic agent at 37 °C for 30 min^10^. Finally, the absorbance at 820 nm was recorded to calculate the conversion rate.

## Hydrolysis of plasmid DNA (plasmid extracted from *E. coli*)

Hydrolysis of DNA was conducted in 1×PBS at 37 °C with addition of 10 ng DNA and 5 μg Ce-FMA**-**FA-20-RT. Equivalent samples were taken at 12 h and 24 h, respectively. The hydrolytic results were observed by horizontal electrophoresis.

## Hydrolysis of lactose and maltose

Typically, 0.5 mL of 1.2%_wt_ lactose/maltose was mixed with or without 100 µL of 10 mg/mL Ce-FMA-FA-20-RT in pH 4 and pH 8 buffers at 60 °C with stirring. The obtained solutions were then centrifuged to collect the supernatants. After filtered by 0.22 µm filter, samples were run in 150 mM NaOH at 30 °C with a flow rate of 1.0 mL/min by ion chromatography to study whether their monosaccharides were generated by Ce-FMA-FA-20-RT.

## Bacterial culture and development of biofilm

***Culture of bacteria.*** Frozen *E. coli* and *S. aureus* were seeded in LB culture medium overnight at 37 °C with shaking at 220 rpm. Then the cultured solution was spread onto LB agar plate to obtain single colony. Next day, single colony was transferred into fresh LB culture medium until the absorbance at 600 nm achieved around 1.0 for use.

***Development and dispersion of biofilm.*** To develop biofilm, the cultured solution was diluted into the absorbance at 600 nm around 0.01 (cuvette with 1 cm optical path). Then 2 mL of bacterial solution was seeded in 24-well plate at 37 °C still for 48 h. Fresh medium was renewed every 24 h. For dispersing biofilm of *S. aureus*, each group (ctrl and 50 μg/mL Ce-FMA-FA-20-RT (treated group) was conducted with 16 parallel samples at 37 °C for 12 h. For statistical analysis, significance was established by using t tests (and nonparametric tests) performed with GraphPad Prism 6. The highest value and the lowest value were removed when analyzing.

Likewise, the dispersion biofilm of *E. coli* was conducted with 12 parallel samples at 37 °C for 12 h. For statistical analysis, significance was established by using t tests (and nonparametric tests) performed with GraphPad Prism 6. The highest value and lowest value were removed when analyzing.

## Cytotoxicity test of Ce-FMA-FA-20-RT treated *E. coli* & *S. aureus*

After the monocolony of *E. coli* and *S. aureus* achieved 1.0 at the absorbance at 600 nm (cuvette with 1 cm optical path), the bacteria solution was diluted to 10^4^ times. Then 100 μL of diluted solution and 5 μL of Ce-FMA-FA-20-RT (10 mg/mL) or PBS (control group) was diluted into 1 mL solution with fresh medium, respectively. The mixture was placed at 37 °C with shaking at 220 rpm. After 30 min, 100 μL of Ce-FMA-FA-20-RT treated bacteria was spread into LB agar plates and cultured at 37 °C for 24 h with three parallel plates.

# Supplementary References

1 Katz, M. J. et al. One step backward is two steps forward: Enhancing the hydrolysis rate of UiO-66 by decreasing [OH^–^]. *ACS Catal.* **5**, 4637-4642, (2015).

2 Kalinovskyy, Y., Cooper, N. J., Main, M. J., Holder, S. J. & Blight, B. A. Microwave-assisted activation and modulator removal in zirconium MOFs for buffer-free CWA hydrolysis. *Dalton Trans.* **46**, 15704-15709, (2017).

3 Wang, S., Bromberg, L., Schreuder-Gibson, H. & Hatton, T. A. Organophophorous ester degradation by chromium(III) terephthalate metal–organic framework (MIL-101) chelated to N,N-dimethylaminopyridine and related aminopyridines. *ACS Appl. Mater. Interfaces* **5**, 1269-1278, (2013).

4 Peterson, G. W. & Wagner, G. W. Detoxification of chemical warfare agents by CuBTC. *J. Porous Mater.* **21**, 121-126, (2014).

5 Plonka, A. M. et al. In situ probes of capture and decomposition of chemical warfare agent simulants by Zr-based metal organic frameworks. *J. Am. Chem. Soc.* **139**, 599-602, (2017).

6 Li, P. et al. Synthesis of nanocrystals of Zr-based metal–organic frameworks with csq-net: Significant enhancement in the degradation of a nerve agent simulant. *Chem. Commun.* **51**, 10925-10928, (2015).

7 de Koning, M. C., van Grol, M. & Breijaert, T. Degradation of Paraoxon and the chemical warfare agents VX, Tabun, and Soman by the metal–organic frameworks UiO-66-NH_2_, MOF-808, NU-1000, and PCN-777. *Inorg. Chem.* **56**, 11804-11809, (2017).

8 Park, H. J. et al. Synthesis of a Zr-based metal–organic framework with spirobifluorenetetrabenzoic acid for the effective removal of nerve agent simulants. *Inorg. Chem.* **56**, 12098-12101, (2017).

9 Xia, M. et al. Assembly of the active center of organophosphorus hydrolase in metal–organic frameworks via rational combination of functional ligands. *Chem. Commun.* **53**, 11302-11305, (2017).

10 Qin, L., Wang, X., Liu, Y. & Wei, H. 2D-metal-organic-framework-nanozyme sensor arrays for probing phosphates and their enzymatic hydrolysis. *Anal. Chem.* **90**, 9983-9989, (2018).

11 Lide, D. R. *CRC handbook of chemistry and physics*. Vol. 85 (CRC press, 2004).

12 Huheey, J. E., Keiter, E. A., Keiter, R. L. & Medhi, O. K. *Inorganic chemistry: principles of structure and reactivity*. (Pearson Education India, 2006).

13 James, A. M. & Lord, M. P. *Macmillan's chemical and physical data*. (Macmillan, 1992).

14 Li, Y. et al. H_5_PV_2_Mo_10_O_40_ encapsulated in MIL-101(Cr): Facile synthesis and characterization of rationally designed composite materials for efficient decontamination of sulfur mustardt. *Dalton Trans.* **47**, 6394-6403, (2018).

15 Li, B. et al. MOFzyme: Intrinsic protease-like activity of Cu-MOF. *Sci. Rep.* **4**, 6759, (2014).

16 Giannakoudakis, D. A., Hu, Y., Florent, M. & Bandosz, T. J. Smart textiles of MOF/g-C_3_N_4_ nanospheres for the rapid detection/detoxification of chemical warfare agents. *Nanoscale Horiz.* **2**, 356-364, (2017).

17 Mondloch, J. E. et al. Destruction of chemical warfare agents using metal–organic frameworks. *Nat. Mater.* **14**, 512-516, (2015).

18 Moon, S. Y. et al. Detoxification of chemical warfare agents using a Zr_6_-based metal–organic framework/polymer mixture. *Chem. Eur. J.* **22**, 14864-14868, (2016).

19 Liu, Y., Moon, S. Y., Hupp, J. T. & Farha, O. K. Dual-function metal–organic framework as a versatile catalyst for detoxifying chemical warfare agent simulants. *ACS Nano* **9**, 12358-12364, (2015).

20 Gil-San-Millan, R. et al. Chemical warfare agents detoxification properties of zirconium metal–organic frameworks by synergistic incorporation of nucleophilic and basic sites. *ACS Appl. Mater. Interfaces* **9**, 23967-23973, (2017).

21 López-Maya, E. et al. Textile/metal–organic-framework composites as self-detoxifying filters for chemical-warfare agents. *Angew. Chem. Int. Ed.* **54**, 6790-6794, (2015).

22 Zhao, J. et al. Ultra-fast degradation of chemical warfare agents using MOF-nanofiber kebabs. *Angew. Chem. Int. Ed.* **55**, 13224-13228, (2016).

23 Katz, M. J. et al. Exploiting parameter space in MOFs: A 20-fold enhancement of phosphate-ester hydrolysis with UiO-66-NH_2_. *Chem. Sci.* **6**, 2286-2291, (2015).

24 Lammert, M. et al. Cerium-based metal organic frameworks with UiO-66 architecture: Synthesis, properties and redox catalytic activity. *Chem. Commun.* **51**, 12578-12581, (2015).

25 Lee, D. T., Zhao, J., Peterson, G. W. & Parsons, G. N. Catalytic “MOF-cloth” formed via directed supramolecular assembly of UiO-66-NH_2_ crystals on atomic layer deposition-coated textiles for rapid degradation of chemical warfare agent simulants. *Chem. Mater.* **29**, 4894-4903, (2017).

26 Nunes, P., Gomes, A. C., Pillinger, M., Goncalves, I. S. & Abrantes, M. Promotion of phosphoester hydrolysis by the Zr-IV-based metal–organic framework UiO-67. *Microporous Mesoporous Mater.* **208**, 21-29, (2015).

27 Asha, P., Sinha, M. & Mandal, S. Effective removal of chemical warfare agent simulants using water stable metal–organic frameworks: Mechanistic study and structure-property correlation. *RSC Adv.* **7**, 6691-6696, (2017).

28 Moon, S. Y. et al. Effective, facile, and selective hydrolysis of the chemical warfare agent VX using Zr_6_-based metal–organic frameworks. *Inorg. Chem.* **54**, 10829-10833, (2015).

29 Hu, Z. et al. Modulator effects on the water-based synthesis of Zr/Hf metal–organic frameworks: Quantitative relationship studies between modulator, synthetic condition, and performance. *Cryst. Growth Des.* **16**, 2295-2301, (2016).

30 Chen, X. et al. Formulation of metal–organic framework-based drug carriers by controlled coordination of methoxy PEG phosphate: boosting colloidal stability and redispersibility. *J. Am. Chem. Soc.* **143**, 13557-13572, (2021).

31 Loosen, A. et al. Interplay between structural parameters and reactivity of Zr_6_-based MOFs as artificial proteases. *Chem. Sci.* **11**, 6662-6669, (2020).

32 Parr, R. G. & Pearson, R. G. Absolute hardness: Companion parameter to absolute electronegativity. *J. Am. Chem. Soc.* **105**, 7512-7516, (1983).
