# Supplementary material for: Data-informed discovery of hydrolytic nanozymes
Source: Nat Commun. 2022 Feb 11;13:827. doi: 10.1038/s41467-022-28344-2 (PMC8837776; doi:10.1038/s41467-022-28344-2)
Supplement: Supplementary file 2 — Description of Additional Supplementary Files [file 41467_2022_28344_MOESM2_ESM.pdf]

Description of Additional Supplementary Files

File Name: Supplementary Data 1
Description: PDF file of one hundred and five references used for data analysis.
