# Supplementary material for: Data-informed discovery of hydrolytic nanozymes
Source: Nat Commun. 2022 Feb 11;13:827. doi: 10.1038/s41467-022-28344-2 (PMC8837776; doi:10.1038/s41467-022-28344-2)
Supplement: Supplementary file 3 — Supplementary Data 1 [file 41467_2022_28344_MOESM3_ESM.pdf]

One hundred and five references used for data analysis.

1 Almerindo, G. I. et al. Propanolysis of methyl paraoxon in the presence of aluminum-titanate-supported erbium oxide. *J. Phys. Chem. C* **120**, 22323-22329, (2016).

2 Asha, P., Sinha, M. & Mandal, S. Effective removal of chemical warfare agent simulants using water stable metal–organic frameworks: Mechanistic study and structure–property correlation. *RSC Adv.* **7**, 6691-6696, (2017).

3 Balow, R. B. et al. Environmental effects on zirconium hydroxide nanoparticles and chemical warfare agent decomposition: Implications of atmospheric water and carbon dioxide. *ACS Appl. Mater. Interfaces* **9**, 39747-39757, (2017).

4 Bermudez, V. M. Investigation of the interaction of gamma-Al_2O_3 with aqueous solutions of dimethyl methylphosphonate using infrared multiple internal reflection spectroscopy. *Langmuir* **29**, 1483-1489, (2013).

5 Bisio, C. et al. Nanosized inorganic metal oxides as heterogeneous catalysts for the degradation of chemical warfare agents. *Catal. Today* **277**, 192-199, (2016).

6 Bobbitt, N. S. et al. Metal-organic frameworks for the removal of toxic industrial chemicals and chemical warfare agents. *Chem. Soc. Rev.* **46**, 3357-3385, (2017).

7 Bonomi, R., Scrimin, P. & Mancin, F. Phosphate diesters cleavage mediated by Ce(IV) complexes self-assembled on gold nanoparticles. *Org. Biomol. Chem.* **8**, 2622-2626, (2010).

8 Bonomi, R. et al. Phosphate diester and DNA hydrolysis by a multivalent, nanoparticle-based catalyst. *J. Am. Chem. Soc.* **130**, 15744-15745, (2008).

9 Breaker, R. R. & Joyce, G. F. A DNA enzyme that cleaves RNA. *Chemistry Biology* **1**, 223-229, (1994).

10 Breslow, R. Artificial enzymes. *Science* **218**, 532-537, (1982).

11 Chapleski, R. C., Jr., Musaev, D. G., Hill, C. L. & Troya, D. Reaction mechanism of nerve-agent hydrolysis with the $Cs_8Nb_6O_{19}$ Lindqvist hexaniobate catalyst. *J. Phys. Chem. C* **120**, 16822-16830, (2016).

12 Chen, H., Liao, P., Mendonca, M. L. & Snurr, R. Q. Insights into catalytic hydrolysis of organophosphate warfare agents by metal-organic framework NU-1000. *J. Phys. Chem. C* **122**, 12362-12368, (2018).

13 Chen, Z. W. et al. Design of surface-active artificial enzyme particles to stabilize pickering emulsions for high-performance biphasic biocatalysis. *Adv. Mater.* **28**, 1682-1688, (2016).

14 Chinthakindi, S. et al. Iron oxide functionalized graphene nano-composite for dispersive solid phase extraction of chemical warfare agents from aqueous samples. *J. Chromatogr. A* **1394**, 9-17, (2015).

15 de Koning, M. C., van Grol, M. & Breijaert, T. Degradation of paraoxon and the chemical warfare agents VX, Tabun, and Soman by the metal-organic frameworks UiO-66-NH_2, MOF-808, NU-1000, and PCN-777. *Inorg. Chem.* **56**, 11804-11809, (2017).

16 Decoste, J. B. & Peterson, G. W. Metal-organic frameworks for air purification of toxic chemicals. *Chem. Rev.* **114**, 5695-5727, (2014).

17 Diez Castellnou, M. *Strategies for the modulation of catalytic activity of Zn(II)-based artificial nucleases*. (2014).

18 Diez-Castellnou, M., Mancin, F. & Scrimin, P. Efficient phosphodiester cleaving nanozymes resulting from multivalency and local medium polarity control. *J. Am. Chem. Soc.* **136**, 1158-1161, (2014).

19 Du, B., Li, D., Wang, J. & Wang, E. Designing metal-contained enzyme mimics for prodrug activation. *Adv. Drug Deliv. Rev.* **118**, 78-93, (2017).

20 Florent, M., Giannakoudakis, D. A., Wallace, R. & Bandosz, T. J. Mixed CuFe and ZnFe (hydr)oxides as reactive adsorbents of chemical warfare agent surrogates. *J. Hazard. Mater.* **329**, 141-149, (2017).

21 Fridkin, G., Yehezkel, L., Columbus, I. & Zafrani, Y. Solvent effects on the reactions of the nerve agent VX with KF/Al_2O_3: Heterogeneous or homogeneous decontamination? *J. Org. Chem.* **81**, 2154-2158, (2016).

22 Giannakoudakis, D. A., Arcibar-Orozco, J. A. & Bandosz, T. J. Effect of GO phase in $Zn(OH)_2$/GO composite on the extent of photocatalytic reactive adsorption of mustard gas surrogate. *Appl. Catal. B-Environ.* **183**, 37-46, (2016).

23 Giannakoudakis, D. A. et al. Zinc peroxide nanoparticles: Surface, chemical and optical properties and the effect of thermal treatment on the detoxification of mustard gas. *Appl. Catal. B-Environ.* **226**, 429-440, (2018).

24 Giannakoudakis, D. A., Hu, Y., Florent, M. & Bandosz, T. J. Smart textiles of MOF/g-C_3N_4 nanospheres for the rapid detection/detoxification of chemical warfare agents. *Nanoscale Horiz.* **2**, 356-364, (2017).

25 Gil-San Millan, R. et al. Chemical warfare agents detoxification properties of zirconium metal-organic frameworks by synergistic incorporation of nucleophilic and basic sites. *ACS Appl. Mater. Interfaces* **9**, 23967-23973, (2017).

26 Gomes, A. C., Pillinger, M., Nunes, P., Goncalves, I. S. & Abrantes, M. Promotion of phosphoester hydrolysis by MoO_2Cl_2L (L bipyridine derivatives, H_2O, no ligand), $MoO_2(CH_3)_2L$ (L bipyridine derivatives) and related inorganice-organic hybrids in aqueous media. *J. Organomet. Chem.* **760**, 42-47, (2014).

27 Guerriertakada, C., Gardiner, K., Marsh, T., Pace, N. & Altman, S. The RNA moiety of ribonuclease-P is the catalytic subunit of the enzyme. *Cell* **35**, 849-857, (1983).

28 He, X., Zhang, F., Liu, J., Fang, G. & Wang, S. Homogenous graphene oxide-peptide nanofiber hybrid hydrogel as biomimetic polysaccharide hydrolase. *Nanoscale* **9**, 18066-18074, (2017).

29 Hirakawa, T. et al. Specific properties on TiO_2 photocatalysis to decompose isopropyl methylphosphonofluoridate and dimethyl methylphosphonate in gas phase. *J. Photochem. Photobiol. A-Chem.* **264**, 12-17, (2013).

30 Hostert, L. et al. Imidazole-derived graphene nanocatalysts for organophosphate destruction: Powder and thin film heterogeneous reactions. *J. Catal.* **356**, 75-84, (2017).

31 Housaindokht, M. R. & Zamand, N. A DFT study of associative and dissociative chemical adsorption of DMMP onto SnO_2(110) surface nano-cluster. *Struct. Chem.* **26**, 87-96, (2015).

32 Huang, X. L. Hydrolysis of phosphate esters catalyzed by inorganic iron oxide nanoparticles acting as biocatalysts. *Astrobiology* **18**, 294-310, (2018).

33 Huang, X. L. & Zhang, J. Z. Hydrolysis of glucose-6-phosphate in aged, acid-forced hydrolysed nanomolar inorganic iron solutions-an inorganic biocatalyst? *RSC Adv.* **2**, 199-208, (2012).

34 Islamoglu, T. et al. Cerium(IV) vs zirconium(IV) based metal–organic frameworks for detoxification of a nerve agent. *Chem. Mater.* **29**, 2672-2675, (2017).

35 Janos, P. et al. Cerium oxide for the destruction of chemical warfare agents: A comparison of synthetic routes. *J. Hazard. Mater.* **304**, 259-268, (2016).

36 Janos, P. et al. Cerium dioxide as a new reactive sorbent for fast degradation of parathion methyl and some other organophosphates. *J. Rare Earths* **32**, 360-370, (2014).

37 Janos, P. et al. Magnetically separable reactive sorbent based on the CeO_2/gamma-Fe_2O_3 composite and its utilization for rapid degradation of the organophosphate pesticide parathion methyl and certain nerve agents. *Chem. Eng. J.* **262**, 747-755, (2015).

38 Kalinovskyy, Y., Cooper, N. J., Main, M. J., Holder, S. J. & Blight, B. A. Microwave-assisted activation and modulator removal in zirconium MOFs for buffer-free CWA hydrolysis. *Dalton Trans.* **46**, 15704-15709, (2017).

39 Kim, S. et al. Zirconium hydroxide-coated nanofiber mats for nerve agent decontamination. *Chem. Asian J.* **12**, 698-705, (2017).

40 Kowsalya Vellingiri , L. P., Ki Hyun Kim. Metal–organic frameworks as media for the catalytic degradation. *Coord. Chem. Rev.* **353**, 159-179, (2017).

41 Kuchma, M. H. et al. Phosphate ester hydrolysis of biologically relevant molecules by cerium oxide nanoparticles. *Nanomed.* **6**, 738-744, (2010).

42 Kuo, L. Y., Baker, D. C., Dortignacq, A. K. & Dill, K. M. Phosphonothioate hydrolysis by molybdocene dichlorides: Importance of metal interaction with the sulfur of the thiolate leaving group. *Organometallics* **32**, 4759-4765, (2013).

43 Kuo, L. Y., Bennett, A. & Miao, Q. Heterogeneous organophosphate ethanolysis: Degradation of phosphonothioate neurotoxin by a supported molybdenum peroxo polymer. *Inorg. Chem.* **56**, 10013-10020, (2017).

44 Lammert, M. et al. Cerium-based metal organic frameworks with UiO-66 architecture: synthesis, properties and redox catalytic activity. *Chem. Commun.* **51**, 12578-12581, (2015).

45 Lee, D. T., Zhao, J., Peterson, G. W. & Parsons, G. N. Catalytic "MOF-cloth" formed via directed supramolecular assembly of UiO-66-NH_2 crystals on atomic layer deposition-coated textiles for rapid degradation of chemical warfare agent simulants. *Chem. Mater.* **29**, 4894-4903, (2017).

46 Lee, S. Y., Lee, S., Lee, J., Lee, H. S. & Chang, J. H. Biomimetic magnetic nanoparticles for rapid hydrolysis of ester compounds. *Materials Letters* **110**, 229-232, (2013).

47 Li, B. et al. MOFzyme: Intrinsic protease-like activity of Cu-MOF. *Sci. Rep.* **4**, 6759, (2014).

48 Li, P. et al. Synthesis of nanocrystals of Zr-based metal-organic frameworks with csq-net: significant enhancement in the degradation of a nerve agent simulant. *Chem. Commun.* **51**, 10925-10928, (2015).

49 Li, P. et al. Encapsulation of a nerve agent detoxifying enzyme by a mesoporous zirconium metal-organic framework engenders thermal and long-term stability. *J. Am. Chem. Soc.* **138**, 8052-8055, (2016).

50 Li, Y. et al. $H_5PV_2Mo_{10}O_{40}$ encapsulated in MIL-101(Cr): Facile synthesis and characterization of rationally designed composite materials for efficient decontamination of sulfur mustardt. *Dalton Trans.* **47**, 6394-6403, (2018).

51 Lin, Y. H., Ren, J. S. & Qu, X. G. Nano-gold as artificial enzymes: Hidden talents. *Adv. Mater.* **26**, 4200-4217, (2014).

52 Liu, Y., Moon, S.-Y., Hupp, J. T. & Farha, O. K. Dual-function metal-organic framework as a versatile catalyst for detoxifying chemical warfare agent simulants. *ACS Nano* **9**, 12358-12364, (2015).

53 Ma, X. J. et al. Mimicking the active sites of organophosphorus hydrolase on the backbone of graphene oxide to destroy nerve agent simulants. *ACS Appl. Mater. Interfaces* **9**, 21089-21093, (2017).

54 Manea, F., Houillon, F. B., Pasquato, L. & Scrimin, P. Nanozymes: Gold-nanoparticle-based transphosphorylation catalysts. *Angew. Chem. Int. Ed.* **43**, 6165-6169, (2004).

55 Martin, M. et al. Metallodendrimers as transphosphorylation catalysts. *J. Am. Chem. Soc.* **129**, 6982-6983, (2007).

56 Mathew, D., Thomas, B. & Devaky, K. S. Amidase activity of phosphonate analogue imprinted chymotrypsin mimics in shape-selective, substrate-specific and enantioselective amidolysis of l-phenylalanine-p-nitroanilides. *J. Mol. Catal. A: Chem.* **415**, 65-73, (2016).

57 McCarthy, D. L. et al. Electrospun metal–organic framework polymer composites for the catalytic degradation of methyl paraoxon. *New J. Chem.* **41**, 8748-8753, (2017).

58 Molenveld, P., Engbersen, J. F. J. & Reinhoudt, D. N. Dinuclear metallo-phosphodiesterase models: Application of calix[4]arenes as molecular scaffolds. *Chem. Soc. Rev.* **29**, 75-86, (2000).

59 Mondloch, J. E. et al. Destruction of chemical warfare agents using metal–organic frameworks. *Nat. Mater.* **14**, 512-516, (2015).

60 Moon, S. Y. et al. Detoxification of chemical warfare agents using a Zr-6-based metal-organic framework/polymer mixture. *Chem.-Eur. J.* **22**, 14864-14868, (2016).

61 Moon, S. Y. et al. Effective, facile, and selective hydrolysis of the chemical warfare agent VX using Zr_6-based metal-organic frameworks. *Inorg. Chem.* **54**, 10829-10833, (2015).

62 Naseri, M. T., Sarabadani, M., Ashrafi, D., Saeidian, H. & Babri, M. Photoassisted and photocatalytic degradation of sulfur mustard using TiO_2 nanoparticles and polyoxometalates. *Environ. Sci. Pollut. Res.* **20**, 907-916, (2013).

63 Nunes, P., Gomes, A. C., Pillinger, M., Goncalves, I. S. & Abrantes, M. Promotion of phosphoester hydrolysis by the Zr-IV-based metal-organic framework UiO-67. *Microporous Mesoporous Mater.* **208**, 21-29, (2015).

64 Park, H. J. et al. Synthesis of a Zr-based metal-organic framework with spirobifluorenetetrabenzoic acid for the effective removal of nerve agent simulants. *Inorg. Chem.* **56**, 12098-12101, (2017).

65 Pasquato, L., Rancan, F., Scrimin, P., Mancin, F. & Frigeri, C. N-methylimidazole-functionalized gold nanoparticles as catalysts for cleavage of a carboxylic acid ester. *Chem. Commun.*, 2253-2254, (2000).

66 Peterson, G. W., Lu, A. X. & Epps, T. H., 3rd. Tuning the morphology and activity of electrospun polystyrene/UiO-66-NH_2 metal-organic framework composites to enhance chemical warfare agent removal. *ACS Appl. Mater. Interfaces* **9**, 32248-32254, (2017).

67 Peterson, G. W. & Wagner, G. W. Detoxification of chemical warfare agents by CuBTC. *J. Porous Mater.* **21**, 121-126, (2014).

68 Pezzato, C. & Prins, L. J. Transient signal generation in a self-assembled nanosystem fueled by ATP. *Nat. Commun.* **6**, 7790, (2015).

69 Pieters, G., Pezzato, C. & Prins, L. J. Controlling supramolecular complex formation on the surface of a monolayer-protected gold nanoparticle in water. *Langmuir* **29**, 7180-7185, (2013).

70 Plonka, A. M. et al. In situ probes of capture and decomposition of chemical warfare agent simulants by Zr-based metal organic frameworks. *J. Am. Chem. Soc.* **139**, 599-602, (2017).

71 Praveen Kumar, J., Prasad, G. K., Ramacharyulu, P. V. R. K., Garg, P. & Ganesan, K. Mesoporous CuO-ZnO binary metal oxide nanocomposite for decontamination of sulfur mustard. *Mater. Chem. Phys.* **142**, 484-490, (2013).

72 Ramacharyulu, P. V. R. K. & Prasad, G. K. Enhanced photocatalytic activity of mesoporous nano titania decorated with zinc phthalocyanine. *Indian J. Chem. Sect A-Inorg. Bio-Inorg. Phys. Theor. Anal. Chem.* **57**, 18-25, (2018).

73 Ramacharyulu, P. V. R. K. et al. Sunlight assisted photocatalytic detoxification of sulfur mustard on vanadium ion doped titania nanocatalysts. *J. Mol. Catal. A: Chem.* **387**, 38-44, (2014).

74 Sahu, C., Ghosh, D., Sen, K. & Das, A. K. Decomposition of O,S-dimethyl methylphosphonothiolate by ammonia on magnesium oxide: a theoretical study of catalytic detoxification of a chemical warfare agent. *Phys. Chem. Chem. Phys.* **17**, 20231-20249, (2015).

75 Salvio, R. & Cincotti, A. Guanidine based self-assembled monolayers on Au nanoparticles as artificial phosphodiesterases. *RSC Adv.* **4**, 28678-28682, (2014).

76 Savelli, C. & Salvio, R. Guanidine-based polymer brushes grafted onto silica nanoparticles as efficient artificial phosphodiesterases. *Chem.-Eur. J.* **21**, 5856-5863, (2015).
77 Sharma, P. K. et al. Photoelectrocatalytic degradation of blistering agent sulfur mustard to non-blistering substances using pPy/NiOBPC nanocomposite. *J. Mol. Catal. A-Chem.* **366**, 368-374, (2013).
78 Silva, V. B., Rodrigues, T. S., Camargo, P. H. C. & Orth, E. S. Detoxification of organophosphates using imidazole-coated Ag, Au and AgAu nanoparticles. *RSC Adv.* **7**, 40711-40719, (2017).
79 Simona Neri, S. G. M., Cristian Pezzato, Leonard J. Prins. Photoswitchable catalysis by a nanozyme mediated by a light-sensitive cofactor. *J. Am. Chem. Soc.* **139**, 1794-1797, (2017).
80 Singh, V. V. et al. Multifunctional silver-exchanged zeolite micromotors for catalytic detoxification of chemical and biological threats. *Adv. Funct. Mater.* **25**, 2147-2155, (2015).
81 Stengl, V., Grygar, T. M., Oplustil, F. & Olsanska, M. Decontamination of sulfur mustard from printed circuit board using Zr-doped titania suspension. *Ind. Eng. Chem. Res.* **52**, 3436-3440, (2013).
82 Tang, X. et al. Adsorption and decomposition of dimethyl methylphosphonate on size-selected $(MoO_3)_3$ clusters. *Phys. Chem. Chem. Phys.* **20**, 4840-4850, (2018).
83 Verma, A. K. et al. Alumina-supported oxime for the degradation of sarin and diethylchlorophosphate. *Chemosphere* **90**, 2254-2260, (2013).
84 Vernekar, A. A., Das, T. & Mugesh, G. Vacancy engineered nanoceria enzyme mimetic hotspots for the degradation of nerve agents. *Angew. Chem. Int. Ed.* **55**, 1412-1416, (2016).
85 Wallace, R., Giannakoudakis, D. A., Florent, M., Karwacki, C. J. & Bandosz, T. Ferrihydrite deposited on cotton textiles as protection media against the chemical warfare agent surrogate (2-chloroethyl ethyl sulfide). *J. Mater. Chem. A* **5**, 4972-4981, (2017).
86 Wan, D., Li, W., Wang, G., Lu, L. & Wei, X. Degradation of p-nitrophenol using magnetic Fe^0/Fe_3O_4/Coke composite as a heterogeneous Fenton-like catalyst. *Sci. Total Environ.* **574**, 1326-1334, (2017).
87 Wang, G. et al. Mechanism and kinetics for reaction of the chemical warfare agent simulant, DMMP(g), with zirconium(IV) MOFs: An ultrahigh-vacuum and DFT study. *J. Phys. Chem. C* **121**, 11261-11272, (2017).
88 Wang, Q. et al. Atomic-level structural dynamics of polyoxoniobates during DMMP decomposition. *Sci. Rep.* **7**, 773, (2017).
89 Wang, S., Bromberg, L., Schreuder-Gibson, H. & Hatton, T. A. Organophophorous ester degradation by chromium(III) terephthalate metal-organic framework (MIL-101) chelated to N, N-dimethylaminopyridine and related aminopyridines. *ACS Appl. Mater. Interfaces* **5**, 1269-1278, (2013).
90 Wang, T., Wang, J., Yang, Y., Su, P. & Yang, Y. Co_3O_4/reduced graphene oxide nanocomposites as effective phosphotriesterase mimetics for degradation and detection of Paraoxon. *Ind. Eng. Chem. Res.* **56**, 9762-9769, (2017).
91 Wong, Y.-M., Hoshino, Y., Sudesh, K., Miura, Y. & Numata, K. Optimization of poly(N-isopropylacrylamide) as an artificial amidase. *Biomacromolecules* **16**, 411-421, (2015).
92 Wong, Y. M., Masunaga, H., Chuah, J. A., Sudesh, K. & Numata, K. Enzyme-mimic peptide assembly to achieve amidolytic activity. *Biomacromolecules* **17**, 3375-3385, (2016).
93 Xia, M. et al. Assembly of the active center of organophosphorus hydrolase in metal-organic frameworks via rational combination of functional ligands. *Chem. Commun.* **53**, 11302-11305, (2017).
94 Xu, C., Liu, Z., Wu, L., Ren, J. S. & Qu, X. G. Nucleoside triphosphates as promoters to enhance nanoceria enzyme-like activity and for single- nucleotide polymorphism typing. *Adv. Funct. Mater.* **24**, 1624-1630, (2014).

95 Xu, C. & Qu, X. G. Recent advances of ceria-based biomedical applications. *Scientia Sinica Chimica*, 506-520 (2014).
96 Xu, P., Guo, S., Yu, H. & Li, X. Mesoporous silica nanoparticles (MSNs) for detoxification of hazardous organophorous chemicals. *Small* **10**, 2404-2412, (2014).
97 Yu, L. *Analysis of metal complexes of tetraazamoxa crown ether derivatives as simulated nucleases*, Chongqing University of Technology, (2015).
98 Zaupa, G., Mora, C., Bonomi, R., Prins, L. J. & Scrimin, P. Catalytic self-assembled monolayers on au nanoparticles: The source of catalysis of a transphosphorylation reaction. *Chem.-Eur. J.* **17**, 4879-4889, (2011).
99 Zhang, Q. et al. Artificial hydrolase based on carbon nanotubes conjugated with peptides. *Nanoscale* **8**, 16851-16856, (2016).
100 Zhang, Z. et al. Construction of the active site of metalloenzyme on Au NC micelles. *Chin. J. Chem.* **27**, 1215-1220, (2009).
101 Zhang, Z. et al. Self-assembled gold nanocrystal micelles act as an excellent artificial nanozyme with ribonuclease activity. *J. Biol. Inorg. Chem.* **14**, 653-662, (2009).
102 Zhao, J. et al. Ultra-fast degradation of chemical warfare agents using MOF-nanofiber kebabs. *Angew. Chem. Int. Ed.* **55**, 13224-13228, (2016).
103 Zhou, C. S., Liu, Q. L., Xu, W., Wang, C. R. & Fang, X. H. A water-soluble C_{60}-porphyrin compound for highly efficient DNA photocleavage. *Chem. Commun.* **47**, 2982-2984, (2011).
104 Zimmermann, L. M. et al. Degradation of methyl paraoxon in the presence of Mg^{2+}-Al^{3+} mixed oxides. *J. Phys. Chem. C* **117**, 26097-26105, (2013).
105 Q., Z. *Study on artificial hydrolase based on peptides and multiwalled carbon nanotube conjugations*, Tianjin University of Science and Technology, (2017).
