# Supplementary figures and images for: Data-informed discovery of hydrolytic nanozymes
Source: Nat Commun. 2022 Feb 11;13:827. doi: 10.1038/s41467-022-28344-2 (PMC8837776; doi:10.1038/s41467-022-28344-2)

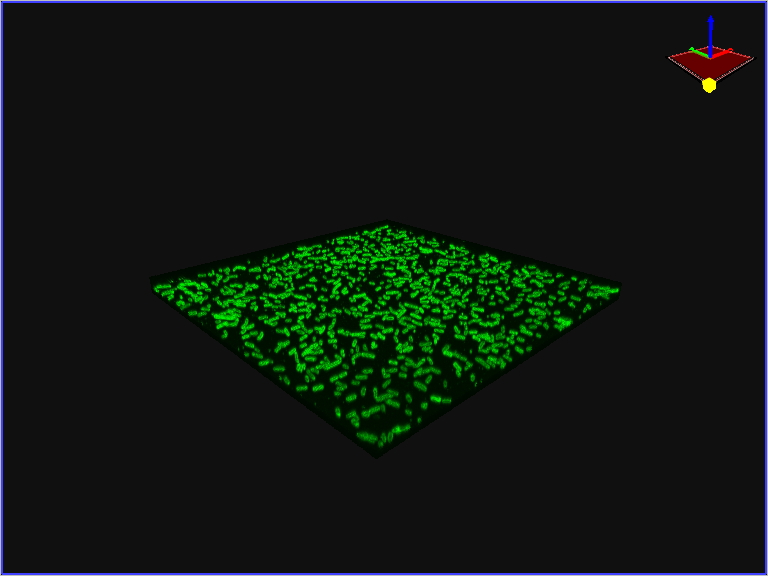

Supplement: Supplementary file 4 — Source Data [file 41467_2022_28344_MOESM4_ESM.zip › Source Data_NCOMMS-21-01890A/Fig. 6/Fig. 6b/ctrl-many_0003_green.bmp]

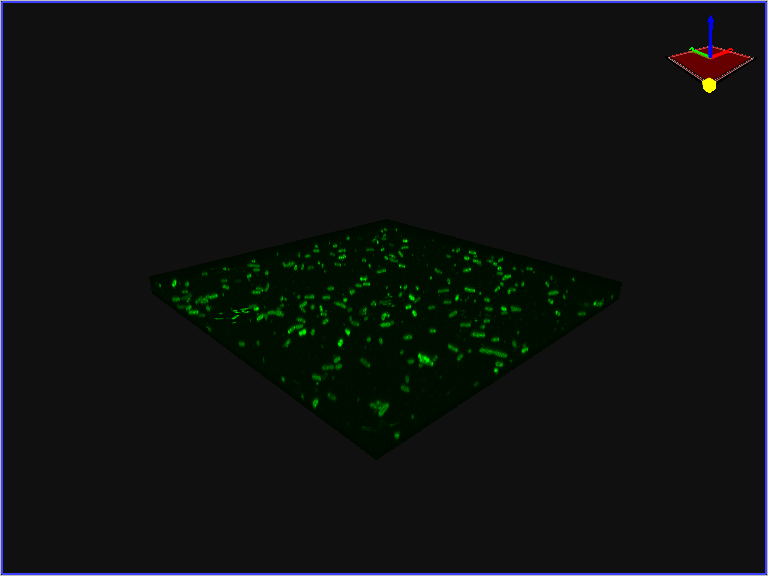

Supplement: Supplementary file 4 — Source Data [file 41467_2022_28344_MOESM4_ESM.zip › Source Data_NCOMMS-21-01890A/Fig. 6/Fig. 6b/treated-many_0004_green.bmp]

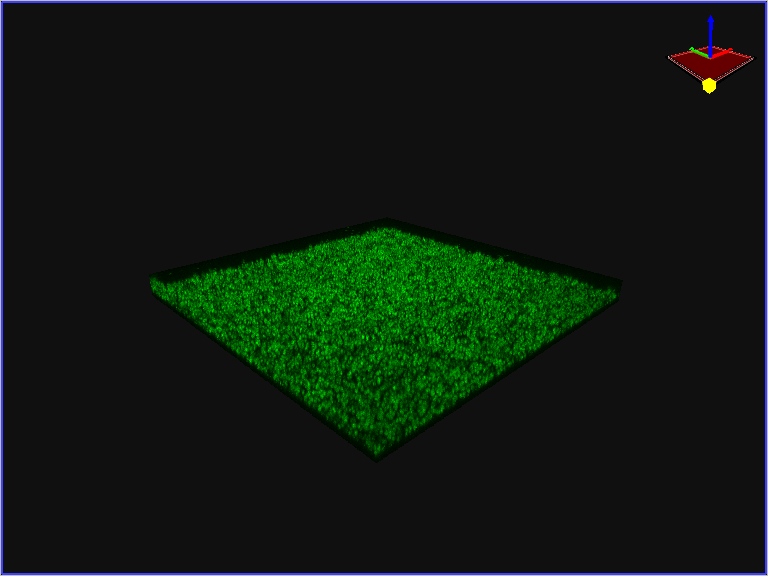

Supplement: Supplementary file 4 — Source Data [file 41467_2022_28344_MOESM4_ESM.zip › Source Data_NCOMMS-21-01890A/Fig. 6/Fig. 6d/ctrl_0004-green-stain.tif]

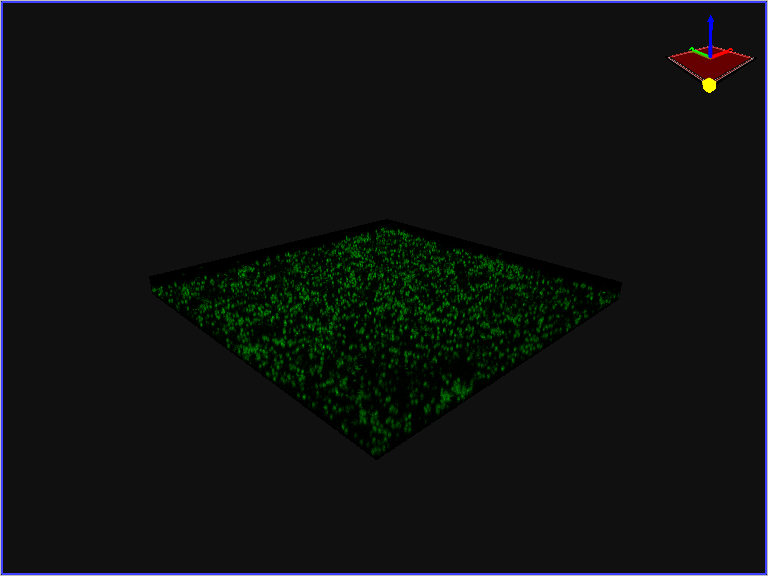

Supplement: Supplementary file 4 — Source Data [file 41467_2022_28344_MOESM4_ESM.zip › Source Data_NCOMMS-21-01890A/Fig. 6/Fig. 6d/treated-2_0003-green-stain.tif]

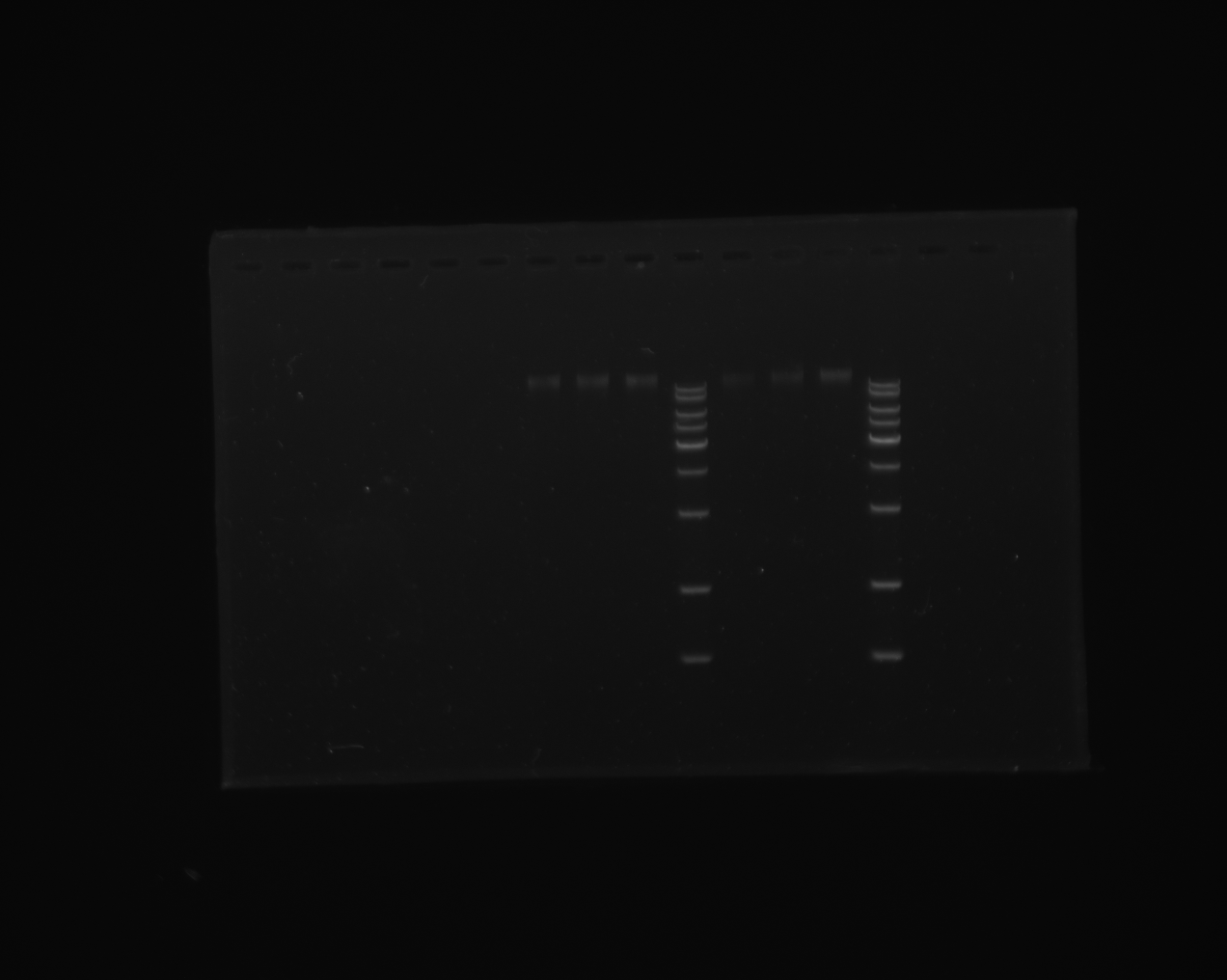

Supplement: Supplementary file 4 — Source Data [file 41467_2022_28344_MOESM4_ESM.zip › Source Data_NCOMMS-21-01890A/Fig. S16/jiaxianan-water-12h & 24 h.tif]

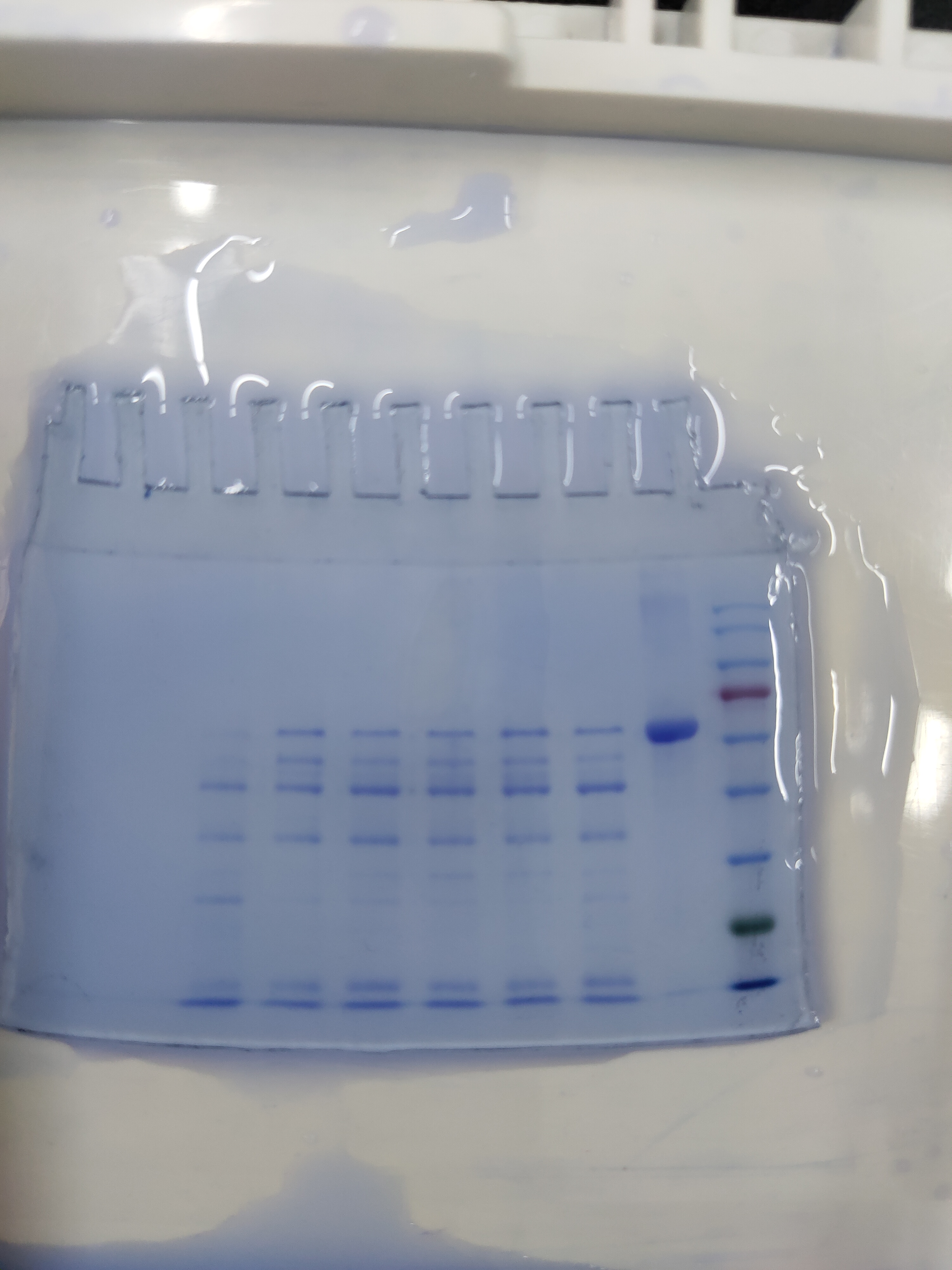

Supplement: Supplementary file 4 — Source Data [file 41467_2022_28344_MOESM4_ESM.zip › Source Data_NCOMMS-21-01890A/Fig. S19, Table S9/trypsin-1mg-24h-3.jpg]

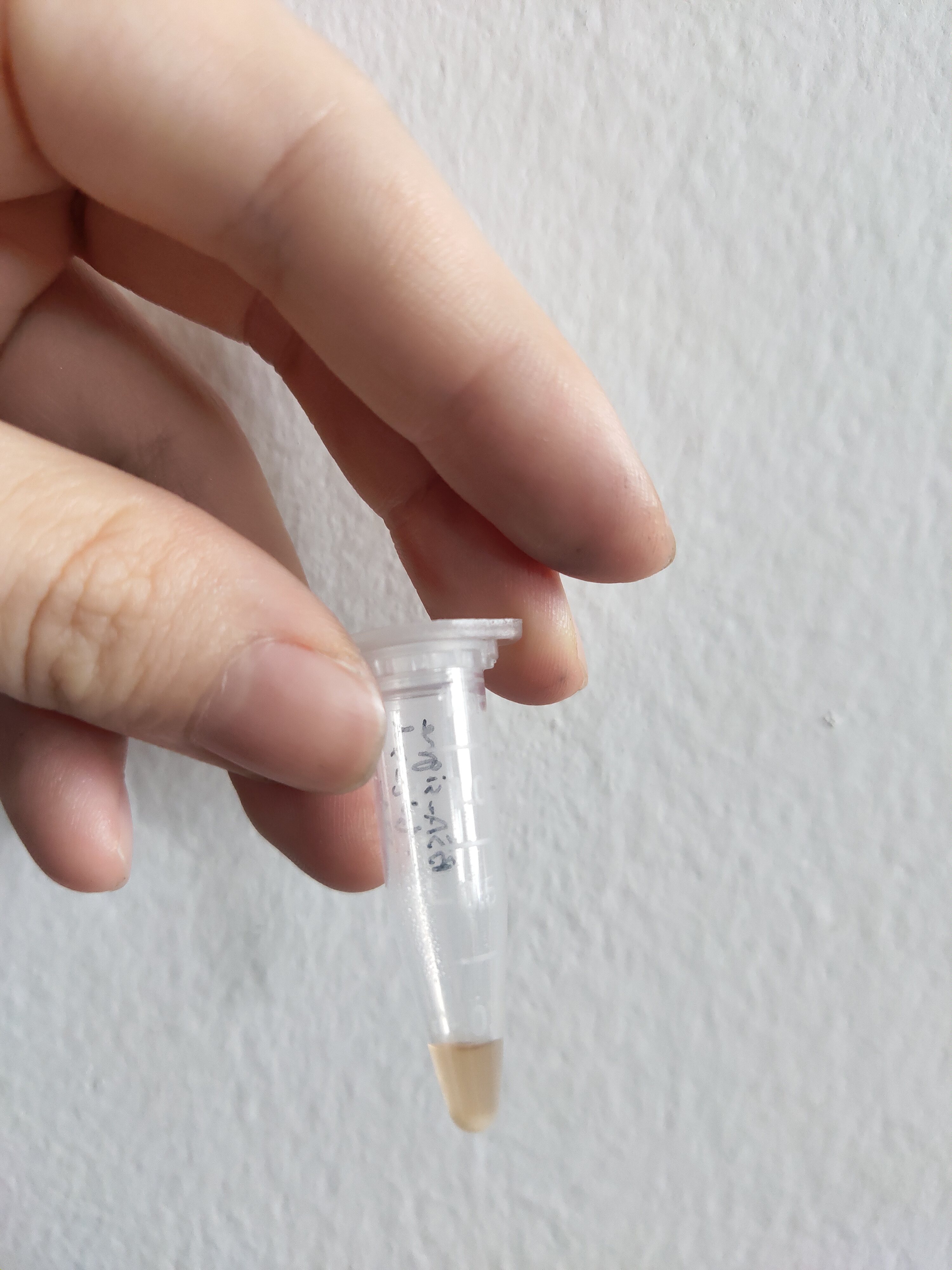

Supplement: Supplementary file 4 — Source Data [file 41467_2022_28344_MOESM4_ESM.zip › Source Data_NCOMMS-21-01890A/Fig. S23/S23a.jpg]

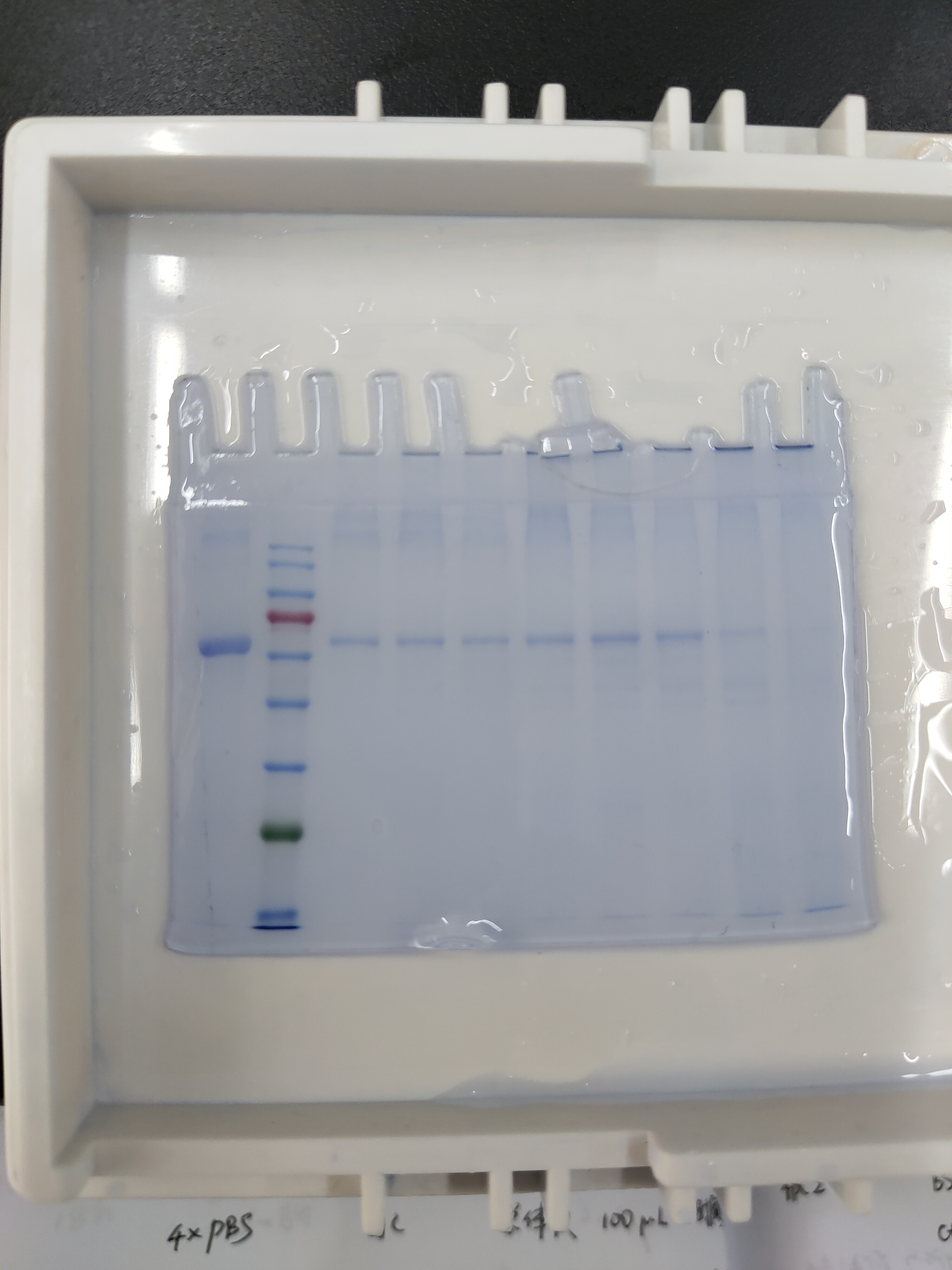

Supplement: Supplementary file 4 — Source Data [file 41467_2022_28344_MOESM4_ESM.zip › Source Data_NCOMMS-21-01890A/Fig. S23/S23b.jpg]

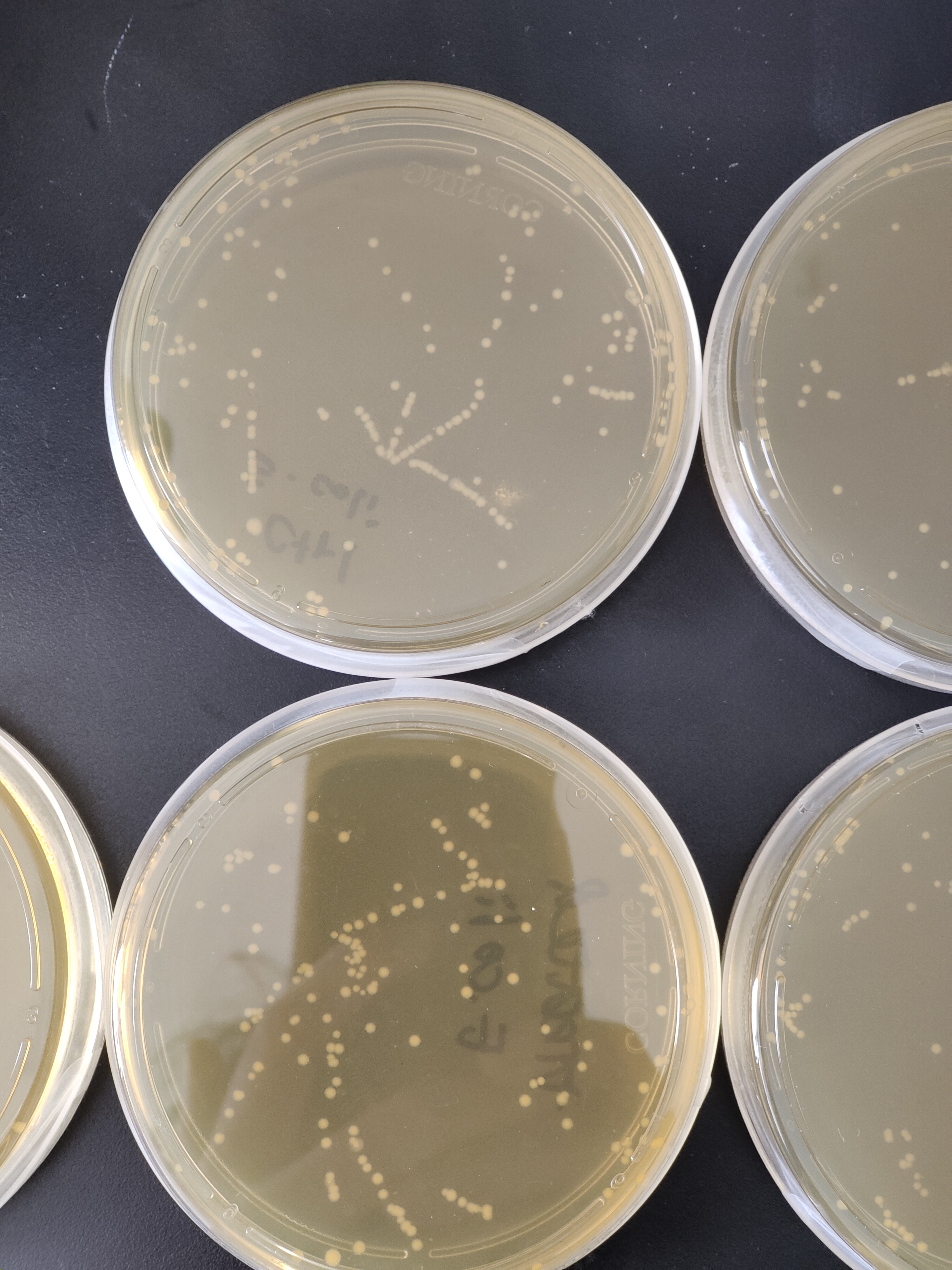

Supplement: Supplementary file 4 — Source Data [file 41467_2022_28344_MOESM4_ESM.zip › Source Data_NCOMMS-21-01890A/Fig. S29/E. coli/ctrl-1.jpg]

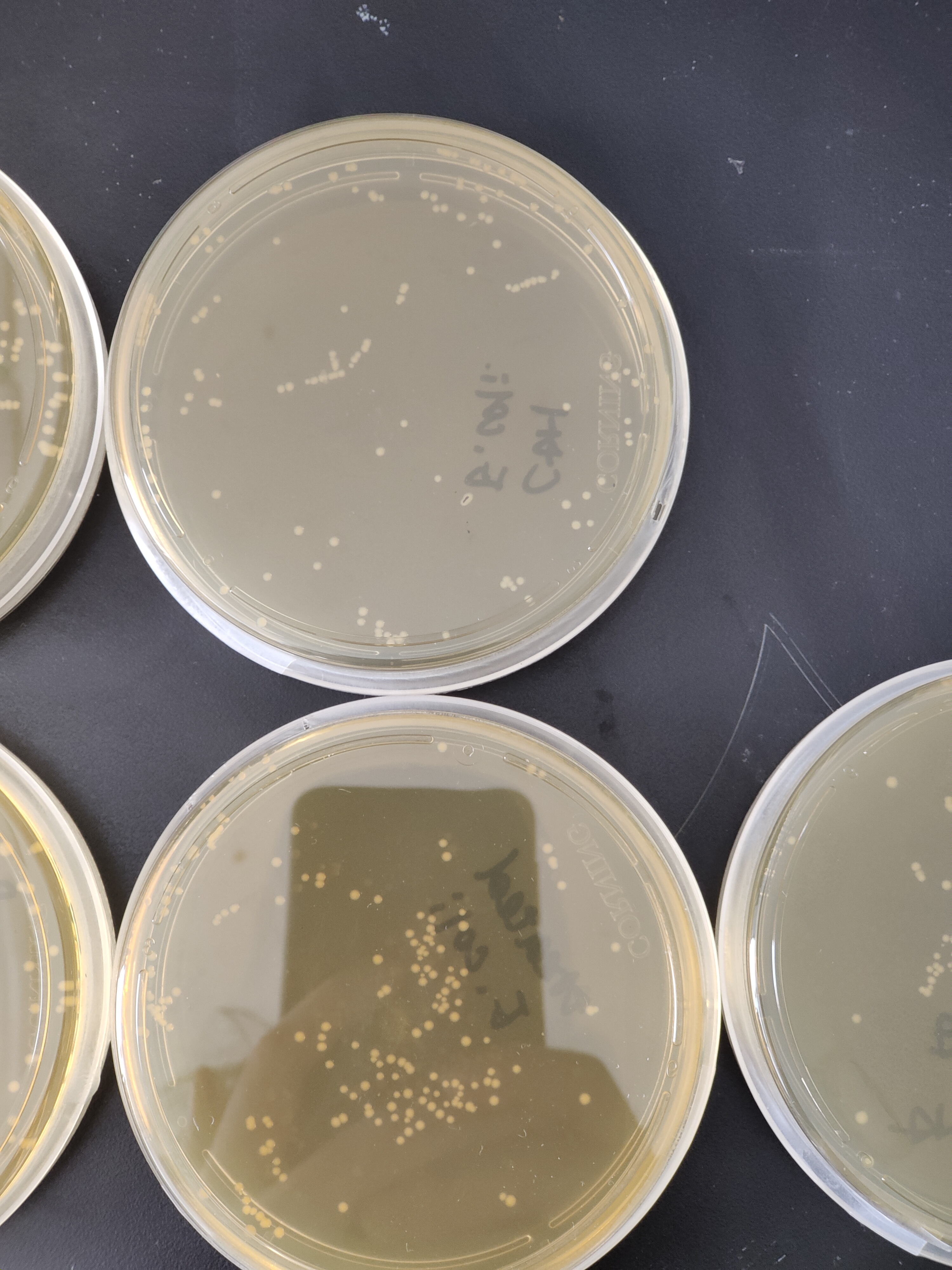

Supplement: Supplementary file 4 — Source Data [file 41467_2022_28344_MOESM4_ESM.zip › Source Data_NCOMMS-21-01890A/Fig. S29/E. coli/ctrl-2.jpg]

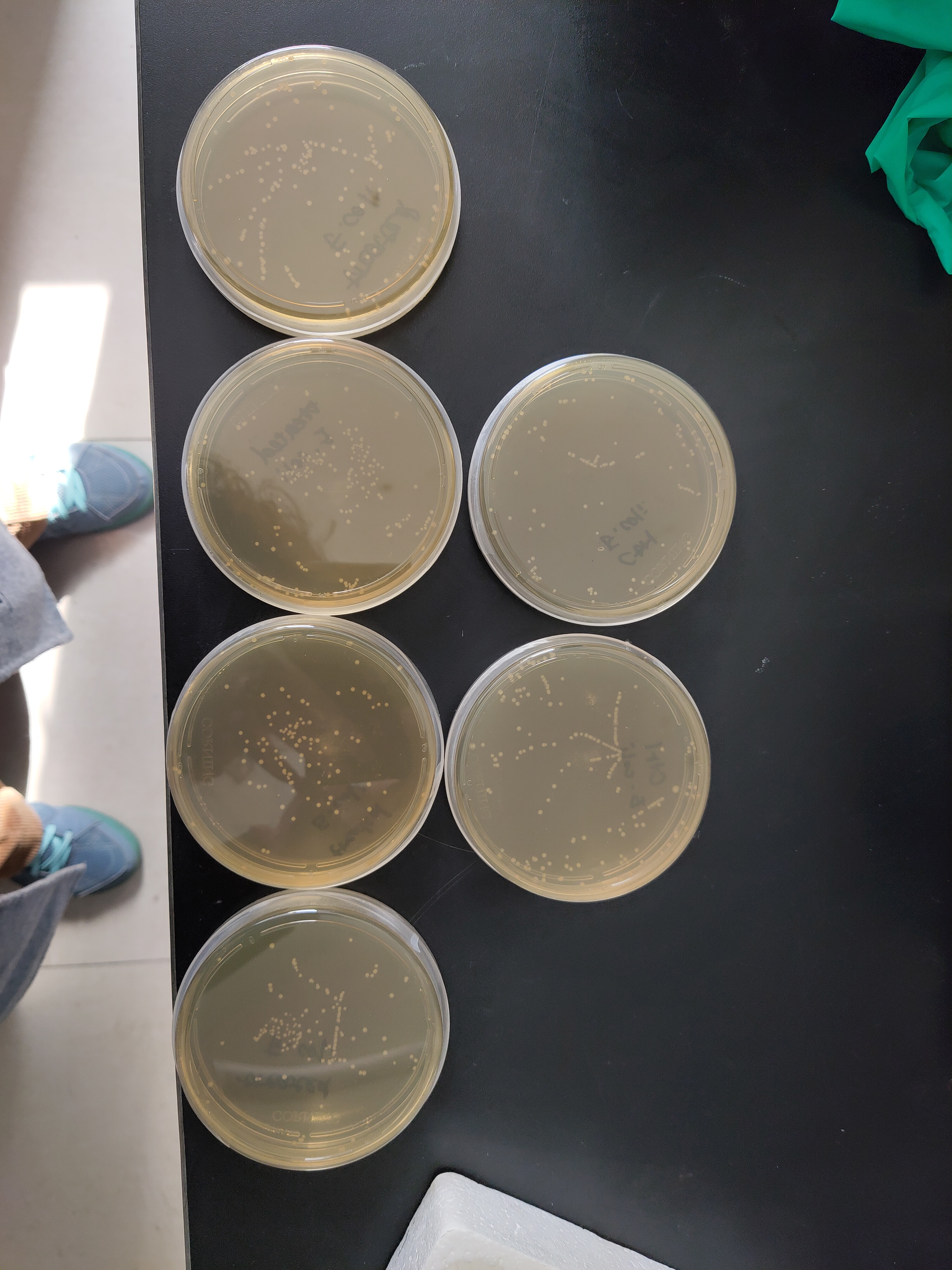

Supplement: Supplementary file 4 — Source Data [file 41467_2022_28344_MOESM4_ESM.zip › Source Data_NCOMMS-21-01890A/Fig. S29/E. coli/overall.jpg]

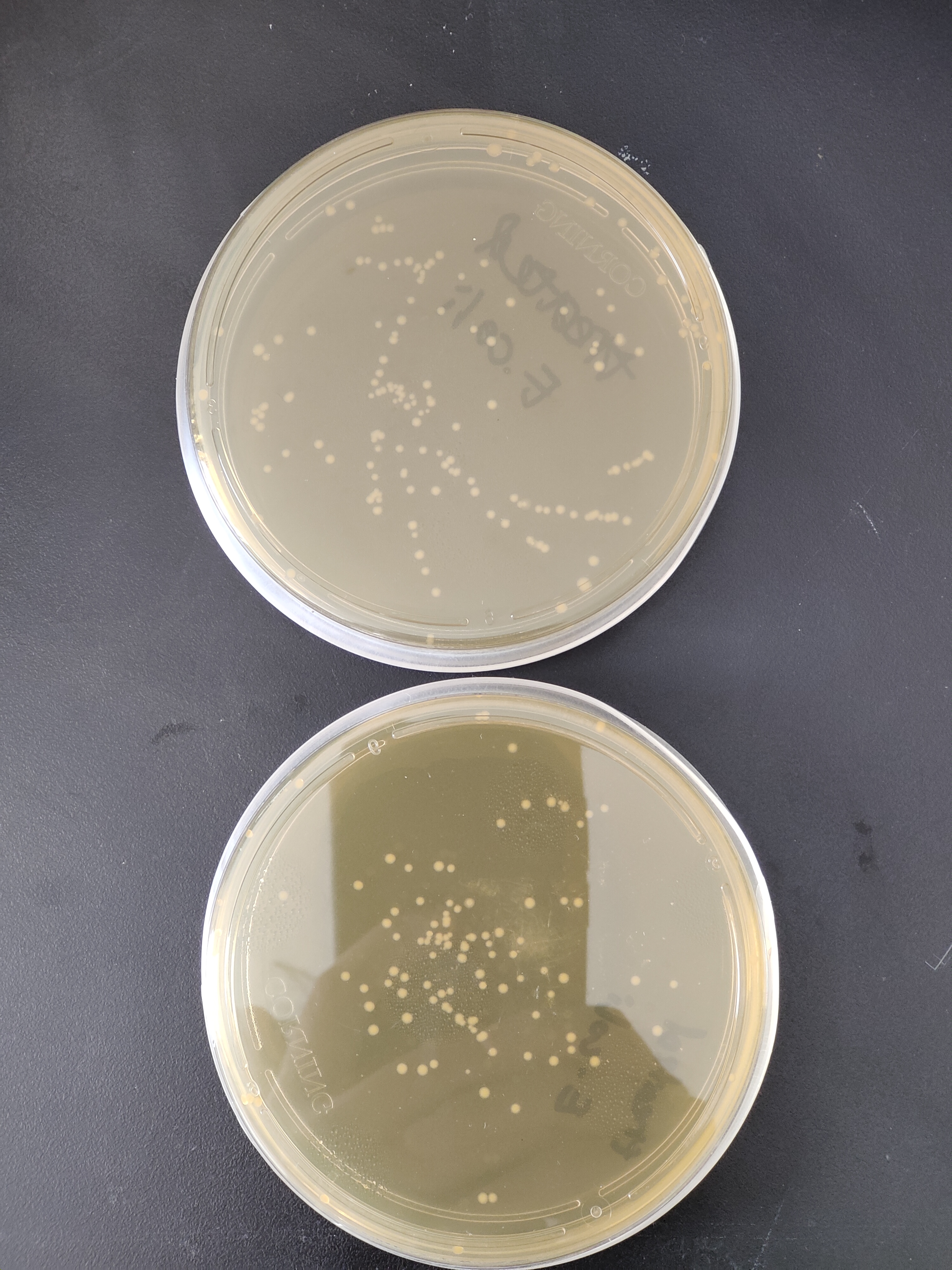

Supplement: Supplementary file 4 — Source Data [file 41467_2022_28344_MOESM4_ESM.zip › Source Data_NCOMMS-21-01890A/Fig. S29/E. coli/treated-1.jpg]

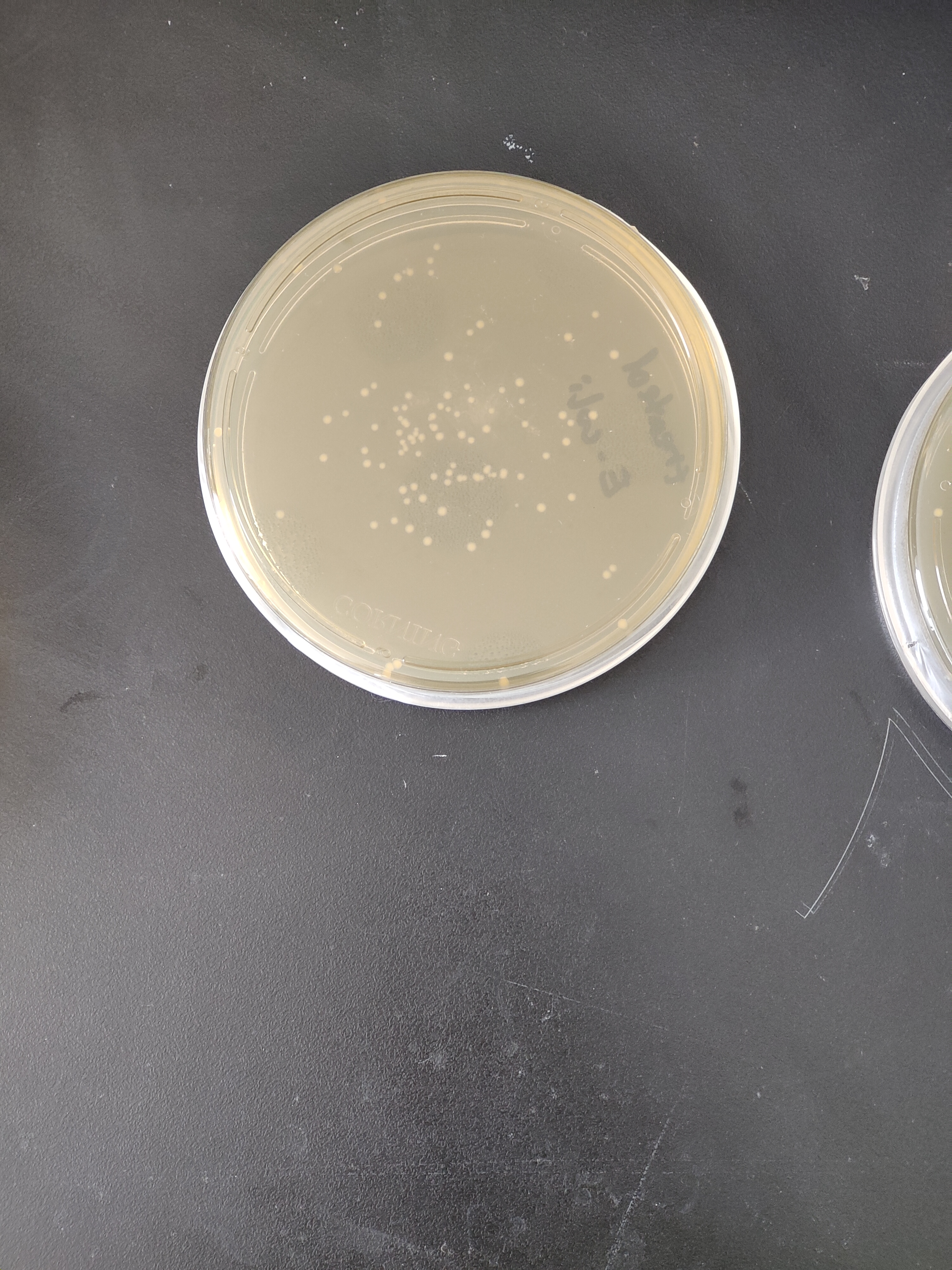

Supplement: Supplementary file 4 — Source Data [file 41467_2022_28344_MOESM4_ESM.zip › Source Data_NCOMMS-21-01890A/Fig. S29/E. coli/treated-2.jpg]

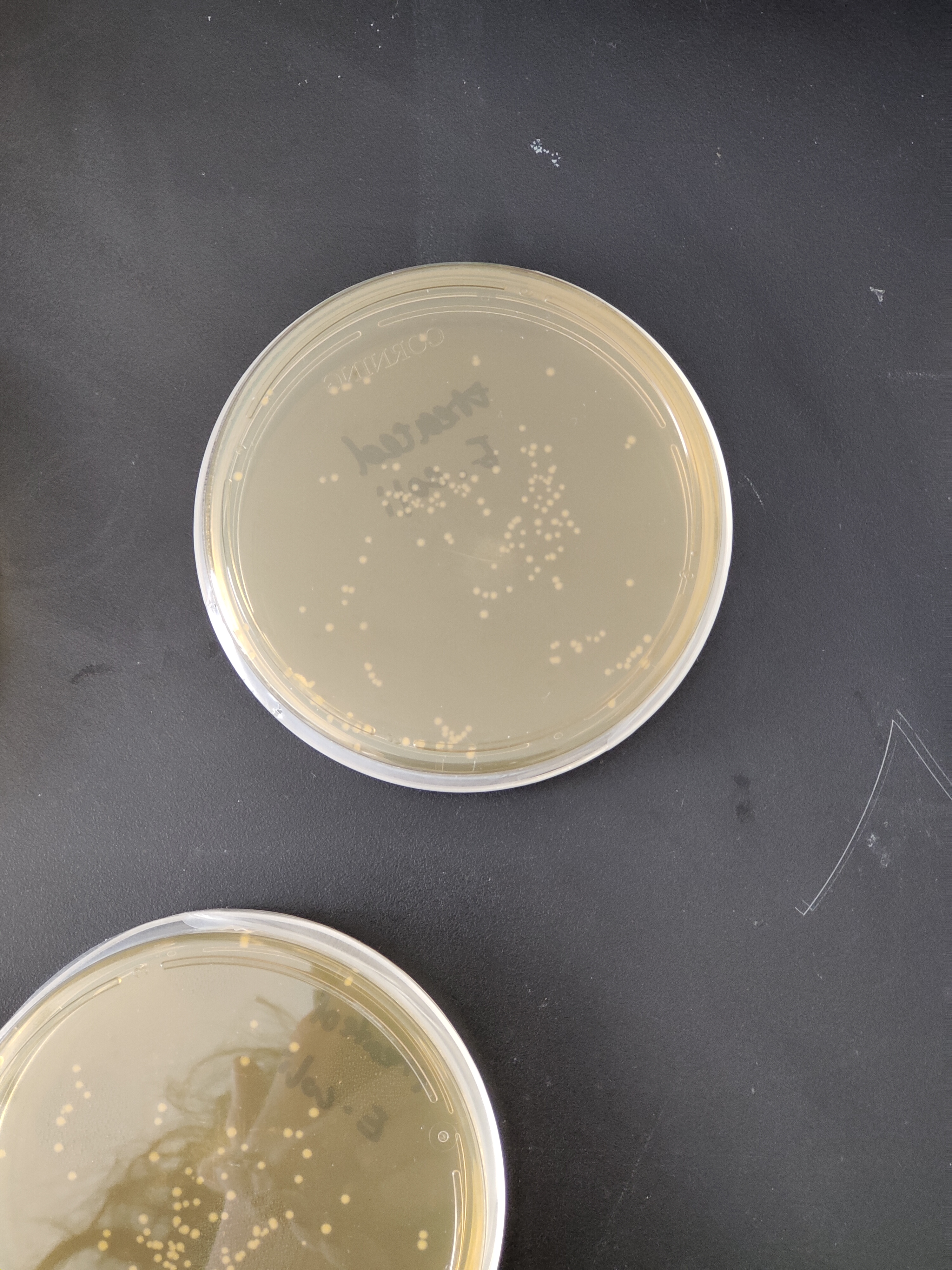

Supplement: Supplementary file 4 — Source Data [file 41467_2022_28344_MOESM4_ESM.zip › Source Data_NCOMMS-21-01890A/Fig. S29/E. coli/treated-3.jpg]

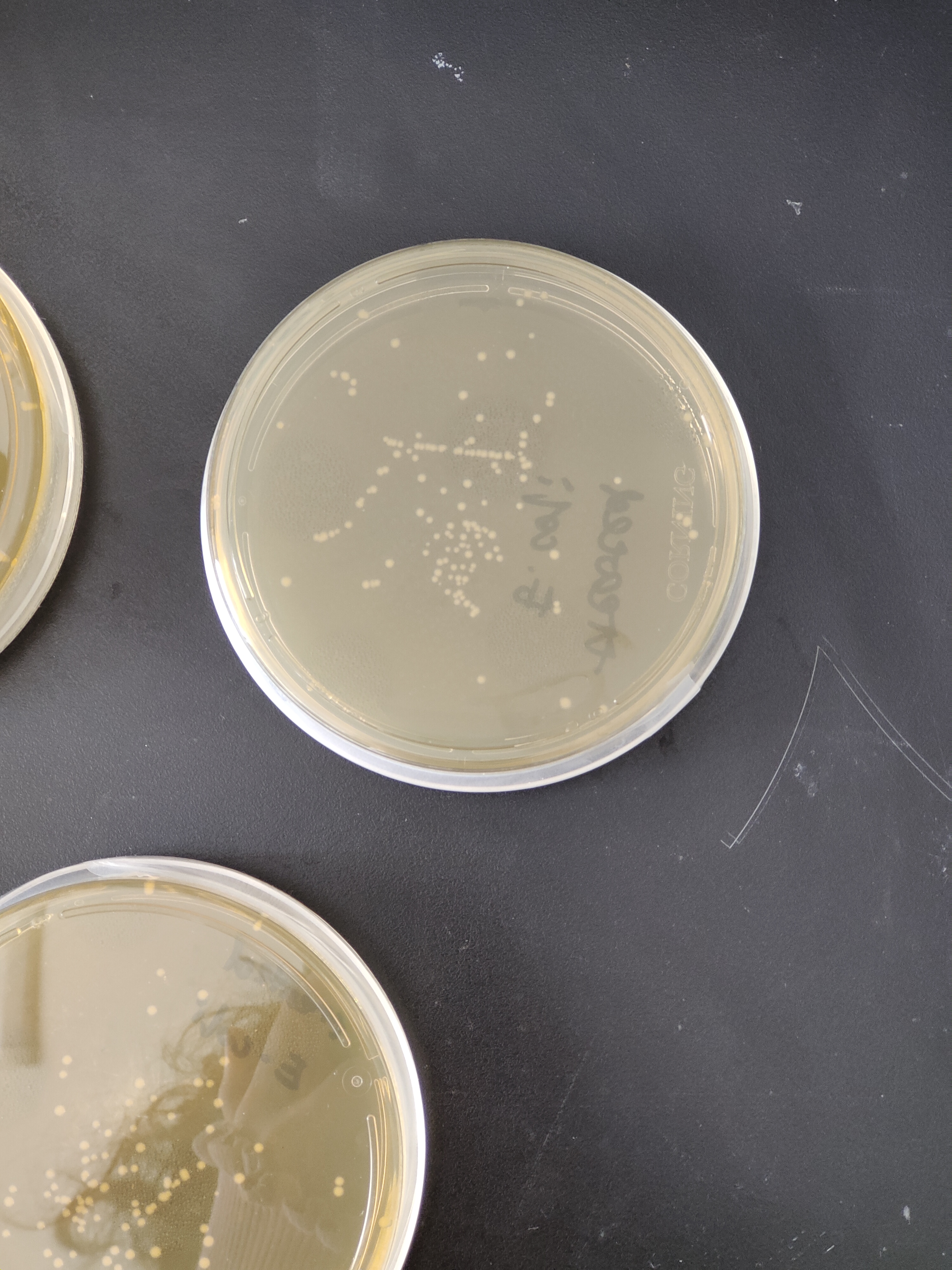

Supplement: Supplementary file 4 — Source Data [file 41467_2022_28344_MOESM4_ESM.zip › Source Data_NCOMMS-21-01890A/Fig. S29/E. coli/treated-4.jpg]

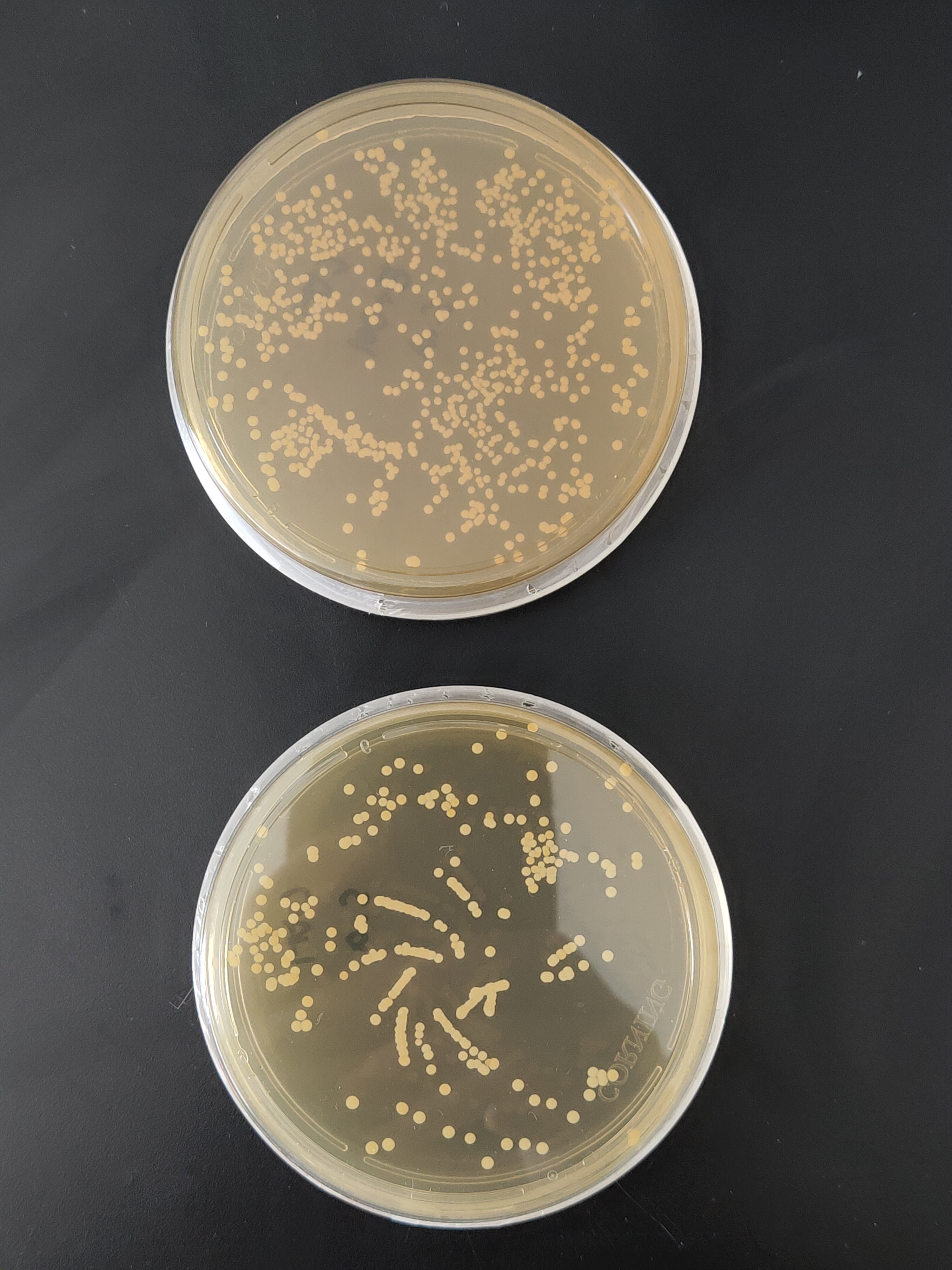

Supplement: Supplementary file 4 — Source Data [file 41467_2022_28344_MOESM4_ESM.zip › Source Data_NCOMMS-21-01890A/Fig. S29/S. aureus/ctrl-1.jpg]

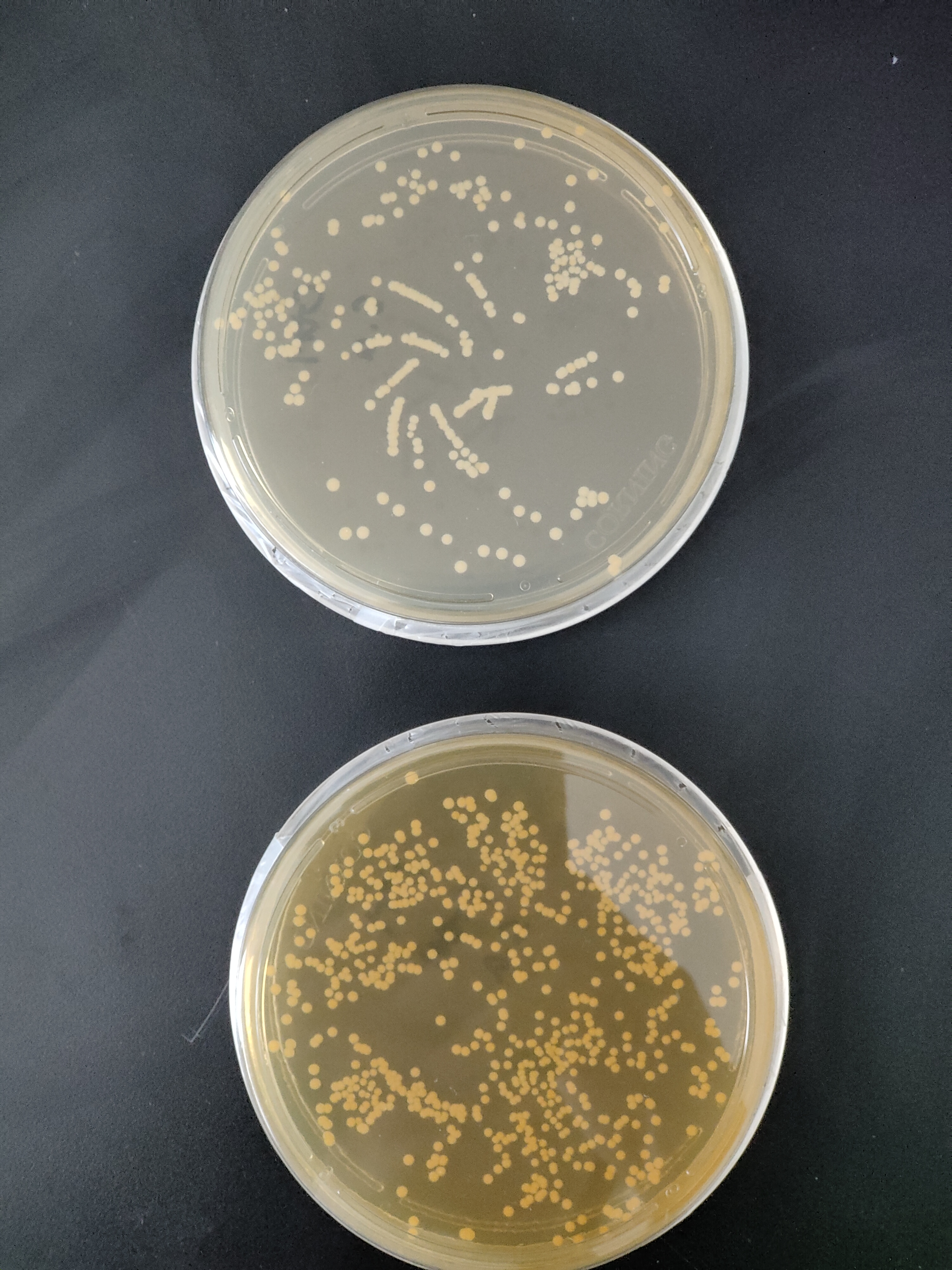

Supplement: Supplementary file 4 — Source Data [file 41467_2022_28344_MOESM4_ESM.zip › Source Data_NCOMMS-21-01890A/Fig. S29/S. aureus/ctrl-2.jpg]

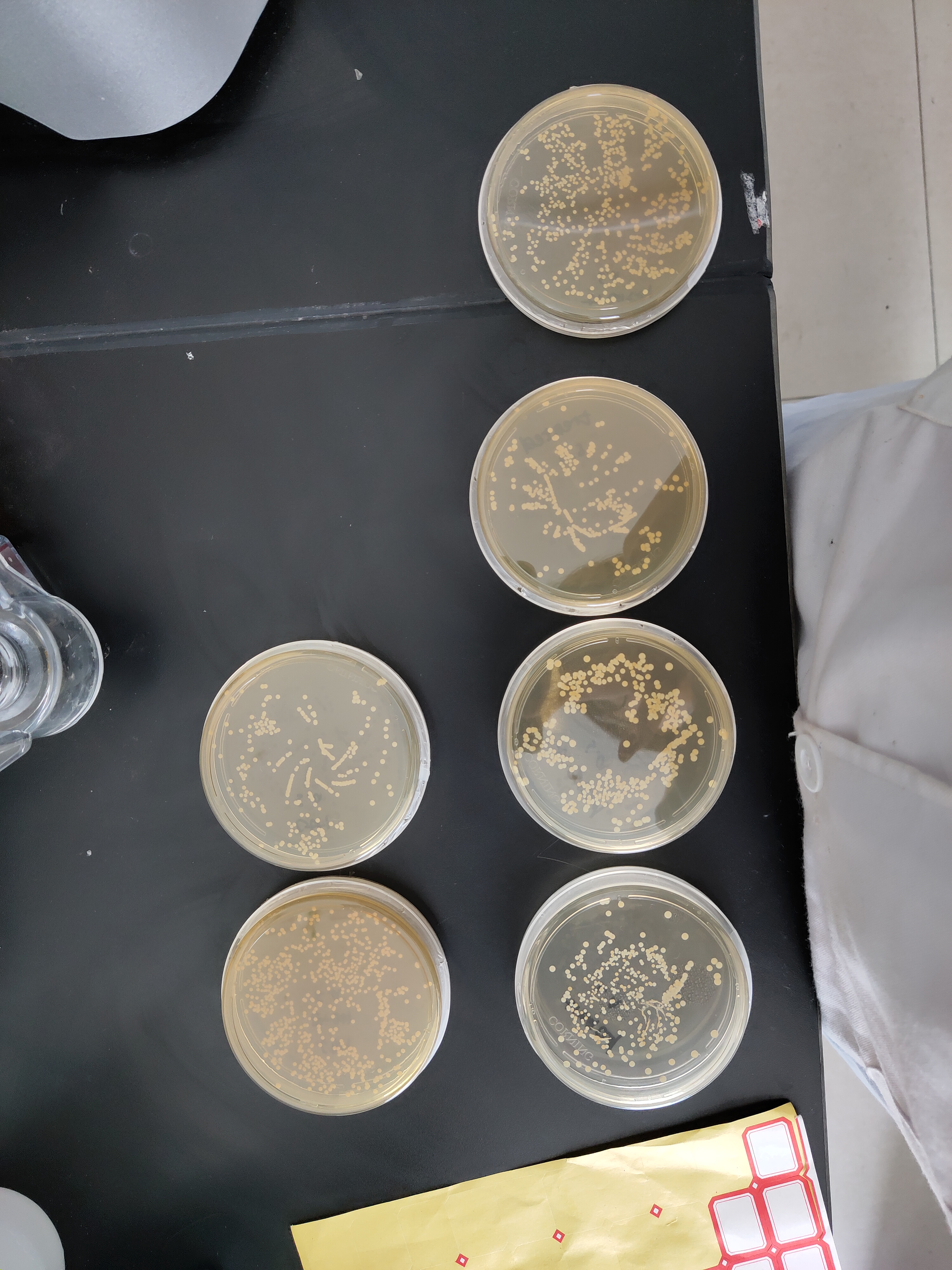

Supplement: Supplementary file 4 — Source Data [file 41467_2022_28344_MOESM4_ESM.zip › Source Data_NCOMMS-21-01890A/Fig. S29/S. aureus/overall.jpg]

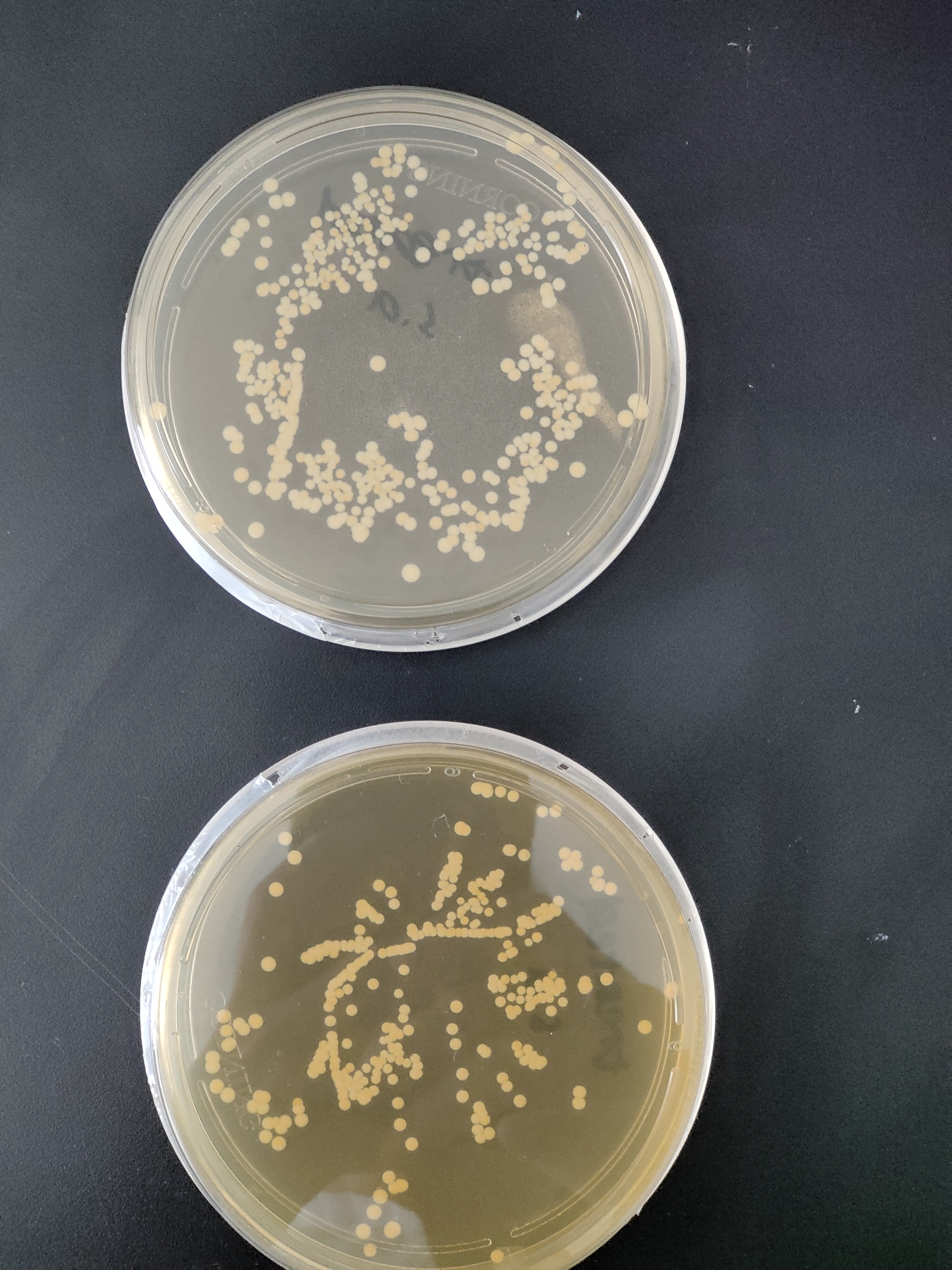

Supplement: Supplementary file 4 — Source Data [file 41467_2022_28344_MOESM4_ESM.zip › Source Data_NCOMMS-21-01890A/Fig. S29/S. aureus/treated-1.jpg]

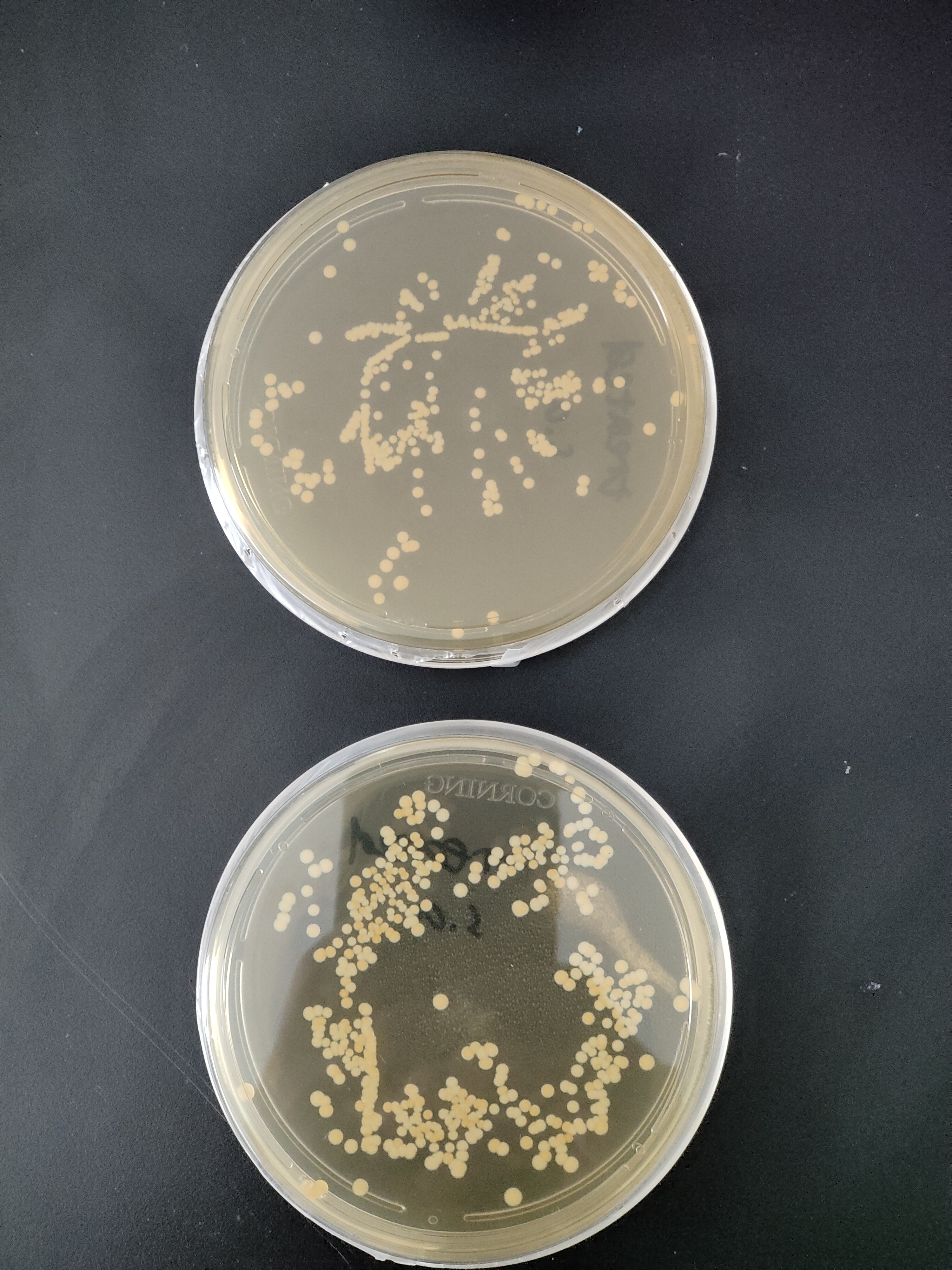

Supplement: Supplementary file 4 — Source Data [file 41467_2022_28344_MOESM4_ESM.zip › Source Data_NCOMMS-21-01890A/Fig. S29/S. aureus/treated-2.jpg]

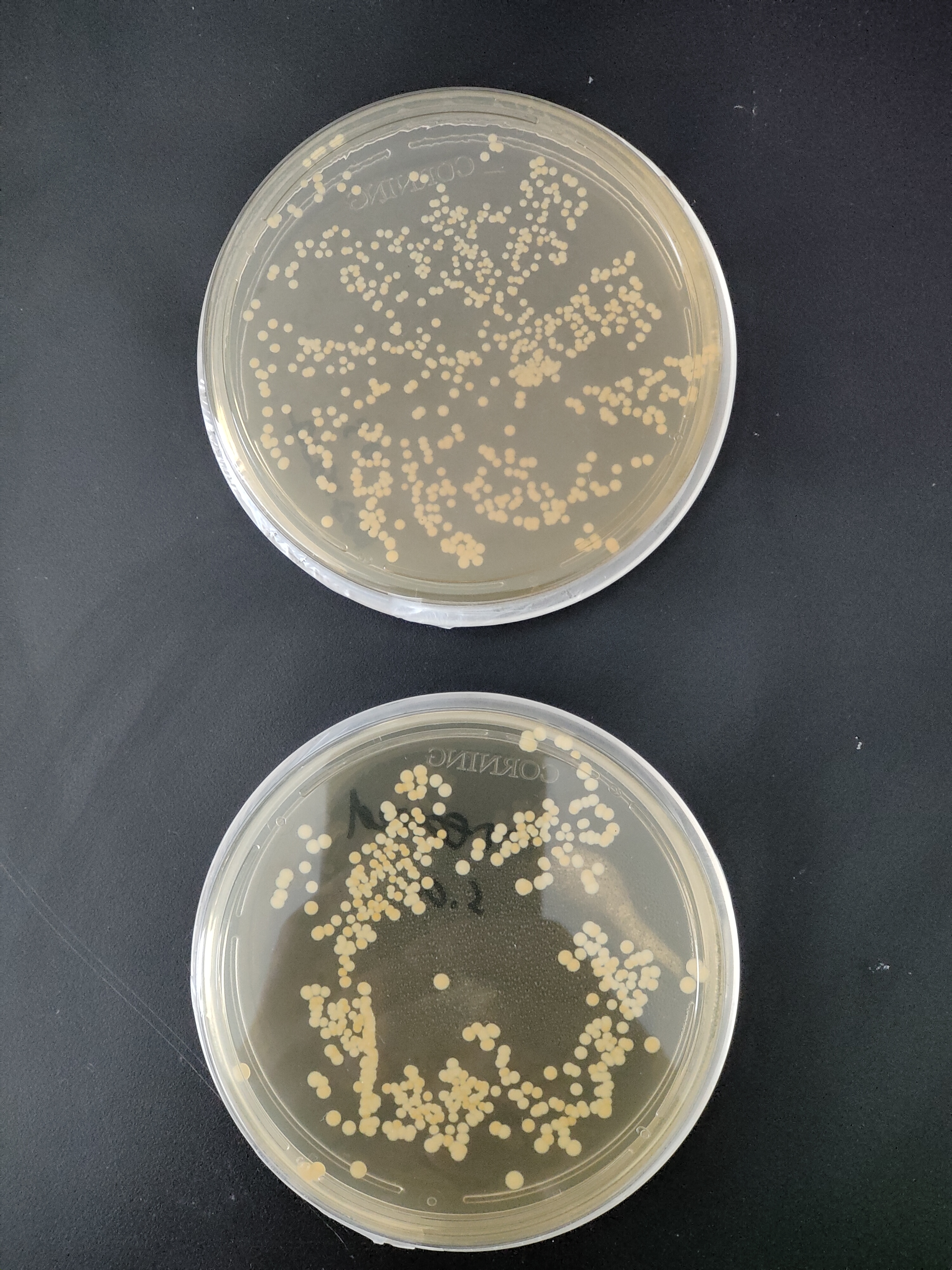

Supplement: Supplementary file 4 — Source Data [file 41467_2022_28344_MOESM4_ESM.zip › Source Data_NCOMMS-21-01890A/Fig. S29/S. aureus/treated-3.jpg]

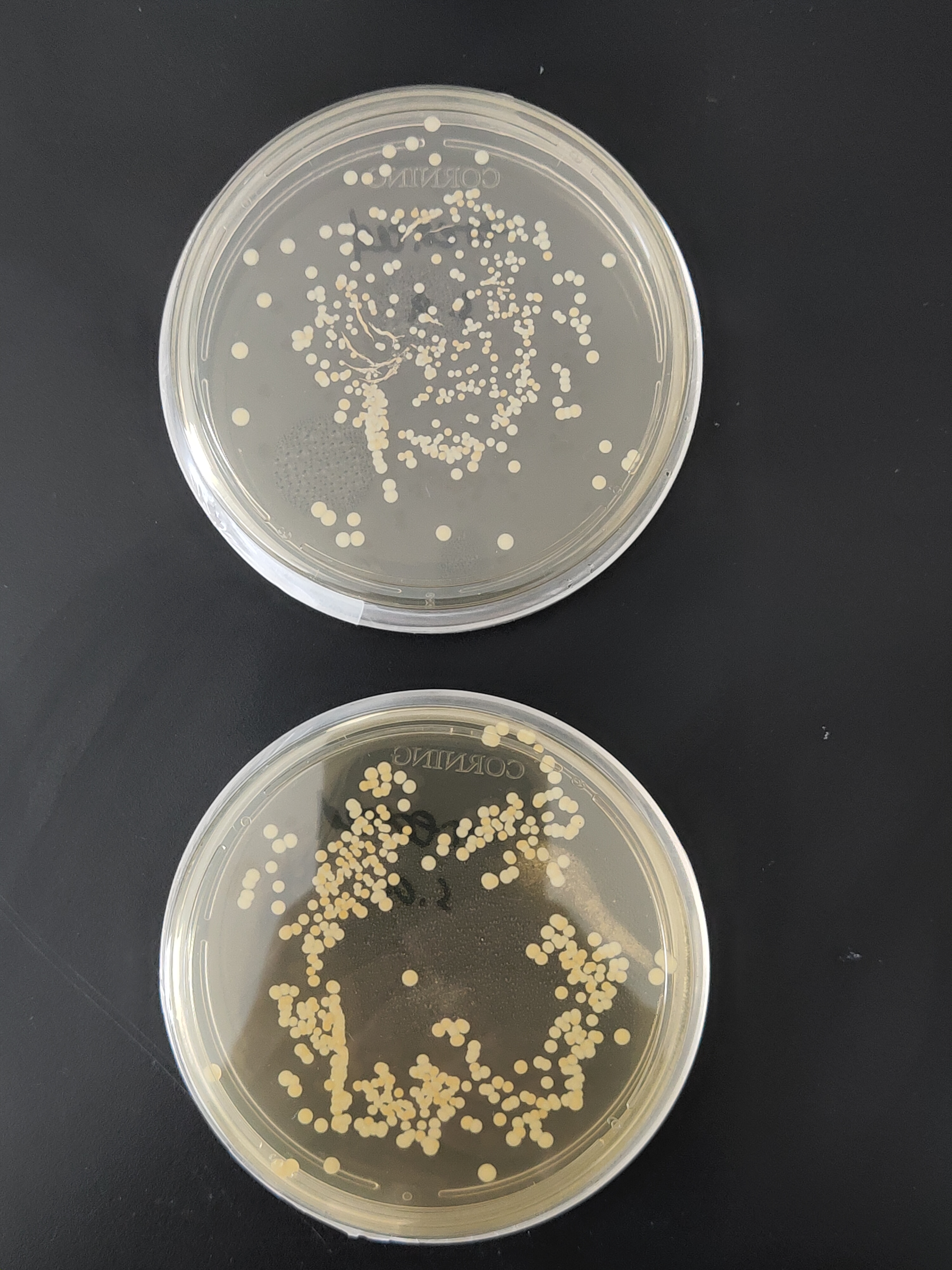

Supplement: Supplementary file 4 — Source Data [file 41467_2022_28344_MOESM4_ESM.zip › Source Data_NCOMMS-21-01890A/Fig. S29/S. aureus/treated-4.jpg]
